# Author Correction: Neoadjuvant durvalumab plus radiation versus durvalumab alone in stages I–III non-small cell lung cancer: survival outcomes and molecular correlates of a randomized phase II trial

**DOI:** 10.1038/s41467-023-44575-3

**Published:** 2024-01-03

**Authors:** Nasser K. Altorki, Zachary H. Walsh, Johannes C. Melms, Jeffery L. Port, Benjamin E. Lee, Abu Nasar, Cathy Spinelli, Lindsay Caprio, Meri Rogava, Patricia Ho, Paul J. Christos, Ashish Saxena, Olivier Elemento, Bhavneet Bhinder, Casey Ager, Amit Dipak Amin, Nicholas J. Sanfilippo, Vivek Mittal, Alain C. Borczuk, Silvia C. Formenti, Benjamin Izar, Timothy E. McGraw

**Affiliations:** 1 Weill Cornell Medicine, Department of Cardiothoracic Surgery, New York, New York USA; 2 Department of Medicine, Division of Hematology and Oncology, Columbia University Irving Medical Center, Vagelos College of Physicians & Surgeons, New York, New York USA; 3 Department of Population Health Sciences, Weill Cornell Medicine, New York, New York USA; 4 Weill Cornell Medicine, Division of Hematology and Oncology, New York, New York USA; 5 Weill Cornell Medicine, Caryl and Israel Englander Institute for Precision Medicine, Institute for Computational Biomedicine, Department of Physiology and Biophysics, New York, New York USA; 6 Weill Cornell Medicine, Department of Radiation Oncology, New York, New York USA; 7 Department of Pathology, Northwell Health, Greenvale, New York, New York USA; 8 Department of Systems Biology, Program for Mathematical Genomics, Columbia University, New York, New York USA; 9 Columbia Center for Translational Immunology, New York, New York USA; 10 Weill Cornell Medicine, Department of Biochemistry, New York, New York USA

**Keywords:** Surgical oncology, Cancer immunotherapy, Non-small-cell lung cancer, Radiotherapy

Correction to: *Nature Communications* 10.1038/s41467-023-44195-x, published online 19 December 2023

In this article, the author name Silvia C. Formenti was incorrectly written as Sylvia C. Formenti. The original article has been corrected.

