# Supplementary material for: Neoadjuvant durvalumab plus radiation versus durvalumab alone in stages I–III non-small cell lung cancer: survival outcomes and molecular correlates of a randomized phase II trial
Source: Nat Commun. 2023 Dec 19;14:8435. doi: 10.1038/s41467-023-44195-x (PMC10730562; doi:10.1038/s41467-023-44195-x)
Supplement: Supplementary file 1 — Supplementary Information [file 41467_2023_44195_MOESM1_ESM.pdf]

Table S1: Adjuvant therapy in 52 resected patients

	Monotherapy	SBRT + durvalumab
Chemotherapy+/- radiation	16	9
Median cycles	4	4
Radiotherapy alone	1	2
Adjuvant durvalumab	16	18
Median cycles	8	9

Table S2. Antibodies used for flow.

Antigen	Fluorophore	Antibody Clone	Company & Catalog Number	Antibody Dilution (empirically determined)
CD8	BV421	SK1	Biolegend 344748	1 to 100
HLA-DR	eF450	LN3	Thermo 48-9956-42	1 to 400
CD4	BV480	SK3	BD 566104	1 to 100
Ki67	eF506	SolA15	Invitrogen 69-5698-82	1 to 100
CD95	BV510	DX2	Biolegend 305640	1 to 100
LAG3	BV605	T47-530	BD 745160	1 to 100
CD137 (4-1BB)	BV650	4B4-1	Biolegend 309828	1 to 100
CD45RA	BV711	HI100	Biolegend 304137	1 to 400
CD28	BV750	CD28.2	Biolegend 302969	1 to 50
CD103	BV785	Ber-ACT8	BD 568274	1 to 50
TCF1	A488	C63D9	Cell Signaling 6444S	1 to 50
CD39	PE	A1	Biolegend 328208	1 to 50
FoxP3	PE-Dazzle 594	206D	Biolegend 320125	1 to 50
CD27	PE-Cy5	O323	Thermo 15-0279-42	1 to 50
KLRG1	PerCP-Cy5.5	SA231A2	Biolegend 367708	1 to 50
TIM3	PE-Cy7	F38-2E2	eBioscience 25-3109-42	1 to 50
TOX1	APC	REA473	Miltenyi 130-118-335	1 to 100
NKG2D	A660	1D11	Biolegend 320842	1 to 100
CD3	A700	HIT3a	Biolegend 300324	1 to 100
CCR7	APC-Fire750	G043H7	Biolegend 353246	1 to 50
PD1	APC-Fire810	RA3-6B2	Biolegend 612620	1 to 50
Viability Dye	Zombie NearIR	n/a	Biolegend 423106	1 to 500

Figure S1: Consort Diagram

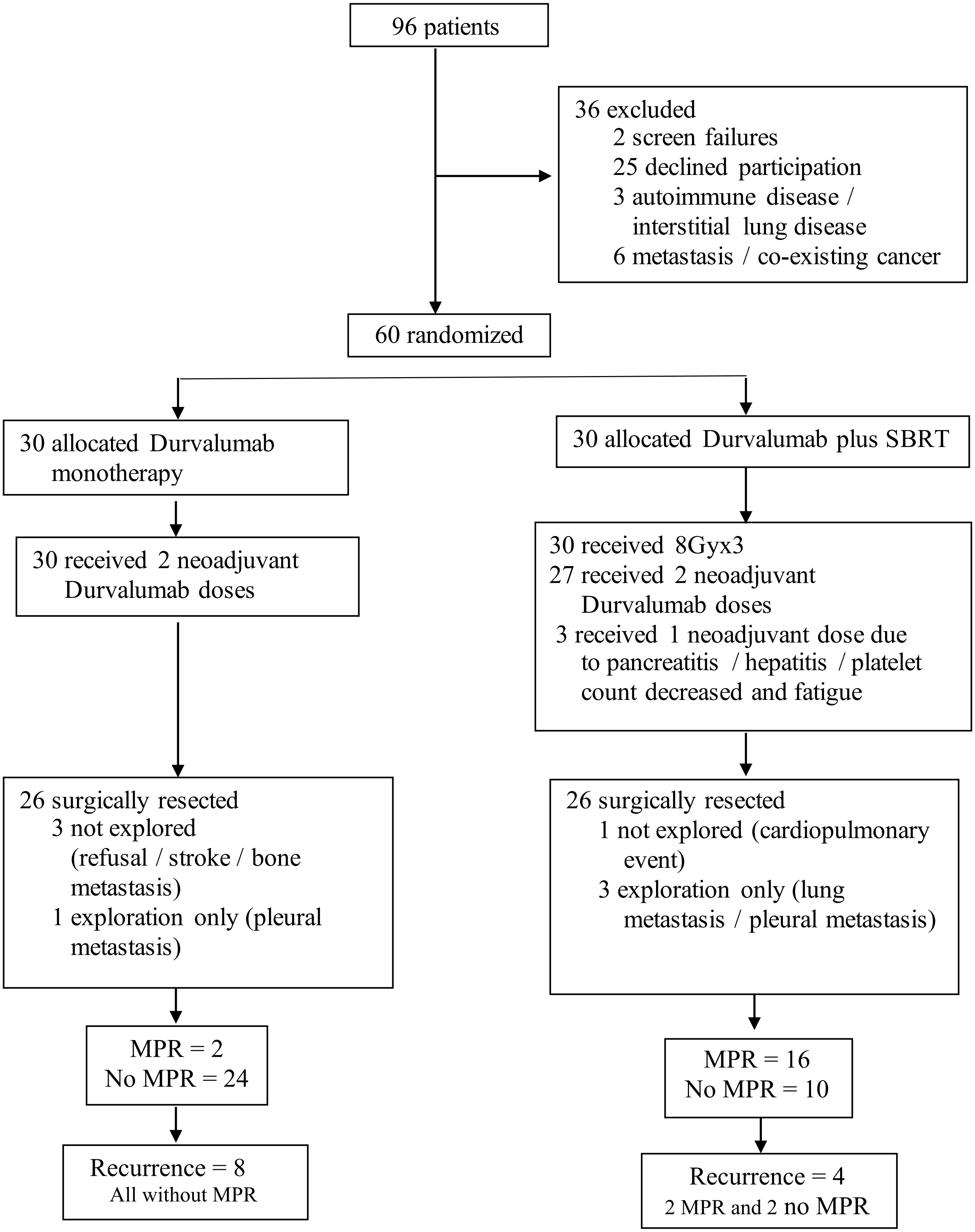

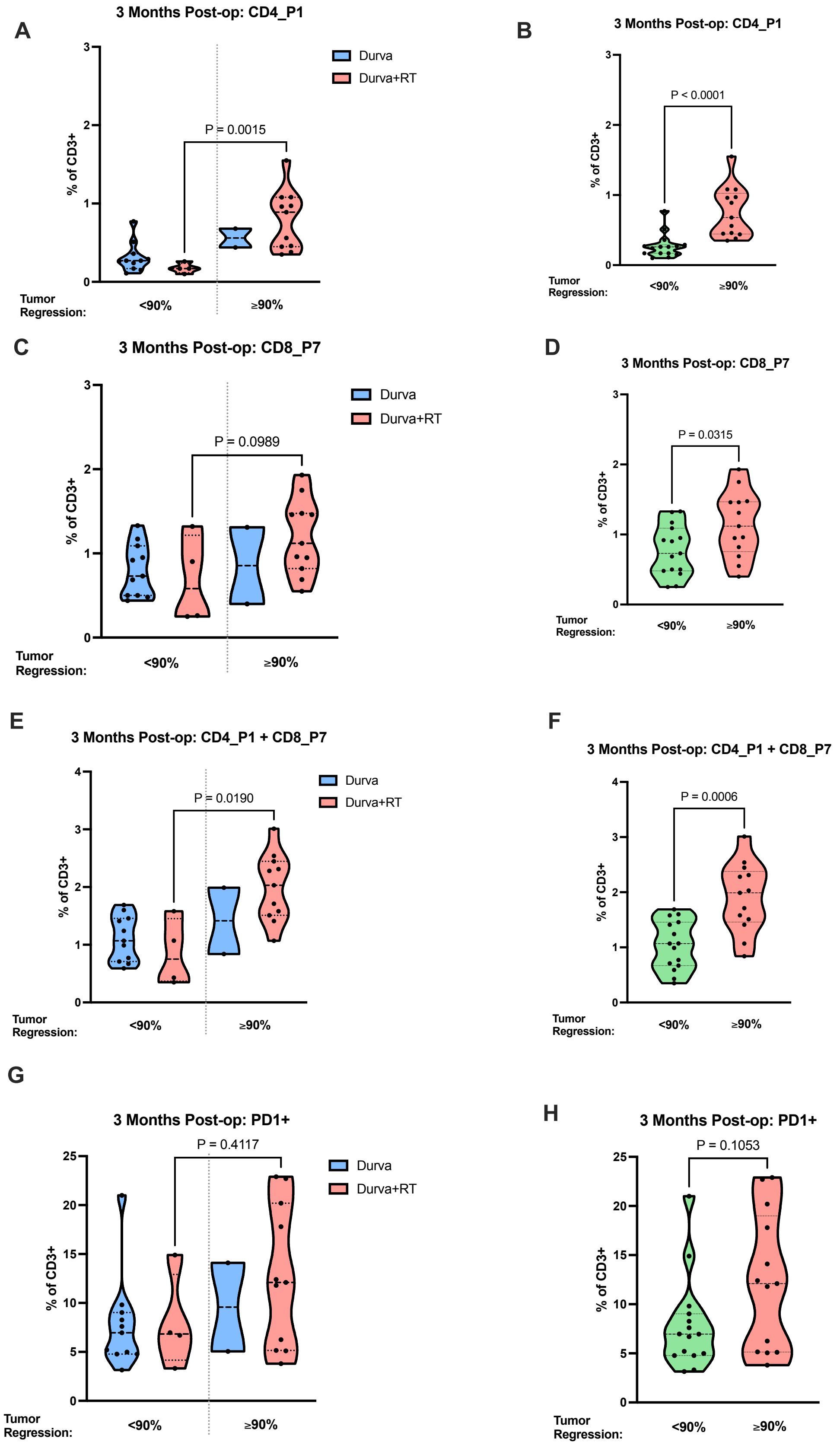

Figure S2. Association of 3 months post-operative CD103+ and PD1+ T cell population frequencies with MPR. Patient post-op peripheral blood samples were evaluated for the frequency of circulating **A-B.** CD4_P1 (CD103-expressing) T cells, **C-D.** CD8_P7 (CD103-expressing) T cells, **E-F.** Combined frequency of CD4_P1 and CD8_P7 T cells, and **G-H.** PD1^{+} T cells**. A-H: T**wo-sided Mann-Whitney test. A, C, E & G: Durva <90% (n=11), Durva+SBRT <90% (n=4), Durva ≥ 90% (n=2), Durva+SBRT >90% (n=11). B,D,F & H: < 90% (n=15), ≥ 90% (n=13). Source data for all panels are provided as a Source Data file.

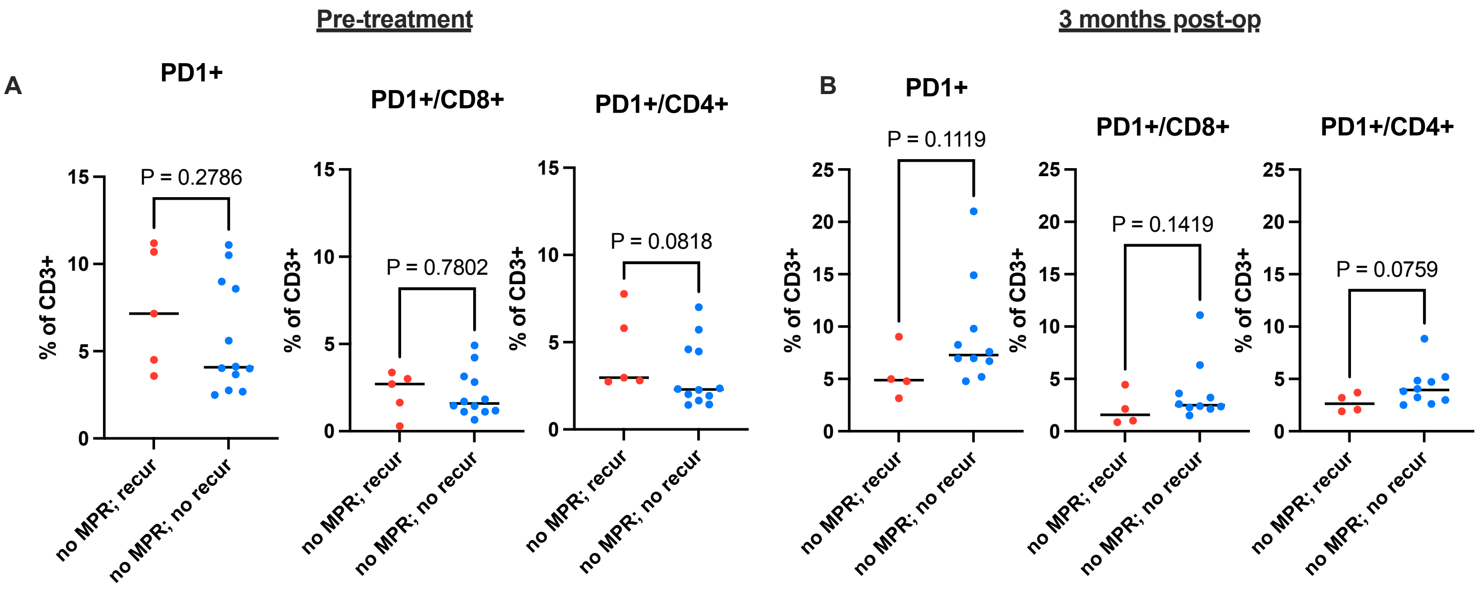

Figure S3. Association of circulating T cell populations with long-term freedom from recurrence in patients who did not experience MPR. Patients who did not experience MPR were compared for the frequency of circulating $PD1^{+}$ T cell populations at **A:** pre-treatment and **B:** 3 months post-operative timepoints. **A-B:** Two-sided Mann-Whitney test. Source data for all panels are provided as a Source Data file. A: all panels, no MPR, no recur (n=5), no MPR, no recur (n=12). B: all panels no MPR, no recur (n=4), no MPR, no recur (n=10).

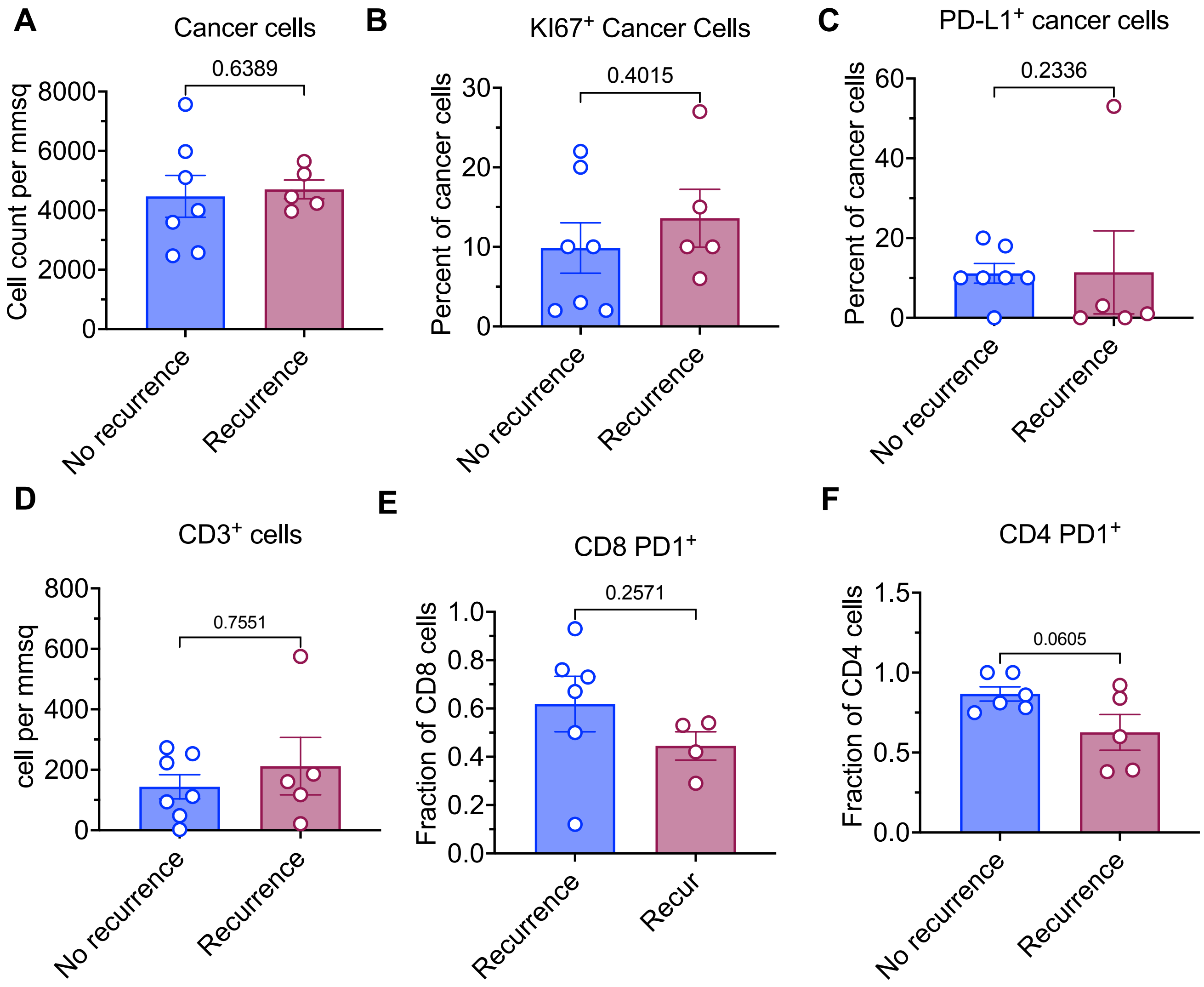

Figure S4. Immune profiling of resected tumors by multiplex immune fluorescence. **A.** Cancer cells (panCK$^+$) per mmsq. No Recurrence (n=7), Recurrence (n=5). Mean ± SEM. Two-sided, unpaired Mann-Whitney test. **B.** ki67$^+$ cancer cells as percent of total cancer cells. No Recurrence (n=7), Recurrence (n=5). Mean ± SEM. Two-sided, unpaired Mann-Whitney test. **C.** PD-L1$^+$ cancer cells as percent of total cancer cells. No Recurrence (n=7), Recurrence (n=5). Mean ± SEM. Two-sided, unpaired Mann-Whitney test. **D.** CD3$^+$ cells per mmsq. No Recurrence (n=7), Recurrence (n=5). Mean ± SEM. Two-sided, unpaired Mann-Whitney test. **E.** CD8$^+$ PD1$^+$ cells as a fraction of total CD8$^+$ cells. No Recurrence (n=7), Recurrence (n=4). Mean ± SEM. Two-sided, unpaired Mann-Whitney test. **F.** CD4$^+$ PD1$^+$ cells as a fraction of total CD4$^+$ cells. No Recurrence (n=7), Recurrence (n=5). Mean ± SEM. Two-sided, unpaired Mann-Whitney test. Source data for all panels are provided as a Source Data file.

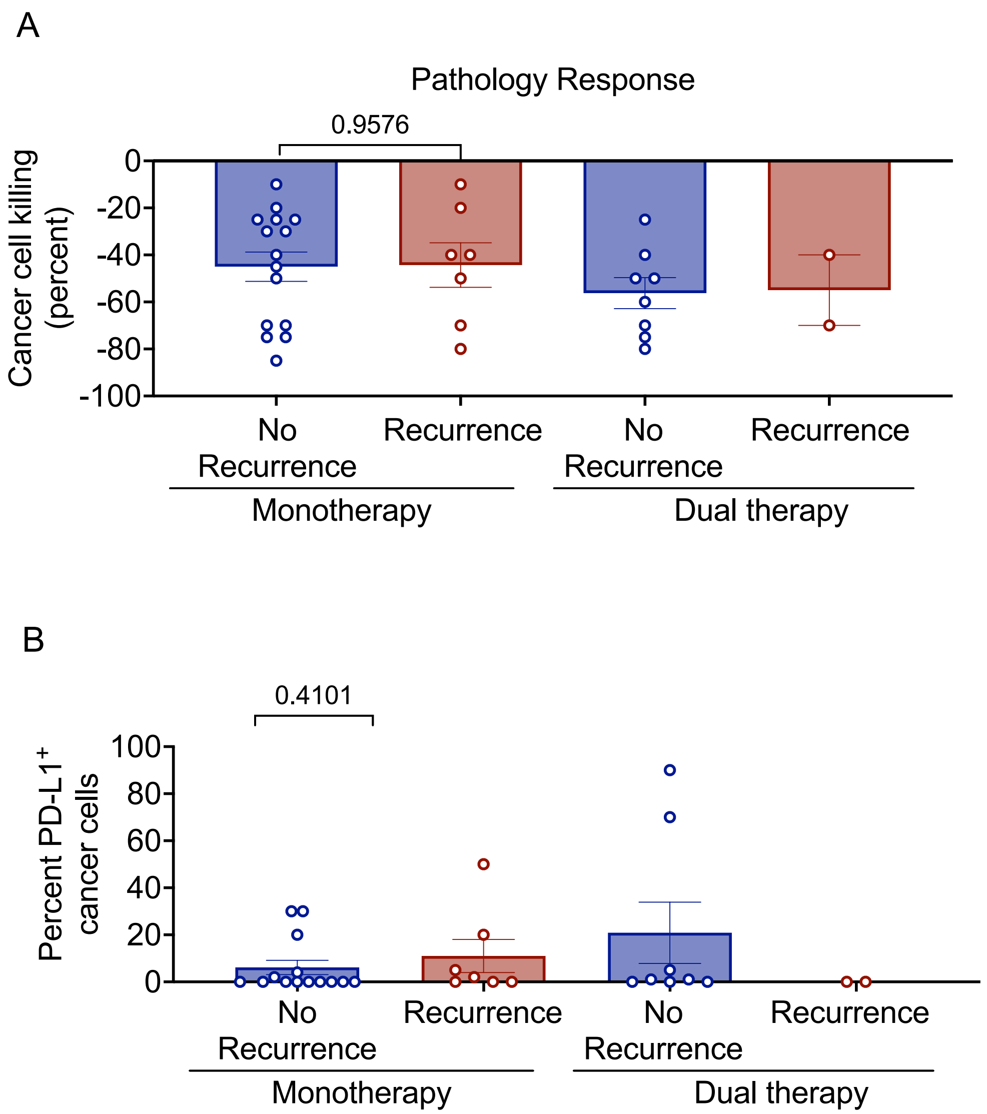

Figure S5. Pathology response and PD-L1 expression post therapy. A. Pathology response by trial arm and recurrence. Mean ± SEM. Monotherapy: No recurrence (n=15), Recurrence (n=7). Dual therapy: No recurrence (n=8), Recurrence (n=2). Two-sided, unpaired Mann-Whitney test. Too few data points for recurrence in dual therapy to perform statistical test. **B.** Cancer cell PD-L1 expression. Mean ± SEM. Monotherapy: No recurrence (n=15), Recurrence (n=7). Dual therapy: No recurrence (n=8), Recurrence (n=2). Two-sided, unpaired Mann-Whitney test. Too few data points for recurrence in dual therapy to perform statistical test. Source data for all panels are provided as a Source Data file.

Dimensionality Reduction Analysis: T cell panel

QC Removal of Samples (<10% viability or <2000 live cells)

↓

Manual gating to identify live CD3+ cells

↓

Downsample and concatenate live CD3+

↓

Manual gating of concatenated sample for CD4+ and CD8+ T cell subsets

↓

PaCMAP dimensionality reduction of CD8+ and CD4+ T cell subsets → FlowSOM unbiased delineation of CD8+ and CD4+ subclusters → Phenotyping and per-sample frequency analysis of subclusters

Manual Gating Scheme: T cells

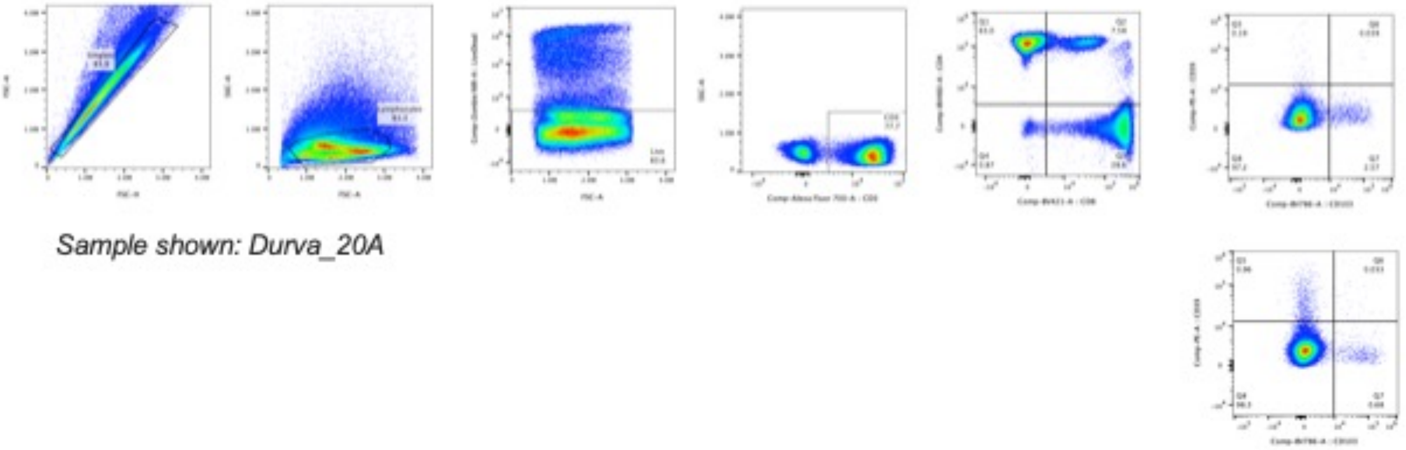

Sample shown: Durva_20A

Figure S6. Flow strategy and exemplary gating

IND Number: 132371
IRB protocol # 1501015795

A randomized phase 2 trial of Durvalumab (MEDI4736) with or without SBRT in clinical stage I, II, and IIIA non-small cell lung cancer (NSCLC)

Weill Cornell Medical College of Cornell University
The Sandra and Edward Meyer Cancer Center

New York, NY

Nasser Altorki, M.D. (Thoracic Surgery)
Brendon Stiles, M.D. (Thoracic Surgery)
Jeffrey Port, M.D. (Thoracic Surgery)
Ronald Scheff, M.D. (Medical Oncology)
Ashish Saxena, M.D. (Medical Oncology)
Sylvia Formenti, M.D. (Radiation Oncology)
Sandra Demaria, M.D. (Radiation Oncology)
Bradley Pua, M.D. (Radiology)
Ben Lee,M.D. (Thoracic Surgery)
Alan Borczuk, M.D. (Pathology)
Vivek Mittal, Ph.D. (Thoracic Surgery/Cell Biology)
Tim McGraw, Ph.D. (Biochemistry)
Karla Ballman Ph.D. (Biostatistics)
Paul Christos, Dr. P.H., M.S. (Biostatistics)
Eugene Shostak, M.D. (Thoracic Surgery)
Nicholas Sanfilippo, MD (Radiation Oncology)
Giorgio Inghirami, M.D. (Pathology)

Version number 1	January 28, 2015
Version number 2	February 29, 2016
Version number 3	June 15, 2016
Version number 4	October 11, 2016
Version number 5	May 24, 2017
Version number 6	December 19, 2017
Version number 7	March 6, 2018
Version number 8	October 1, 2018
Version number 9	February 6, 2019
Version number 10	February 13, 2020
Version number 11	July 14, 2020

Table of Contents

Table of Contents 2
SCHEMA 4
1.0 OBJECTIVES 4
1.1 Primary Objective 4
1.2 Secondary Objectives 4
1.3 Exploratory Objective 4
1.4 Rationale and Hypothesis 4
1.4.1 Targeting PD-L1 is a safe and promising strategy 5
1.4.2 Non-ablative doses of radiation will potentiate anti-PD-L1 therapy in PD-L1 +ve and –ve tumors 5
1.4.3 Hypothesis 6
2.0 BACKGROUND 6
2.1 Cancer and immune system function 6
2.2 Durvalumab in Solid Tumors 7
2.2.1 Preclinical overview 7
2.2.2 Durvalumab Clinical Review 8
2.2.3 Pharmacokinetics and Product Metabolism 8
2.2.4 Safety 9
2.2.5 Efficacy 11
2.2.6 Targeting the PD1/PD-L1 pathway in NSCLC 13
2.2.7 Radiation and tumor immunity 14
2.2.8 Radiation combined with immune checkpoint inhibition 15
3.0 PATIENT SELECTION 16
3.1 Inclusion criteria 16
3.2 Exclusion criteria 17
4.0 REGISTRATION AND RANDOMIZATION PROCEDURES 18
4.1 Registration 18
4.2 Randomization 19
5.0 STUDY DESIGN 19
5.1 Treatment Plan 19
5.2 Limiting normal tissue toxicities with non-ablative SBRT 19
5.3 Duration of follow-up 20
5.4 Withdrawal of Subjects from Study Treatment and/or Study 20
5.4.1 Permanent discontinuation of Durvalumab 20
5.4.2 Withdrawal of consent 21
5.5 Study Procedures 22
6.0 DOSE DELAYS/DOSE MODIFICATIONS. 28
6.1 Dose Modification and Toxicity Management 28
6.2 Study restrictions and Concomitant Medications 28
6.2.1 Contraception 28
6.2.2 Blood donation 29
6.2.3 Permitted Concomitant Medications 29
6.2.4 Excluded Concomitant Medications 30
7.0 ASSESSMENT OF SAFETY. 31
7.1 Safety Parameters 31
7.1.1.1 Definition of Adverse Events 31
7.1.2 Definition of Serious Adverse Events 31
7.1.3 Definition of Adverse Events of Special Interest (AESI) 31
7.1.4 Pneumonitis 32

7.1.5 Hypersensitivity reactions ... 32
7.1.6 Hepatic function abnormalities (hepatotoxicity) ... 33
7.1.7 Gastrointestinal disorders ... 33
7.1.8 Endocrine disorders ... 33
7.1.9 Pancreatic disorders ... 34
7.1.10 Neurotoxicity ... 34
7.1.11 Nephritis ... 34
7.1.12 Radiation risks. ... 34
7.2 Assessment of safety parameters ... 34
7.2.1 Adverse event characteristics ... 34
7.3 Recording of adverse events ... 34
7.3.1 Study recording period and follow-up for AEs and SAEs ... 35
7.3.2 Serious Adverse Event (SAE) Reporting ... 35
7.3.3 Reporting of SAE to IRB ... 35
7.3.4 Reporting of SAE to FDA and Sponsor ... 36
7.3.5 Reporting of deaths ... 36
7.3.6 Other events requiring reporting ... 37
7.3.7 Overdose ... 37
7.3.8 Hepatic function abnormality ... 37
7.3.9 Pregnancy ... 37
7.3.10 Paternal ... 38
7.4 Data Safety Monitoring Plan ... 38
8.0 PHARMACEUTICAL INFORMATION ... 38
8.1 Investigational Agent ... 38
8.1.1 Durvalumab ... 38
8.1.2 Formulation/packaging/storage ... 38
8.1.3 Study drug preparation ... 38
8.1.4 Monitoring of dose administration ... 40
8.1.5 Agent Accountability ... 40
9.0 MEASUREMENT OF EFFECT ... 40
9.1 Assessment of disease-free survival ... 40
9.2 Assessment of clinical response ... 40
9.3 Assessment of pathological response ... 44
10.0 DATA REPORTING / REGULATORY CONSIDERATIONS ... 41
10.1 Data Collection ... 41
10.1.1 REDCap ... 41
10.2 Regulatory Considerations ... 41
10.3 Ethical conduct of the study ... 41
11.0 STATISTICAL CONSIDERATIONS ... 41
11.1 Specific Aims ... 41
11.1.1 Primary Aim ... 41
11.1.2 Secondary Aim ... 42
11.2 Analysis Plan for Endpoints ... 42
11.2.1 Primary Endpoint ... 42
11.2.2 Secondary Endpoints ... 42
REFERENCES ... 44
APPENDICES ... 49
APPENDIX A: Durvalumab Dose Calculations ... 49
APPENDIX B: Dosing Modifications and Toxicity Management Guidelines ... 50
APPENDIX C WCMC SAE Reporting Form ... 84

SCHEMA

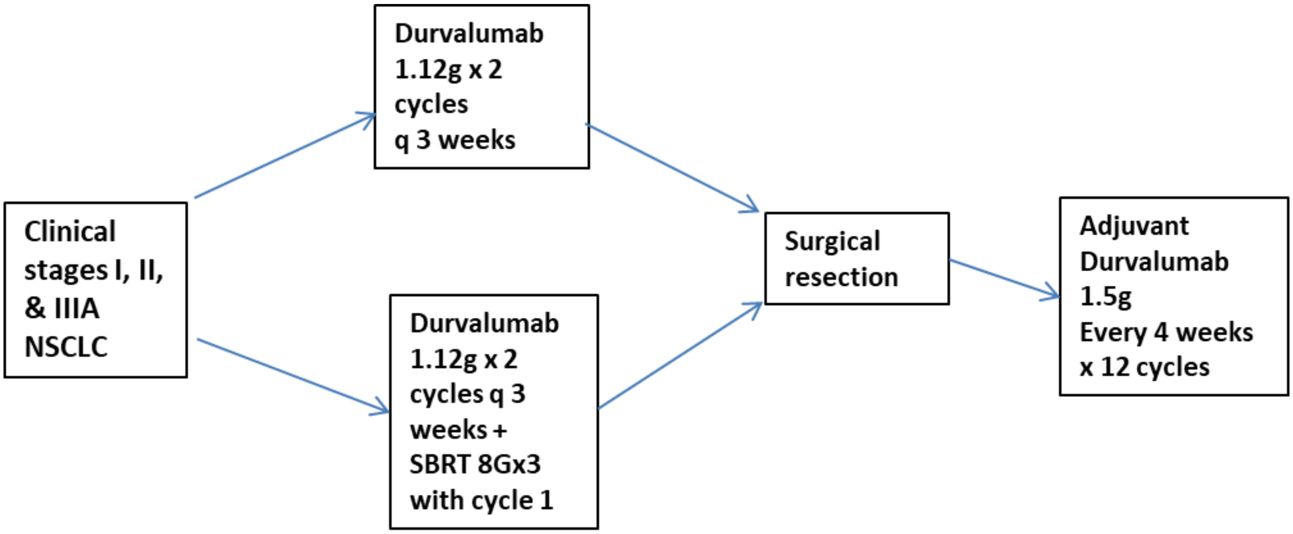

1.0 OBJECTIVES

1.1 Primary Objective

- To compare the pathological response rate after 2 cycles of anti-PD-L1 monotherapy and anti-PD-L1 with SBRT.

1.2 Secondary Objectives

- To determine whether pre-operative anti-PDL-1 therapy with or without radiation leads to an improvement in DFS in patients with clinical stage I (tumors > 2 cm), II, and IIIA NSCLC from a historical control rate of 75% at 2 years to 89%.
- To determine if non-ablative doses of radiotherapy given concurrently with neo-adjuvant durvalumab will lead to a clinical response rate of 35% compared to a clinical response rate of 15% predicted in the antibody alone arm. The response rate of 15% selected for the antibody alone arm is consistent with response rates with durvalumab in patients with advanced refractory lung cancer.
- To determine the safety of 2 cycles of preoperative durvalumab with and without radiation followed by surgery and adjuvant durvalumab every 4 weeks for 12 cycles post-operatively.

1.3 Exploratory Objective

- To determine whether the improvement in response rate in the radio-immunotherapy arm will translate into an improvement in DFS. Though the trial is not powered to adequately test this hypothesis, it may generate data suggesting future trial designs

1.4 Rationale and Hypothesis

Approximately 20-25% of the 220,000 patients diagnosed with NSCLC in the United States in 2014 will have early stage disease (stages I-IIIA) [1]. Although surgical resection is the treatment of choice, five year overall survival is only 55% and disease-free survival (DFS) maybe as low as 40% at five

years. Over the past decade three large international randomized trials have shown that adjuvant chemotherapy after complete resection leads to a significant improvement in survival [2-5]. A meta-analysis of all randomized trials confirmed these results and showed that the absolute improvement in 5-year survival after chemotherapy is only 5.4% [5]. Despite this statistically significant improvement in survival, 40-50% of patients develop locally recurrent or metastatic disease [6, 7]. More recently, large international adjuvant trials tested the efficacy of targeted therapies such as the anti-VEGF antibody bevacizumab or the EGFR tyrosine kinase inhibitor, erlotinib, after surgical resection [8, 9]. Both trials did not meet their primary endpoint of improved DFS. Similarly, a large international randomized trial using vaccination with the recombinant MAGE-A3 protein in patients with MAGE-A3 positive tumors failed to meet the primary endpoint of improving DFS[10]. Clearly there is an urgent unmet clinical need to develop novel neo-adjuvant or adjuvant therapies for patients with early stage lung cancer.

1.4.1 Targeting PD-L1 is a safe and promising strategy

Several clinical trials in patients with advanced refractory NSCLC have shown that antibodies targeting the PD1/PD-L1 pathway resulted in clinical response rates in approximately 15-25% of patients with advanced chemo-refractory NSCLC[11-18]. Responses occurred in patients with either squamous or non-squamous cancers and in patients with and without mutations or rearrangements in driver oncogenes. Although responses were more frequently seen in patients whose tumors expressed PD-L1, responses were also observed in patients with PD-L1 negative tumors. Unlike patients treated by chemotherapy or targeted agents, responses achieved by targeting these inhibitory checkpoints were durable in over 80% of responders and occasionally response was maintained even after drug discontinuation. The favorable safety profile and impressive response rates of these antibodies in advanced disease strongly justify evaluation of their efficacy in patients with early stage disease. However, the more frequent association of anti-PD-1 antibodies with grades 3/4 pneumonitis may limit the utility of anti-PD-1 agents in the neoadjuvant setting in patients with early stage lung cancer. In contrast, anti-PD-L1 antibodies appear to be associated with a more favorable safety profile and to-date have rarely been associated with pneumonitis grade 3 or above. Therefore, we anticipate that neo-adjuvant anti-PD-L1 therapy in early stage NSCLC will be safe and well tolerated. Beyond safety concerns, recent evidence from ongoing neoadjuvant trials in early stage NSCLC shows an unexpectedly high rate of major and complete pathological response rate (20-45%) than would have been anticipated from response rates from trials of immune-check point inhibitors (ICIs) in advanced disease. The association between pathological response and survival has been clearly shown following neoadjuvant therapy for breast cancer[70]. An association between pathological response rate and survival has not been definitively shown in NSCLC. An an ongoing international project that investigates pathological response as a surrogate endpoint for survival in the context of neoadjuvant ICIs in early stage lung cancer has been initiated by the International Association for Study of Lung Cancer (IASLC) .

1.4.2 Non-ablative doses of radiation will potentiate anti-PD-L1 therapy in PD-L1 +ve and –ve tumors

The immuno-modulatory effects of radiation cited below and the results of preclinical studies combining radiation with immune checkpoint inhibitors strongly suggest that combination radio-immunotherapy may potentiate the anti-tumor effects of PD-L1 blockade in PD-L1 positive tumors [19-22]. Furthermore, the induction of PD-L1 expression in tumor and immune cells by radiation may render PD-L1 negative tumors more susceptible to anti-PD-L1 therapy[23]. Given the emerging data on the high pathological response rates from following neoadjuvant ICIs in early NSCLC[71], we postulate that the addition of subablative SBRT to ICI will significantly increase the rate of pathological response compared to ICI alone.

1.4.3 Hypothesis

In this randomized trial design we will test the hypothesis that non-ablative doses of radiation delivered in combination with PDL-1 blockade will lead to a significant improvement in pathological response rates compared to preoperative anti-PD-L1 alone and provide a hint whether such improvement in response translates to improved survival. We will also test the hypothesis that neoadjuvant anti-PDL-1 will significantly improve DFS compared to reported and institutional historical controls. The demonstration that non-ablative doses of radiation can be a potent immuno-modulator will be a paradigm shift and will set the stage for further evaluation of this strategy in NSCLC and other solid tumors.

2.0 BACKGROUND

2.1 Cancer and immune system function

Immune responses directed against tumors are one of the body's natural defenses against the growth and proliferation of cancer cells. However, over time and under pressure from immune attack, cancers develop multiple strategies to evade immune-mediated killing allowing tumor growth and progression. One such mechanism involves upregulation of surface proteins that deliver inhibitory signals to cytotoxic T cells. Programmed cell death ligand 1 is one such protein, and is upregulated in a broad range of cancers with a high frequency. In a number of these cancers, including lung [24], kidney [25-27], pancreatic [28-30], and ovarian cancer [31], tumor cell expression of PD-L1 is associated with reduced survival and an unfavorable prognosis. For example, in ovarian cancer, the 5-year survival rate in patients with low expression of PD-L1 was 80.2% compared to 52.6% in patients with high expression levels of PD-L1 [31].In lung cancer Mu *et al* reported that only 20% of patients with tumors expressing PD-L1 survived for more than 3 years compared to 49% of patients with tumors lacking PD-L1 expression [24]. However that association of PD-L1 expression with survival in lung cancer is inconsistent.

Programmed cell death ligand 1 is part of a complex system of receptors and ligands that are involved in controlling peripheral T-cell activation. PD-L1 acts at multiple sites in the body to help regulate normal immune responses and its expression by tumors is one mechanism of innate or acquired tumor immune resistance. In the lymph nodes, PD-L1 on antigen-presenting cells binds to PD-1 or CD80 on activated T cells and delivers an inhibitory signal to the T cell [32, 33]. This results in reduced T-cell activation and fewer activated T cells in circulation. In the tumor microenvironment, PD-L1 expressed on tumor cells binds to PD-1 and CD80 on activated T cells reaching the tumor. This delivers an inhibitory signal to those T cells, preventing them from killing target cancer cells and protecting the tumor from immune elimination [34].

Based on in vitro studies, an antibody that blocks the interaction between PD-L1 and its receptors can relieve PD-L1-dependent immunosuppressive effects and enhance the cytotoxic activity of antitumor T cells [35]. The levels of tumor-infiltrating lymphocytes, and more specifically cytotoxic T cells, have been correlated with improved prognosis in a number of cancers including colorectal, melanoma, and lung [36], suggesting that an antitumor immune response is beneficial to patients. Based on these findings, an anti-PD-L1 antibody could be used therapeutically to enhance antitumor immune responses in patients with cancer. Results of several preclinical studies using mouse tumor models support this hypothesis [37-40]. In these studies, antibodies directed against PD-L1 or its receptor, PD-1, demonstrated antitumor activity.

Stimulating an antitumor immune response is a mechanism employed successfully by a number of approved cancer therapies. For example, aldesleukin (Proleukin®), a recombinant human interleukin (IL)-2, in renal cancer [41] and sipuleucel-T (Provenge®) in prostate cancer[42] both directly stimulate immune activation. Ipilimumab (Yervoy®) also stimulates the immune response, but does so by blocking the T-cell co-inhibitory molecule, CTLA-4, thereby removing an immunosuppressive signal. Blocking PD-L1 is an approach similar to CTLA-4 inhibition, but with some distinct differences. First, the expression of CTLA-4 and its ligands is restricted to the hematopoietic system and thus, the site of action for molecules targeting CTLA-4 is solely the peripheral lymphoid organs. In contrast, PD-L1 is

expressed on cells of the hematopoietic system and on a range of tumor types. Therefore, targeting PD-L1 should have additional effects within the tumor microenvironment. Second, CTLA-4 plays an early and critical role in controlling T-cell activation. This is reflected in the phenotype of CTLA-4 knockout mice, which die between 3 and 4 weeks of age due to lymphoproliferative disease and tissue destruction. In contrast, PD-L1 acts later in the process of T-cell activation [43] and is considered less critical to the control of initial T-cell activation. This is reflected in the phenotype of PD-L1 knockout mice, which are viable and have normal T-cell numbers and activation levels but show increased T-cell activation in response to antigen and increased susceptibility in certain autoimmune models [44, 45]. Similarly, mice lacking PD-1, a PD-L1 receptor, show strain-specific phenotypes that are milder than those seen in CTLA-4 knockouts [46, 47]. Therefore, inhibition of the PD-L1/PD-1 pathway might be expected to result in less toxicity relative to CTLA-4 inhibition. In support of these findings, recent Phase 1 clinical studies assessing the tolerability of agents targeting PD-1 have demonstrated a toxicity profile that is more favorable than that of CTLA-4 [48-51].

2.2 Durvalumab in Solid Tumors

2.2.1 Preclinical overview

Durvalumab Background

Durvalumab is being developed as a potential anticancer therapy for patients with advanced solid tumors. Durvalumab is a human monoclonal antibody (MAb) of the immunoglobulin G1 kappa (IgG1κ) subclass that inhibits binding of programmed cell death ligand 1 (PD-L1) (B7 homolog 1 [B7-H1], cluster of differentiation [CD]274) to programmed cell death 1 (PD-1; CD279) and CD80 (B7-1). Durvalumab is composed of 2 identical heavy chains and 2 identical light chains, with an overall molecular weight of approximately 149 kDa. Durvalumab contains a triple mutation in the constant domain of the immunoglobulin (Ig) G1 heavy chain that reduces binding to complement protein C1q and the fragment crystallizable gamma (Fcγ) receptors involved in triggering effector function. Preclinical data with durvalumab suggest that targeting PD-L1 with a biologic agent could be an effective antitumor therapy. Durvalumab is a human mAb that selectively binds human PD-L1 with high affinity and blocks its ability to bind to PD-1 and CD80. Blockade of PD-L1 with durvalumab relieved PD-L1-mediated suppression of human T-cell activation in vitro. In a xenograft model, durvalumab inhibited human tumor growth via a T-cell-dependent mechanism. Moreover, an anti-mouse PD-L1 antibody demonstrated improved survival in a syngeneic tumor model when given as monotherapy and resulted in complete tumor regression in > 50% of treated mice when given in combination with chemotherapy.

Durvalumab binds with high affinity and specificity to human PD-L1 and blocks its interaction with PD-1 and CD80. *In vitro* studies demonstrate that durvalumab antagonizes the inhibitory effect of PD-L1 on primary human T cells, resulting in their restored proliferation and release of interferon gamma (IFN-γ). Additionally, durvalumab demonstrated a lack of antibody-dependent cell-mediated cytotoxicity (ADCC) and complement-dependent cytotoxicity (CDC) in cell-based functional assays. In vivo studies show that durvalumab inhibits tumor growth in a xenograft model via a T lymphocyte (T-cell) dependent mechanism. Moreover, an anti-mouse PD-L1 antibody demonstrated improved survival in a syngeneic tumor model when given as monotherapy and resulted in complete tumor regression in > 50% of treated mice when given in combination with chemotherapy. Combination therapy (dual targeting of PD-L1 and cytotoxic T-lymphocyte-associated antigen 4 [CTLA-4]) resulted in tumor regression in a mouse model of colorectal cancer.

Cynomolgus monkeys were selected as the only relevant species for evaluation of the pharmacokinetics (PK)/pharmacodynamics and potential toxicity of durvalumab. Following intravenous (IV) administration, the PK of durvalumab in cynomolgus monkeys was nonlinear. Systemic clearance (CL) decreased and concentration half-life (t1/2) increased with increasing doses,

suggesting saturable target binding-mediated clearance of durvalumab. No apparent gender differences in PK profiles were observed for durvalumab.

In general, treatment of cynomolgus monkeys with durvalumab was not associated with any durvalumab -related adverse effects that were considered to be of relevance to humans. Adverse findings in the non-Good Laboratory Practice (GLP) PK/pharmacodynamics and dose range-finding study, and a GLP 4-week repeat-dose toxicity study were consistent with antidrug antibody (ADA)-associated morbidity and mortality in individual animals. The death of a single animal in the non-GLP, PK/pharmacodynamics, and dose range-finding study was consistent with an ADA-associated acute anaphylactic reaction. The spectrum of findings, especially the clinical signs and microscopic pathology, in a single animal in the GLP, 4-week, repeat-dose study was also consistent with ADA immune complex deposition, and ADA: durvalumab immune complexes were identified in a subsequent non-GLP, investigative immunohistochemistry study. Similar observations were reported in cynomolgus monkeys administered human mAbs unrelated to durvalumab. Given that immunogenicity of human mAbs in nonclinical species is generally not predictive of responses in humans, the ADA-associated morbidity and mortality were not considered for the determination of the no-observed-adverse-effect level (NOAEL) of durvalumab.

Finally, data from the pivotal 3-month GLP toxicity study with durvalumab in cynomolgus monkeys showed that subchronic dosing of durvalumab was not associated with any adverse effects. Therefore, the NOAEL of durvalumab in all the general toxicity studies was considered to be 100 mg/kg, the highest dose tested in these studies. In addition to the in vivo toxicology data, no unexpected membrane binding of durvalumab to human or cynomolgus monkey tissues was observed in GLP tissue cross-reactivity studies using normal human and cynomolgus monkey tissues.

2.2.2 Summary of clinical experience

As of the data cut-off (DCO) date of 12 July 2019, an estimated 8817 patients have received durvalumab in AstraZeneca or MedImmune-sponsored interventional studies in multiple tumour types, stages of disease and lines of therapy. Of these, 4067 patients received durvalumab monotherapy, 2423 patients received durvalumab in combination with tremelimumab, and 2327 patients received durvalumab in combination with an investigational and/or an approved product. An estimated 8343 patients have been randomised to the various treatment/comparator arms in sponsor-blinded studies. In addition, 2482 patients have participated in the durvalumab Early Access Programme (EAP; Study D4194C00002) for patients with locally advanced, unresectable NSCLC whose disease has not progressed following platinum-based chemoradiation therapy. The cumulative global post-marketing patient exposure to durvalumab (10 mg/kg) to 30 June 2019 has been estimated to be approximately 12385 patient-years.

2.2.3 Pharmacokinetics and Product Metabolism

Study CD-ON-durvalumab-1108: As of 09 Feb2015, PK data were available for 378 subjects in the dose-escalation and dose-expansion phases of Study CD ON-durvalumab-1108 following treatment with durvalumab 0.1 to 10 mg/kg every 2 weeks (Q2W) or 15 mg/kg every 3 weeks (Q3W). The maximum observed concentration (Cmax) increased in an approximately dose-proportional manner over the dose range of 0.1 to 15 mg/kg. The area under the concentration-time curve from 0 to 14 days (AUC0-14) increased in a greater than dose proportional manner over the dose range of 0.1 to 3 mg/kg and increased dose-proportionally at $\geq$ 3 mg/kg. These results suggest durvalumab exhibits nonlinear PK likely due to saturable target-mediated CL at doses < 3 mg/kg and approaches linearity at doses $\geq$ 3 mg/kg. Near complete target saturation (soluble programmed cell death ligand 1 [sPD L1] and membrane bound) is expected with durvalumab $\geq$ 3 mg/kg Q2W. Exposures after multiple doses showed accumulation consistent with PK parameters estimated from the first dose. In addition, PK simulations indicate that following durvalumab 10 mg/kg Q2W dosing, > 90% of subjects are expected to maintain PK exposure $\geq$ 40 µg/mL throughout the dosing interval.

As of 09 Feb2015, a total of 388 subjects provided samples for ADA analysis. Only 8 of 388 subjects (1 subject each in 0.1, 1, 3, and 15 mg/kg cohorts, and 4 subjects in 10 mg/kg cohort) were ADA positive with an impact on PK/pharmacodynamics in 1 subject in the 3 mg/kg cohort

2.2.4 Safety

Overall risks

Monoclonal antibodies directed against immune checkpoint proteins, such as programmed cell death ligand 1 (PD-L1) as well as those directed against programmed cell death-1 (PD-1) or cytotoxic T-lymphocyte antigen-4 (CTLA-4), aim to boost endogenous immune responses directed against tumor cells. By stimulating the immune system however, there is the potential for adverse effects on other tissues.
Most adverse drug reactions seen with the immune checkpoint inhibitor class of agents are thought to be due to the effects of inflammatory cells on specific tissues. These risks are generally events with a potential inflammatory mechanism and which may require more frequent monitoring and/or unique interventions such as immunosuppressants and/or endocrine replacement therapy. These risks include gastrointestinal AEs such as colitis and diarrhoea, pneumonitis /interstitial lung disease (ILD), renal AEs such as, nephritis and increases in creatinine, hepatic AEs such as hepatitis and liver enzyme elevations, skin events such as rash and dermatitis, endocrinopathies such as hypo- and hyperthyroidism, hypophysitis, adrenal insufficiency, diabetes mellitus type I and diabetes insipidus, and neurotoxicities such as myasthenia gravis and Guillain-Barre syndrome.

Durvalumab

Risks with durvalumab include diarrhea, colitis, pneumonitis /ILD, endocrinopathies (hypo- and hyperthyroidism, type I diabetes mellitus, diabetes insipidus, hypophysitis and adrenal insufficiency) hepatitis/hepatotoxicity/increases in transaminases, neurotoxicities, nephritis/increases in creatinine, pancreatitis, rash/pruritus/dermatitis, infusion-related reactions, anaphylaxis, hypersensitivity or allergic reactions, and immune complex disease.

Further information on these risks can be found in the current version of the durvalumab IB.

In monotherapy clinical studies, AEs (all grades) reported very commonly (≥10% of patients) are fatigue, nausea, decreased appetite, dyspnea, cough, diarrhea, vomiting, pyrexia, abdominal pain, peripheral edema, rash, pruritus, upper respiratory tract infection, and aspartate aminotransferase increased. Approximately 9.4% of patients experienced an AE that resulted in permanent discontinuation of durvalumab and approximately 6.5% of patients experienced an SAE that was considered to be related to durvalumab by the study investigator.

The majority of treatment-related AEs were manageable with dose delays, symptomatic treatment, and in the case of events suspected to have an immune basis, the use of established treatment guidelines for immune-mediated toxicity (see the Dosing Modification and Toxicity Management Guidelines in B.

A detailed summary of durvalumab monotherapy AE data can be found in the current version of the durvalumab IB.

AEs reported with durvalumab monotherapy in key clinical studies are described below.

Adverse Event Profile of durvalumab Monotherapy

Study CD-ON-durvalumab-1108: The safety profile of durvalumab monotherapy in the 694 subjects with advanced solid tumors treated at 10 mg/kg Q2W in Study CD-ON-durvalumab-1108 has been broadly consistent with that of the overall 1,279 subjects who have received durvalumab monotherapy (not including subjects treated with blinded investigational product) across the clinical development program. The majority of treatment-related AEs were manageable with dose delays, symptomatic treatment, and in the case of events suspected to have an immune basis, the use of established treatment guidelines for immune-mediated toxicity. As of 07 May2015, among the 694 subjects treated with durvalumab 10 mg/kg Q2W in Study CD-ON-durvalumab-1108, a total of 378 subjects (54.5%) experienced a treatment-related AE, with the most frequent (occurring in ≥ 5% of subjects) being fatigue (17.7%), nausea (8.6%), diarrhea (7.3%), decreased appetite (6.8%), pruritus (6.3%), rash (6.1%), and vomiting (5.0%). A majority of the treatment-related AEs were Grade 1 or Grade 2 in severity with ≥ Grade 3 events occurring in 65 subjects (9.4%). Treatment-related ≥ Grade 3 events reported in 3 or more subjects (≥ 0.4%) were fatigue (12 subjects, 1.7%); increased aspartate aminotransferase (AST; 7 subjects, 1.0%); increased gamma-glutamyltransferase (GGT; 6 subjects, 0.9%); increased alanine aminotransferase (ALT; 5 subjects, 0.7%); and colitis, vomiting, decreased appetite, and hyponatremia (3 subjects, 0.4% each). Six subjects had treatment-related Grade 4 AEs (upper gastrointestinal hemorrhage, increased AST, dyspnea, neutropenia, colitis, diarrhea, and pneumonitis) and 1 subject had a treatment-related Grade 5 event (pneumonia). Treatment-related serious adverse events (SAEs) that occurred in ≥ 2 subjects were colitis and pneumonitis (3 subjects each). A majority of the treatment-related SAEs were ≥ Grade 3 in severity and resolved with or without sequelae. AEs that resulted in permanent discontinuation of durvalumab were considered as treatment related in 18 subjects (2.6%), with colitis being the most frequent treatment-related AE resulting in discontinuation (3 subjects). A majority of the treatment-related AEs resulting in discontinuation of durvalumab were ≥ Grade 3 in severity and resolved with or without sequelae.

Study D4191C00003/ATLANTIC: The safety profile of durvalumab monotherapy in Study CD-ON-durvalumab-1108 is generally consistent with that of Study D4191C00003/ATLANTIC in subjects with locally advanced or metastatic non-small-cell lung cancer (NSCLC) treated with durvalumab 10 mg/kg Q2W. As of 05May2015, 264 of 303 subjects (87.1%) reported any AE in Study D4191C00003/ATLANTIC. Overall, events reported in ≥ 10% of subjects were dyspnea (18.8%), fatigue (17.8%), decreased appetite (17.5%), cough (14.2%), pyrexia (12.2%), asthenia (11.9%), and nausea (11.2%). Nearly two-thirds of the subjects experienced AEs that were Grade 1 or 2 in severity and manageable by general treatment guidelines as described in the current durvalumab study protocols. Grade 3 or higher AEs were reported in 107 of 303 subjects (35.3%). A total of 128 subjects (42.2%) reported AEs that were considered by the investigator as related to investigational product. Treatment-related AEs (all grades) reported in ≥ 2% of subjects were decreased appetite (6.6%); fatigue (5.9%); asthenia (5.0%); nausea (4.6%); pruritus (4.3%); diarrhea, hyperthyroidism, hypothyroidism, and pyrexia (3.3% each); rash (2.6%); weight decreased (2.3%); and vomiting (2.0%). Treatment-related Grade 3 AEs reported in ≥ 2 subjects were pneumonitis (3 subjects) and increased GGT (2 subjects). There was no treatment-related Grade 4 or 5 AEs. Ninety-four of 303 subjects (31.0%) reported any SAE. SAEs that occurred in ≥ 1.0% of subjects were dyspnea (6.6%); pleural effusion, general physical health deterioration (2.3% each); pneumonia (2.0%); hemoptysis, pulmonary embolism (1.3% each); and pneumonitis, respiratory failure, disease progression (1.0% each). Nine subjects had an SAE considered by the investigator as related to durvalumab. Each treatment-related SAE occurred in 1 subject each with the exception of pneumonitis, which occurred in 3 subjects. Fifteen of 303 subjects (5.0%) have died due to an AE (pneumonia [3 subjects]; general physical health deterioration, disease progression, hemoptysis, dyspnea [2 subjects each]; pulmonary sepsis, respiratory distress, cardiopulmonary arrest [verbatim term (VT)], hepatic failure, and sepsis [1 subject each]). None of these events was considered related to durvalumab. Twenty-three of 303 subjects

(7.6%) permanently discontinued durvalumab treatment due to AEs. Events that led to discontinuation of durvalumab in ≥ 2 subjects were dyspnea, general physical health deterioration, and pneumonia. Treatment-related AEs that led to discontinuation were increased ALT and increased hepatic enzyme, which occurred in 1 subject each.

2.2.5 Efficacy

Study CD-ON-durvalumab-1108: Overall, 456 of 694 subjects treated with durvalumab 10 mg/kg Q2W were evaluable for response (defined as having ≥ 24 weeks follow-up, measurable disease at baseline, and ≥ 1 follow-up scan, or discontinued due to disease progression or death without any follow-up scan). In PD-L1 unselected patients, the objective response rate (ORR), based on investigator assessment per Response Evaluation Criteria in Solid Tumors (RECIST) v1.1, ranged from 0% in uveal melanoma (n = 23) to 20.0% in bladder cancer (n = 15), and disease control rate at 24 weeks (DCR-24w) ranged from 4.2% in triple-negative breast cancer (TNBC; n = 24) to 39.1% in advanced cutaneous melanoma (n = 23). PD-L1 status was known for 383 of the 456 response evaluable subjects. Across the PD-L1-positive tumors, ORR was highest for bladder cancer, advanced cutaneous melanoma, hepatocellular carcinoma (HCC; n = 3 each, 33.3% each), NSCLC (n = 86, 26.7%), and squamous cell carcinoma of the head and neck (SCCHN; n = 22, 18.2%). In the PD-L1-positive subset, DCR-24w was highest in advanced cutaneous melanoma (n = 3, 66.7%), NSCLC (n = 86, 36.0%), HCC and bladder cancer (n = 3 each, 33.3% each), and SCCHN (n = 22, 18.2%).

Study D4190C00007: Of the 32 subjects with myelodysplastic syndrome (MDS) treated in Study D4190C00007, 21 subjects had at least 1 post-baseline disease assessment. Among these subjects, the best overall responses were marrow complete remission (mCR) in 4 subjects (19.0%); stable disease (SD) in 4 subjects (19.0%); and progressive disease (PD) in 5 subjects (23.8%). The remaining 8 subjects (38.1%) did not meet the criteria for complete remission (CR), mCR, partial remission (PR), SD, or PD at the date of assessment.

Study CD-ON-durvalumab-1161: Of the 65 subjects with metastatic or unresectable melanoma treated with the combination of durvalumab and BRAF inhibitor (BRAFi; dabrafenib)/MEK inhibitor (MEKi; trametinib), 63 subjects were evaluable for response. A total of 35 subjects (55.6%) had a best overall response of confirmed or unconfirmed PR. The disease control rate (DCR; CR + PR [regardless of confirmation] + SD ≥ 12 weeks) was 79.4%.

Dose rationale

This study will utilize an open-label design due to the different treatment administration schedules and treatment durations.

MEDI4736

A dose of MEDI4736 15 mg/kg (or 1.12g fixed dose) every 3 weeks (q3w) is supported by in-vitro data, non-clinical activity, clinical pharmacokinetics (PK)/pharmacodynamics, biomarkers, and activity data from Study 1108 in patients with advanced solid tumors (ongoing first-time-in-humans study) and from a Phase I trial performed in Japanese patients with solid tumor (NCT01938612).

PK/Pharmacodynamic data

Based on available PK/pharmacodynamic data from ongoing Study 1108 with doses ranging from 0.1 to 10 mg/kg every 2 weeks (q2w) or 15 mg/kg every 3 weeks (q3w), MEDI4736 exhibited non-linear (dose-dependent) PK consistent with target-mediated drug disposition. The PK approached linearity at ≥3 mg/kg q2w (trough concentration ~40 µg/mL), suggesting near complete target saturation (membrane-bound and soluble PD-L1 [sPD-L1]), and further shows that the MEDI4736 dosing frequency can be adapted to a particular regimen given the linearity seen at higher doses than 3 mg/kg. The expected half-life with doses ≥3 mg/kg q2w is approximately 21 days. A dose-dependent suppression in peripheral sPD-L1 was observed over the dose range studied, consistent with

engagement of MEDI4736 with PD-L1. Dose-related changes in a variety of peripheral biomarkers have been observed over the dose range of 0.1 to 15 mg/kg. A low level of immunogenicity has been observed. No patients have experienced immune-complex disease following exposure to MEDI4736. Of the 388 patients who received MEDI4736 monotherapy and for whom PK/ antidrug antibody (ADA) data were available as of 9 Feb July 2015, 5 were ADA positive, with an impact on PK/pharmacodynamics reported in 1 patient at 3 mg/kg. Population PK simulations indicate that following 15 mg/kgQ3W dose of MEDI4736, >90% patients are expected to maintain PK exposure yielding full target saturation throughout the dosing interval. In addition, PK simulation indicates similar overall exposure for both 10 mg/kg Q2W (dose selected for Phase 3 monotherapy studies) and 15 mg/kg Q3W. This is supported by available limited PK data following 15 mg/kg Q3W cohort in study 1108.

Clinical data

The initial safety data from a small subset of patients receiving the 15 mg/kg q3w on the 1108 study suggests that the nature and frequency of toxicities observed with 15 mg/kg q3w are consistent with the 10 mg q2w regimen. As of 07 May2015, 736 subjects were treated across all dose levels in Study 1108. Among the 694 subjects treated with MEDI4736 10 mg/kg Q2W, a total of 378 subjects (54.5%) experienced a treatment-related AE, with the most frequent (occurring in ≥ 5% of subjects) being fatigue (17.7%), nausea (8.6%), diarrhea (7.3%), decreased appetite (6.8%), pruritus (6.3%), rash (6.1%), and vomiting (5.0%). A majority of the treatment-related AEs were Grade 1 or Grade 2 in severity with ≥ Grade 3 events occurring in 65 subjects (9.4%). Treatment-related ≥ Grade 3 events reported in 3 or more subjects (≥ 0.4%) were fatigue (12 subjects, 1.7%); increased aspartate aminotransferase (AST; 7 subjects, 1.0%); increased gamma-glutamyltransferase (GGT; 6 subjects, 0.9%); increased alanine aminotransferase (ALT; 5 subjects, 0.7%); and colitis, vomiting, decreased appetite, and hyponatremia (3 subjects, 0.4% each). Six subjects had treatment-related Grade 4 AEs (upper gastrointestinal hemorrhage, increased AST, dyspnea, neutropenia, colitis, diarrhea, and pneumonitis) and 1 subject had a treatment-related Grade 5 event (pneumonia). AEs that resulted in permanent discontinuation of MEDI4736 were considered as treatment related in 18 subjects (2.6%), with colitis being the most frequent treatment-related AE resulting in discontinuation (3 subjects). A majority of the treatment-related AEs resulting in discontinuation of MEDI4736 were ≥ Grade 3 in severity and resolved with or without sequelae. As of this same data cutoff, 7 subjects were treated with MEDI4736 15 mg/kq3w. Among these 7 subjects, 6 experienced a treatment-related AE, with the most frequently reported events being fatigue and nausea (2 subjects each). At this dose level, 2 subjects experienced a treatment-related ≥ Grade 3 AE (peripheral motor neuropathy and fatigue in 1 subject each). One treatment-related AE resulted in permanent discontinuation of MEDI4736 (Grade 3 peripheral motor neuropathy). There were no dose-limiting toxicities (DLTs) or drug-related deaths observed in the 15 mg/kg q3w cohort. Overall, at both the 10 mg/kg q2w and 15 mg/kg q3w dose levels, AEs were manageable with dose delays, symptomatic treatment, and in the case of events suspected to have an immune basis, the use of established treatment guidelines for immune-mediated toxicity.

Data presented at the European Society for Medical Oncology (ESMO) meeting 2014 with a later cutoff of 21 August 2014 showed that MEDI4736 was well tolerated at all doses in the non-small-cell lung cancer (NSCLC) subset of patients enrolled into Study 1108, with drug-related Grade ≥3 AEs reported in 3% of patients and drug-related AEs leading to discontinuation reported in 1% of patients. No drug-related colitis or hyperglycemia of any grade, no Grade ≥3 pneumonitis reported, and no drug-related AEs leading to death were reported. At present, the data do not suggest that the safety profile of MEDI4736 will be different in the 15 mg/kg q3w dosing regimen when compared to 10 mg/kg q2w regimen.

Efficacy data on the Study 1108, presented at ESMO 2014 (cutoff date of 21 August 2014), showed a

disease control rate (DCR) at 12 weeks of 41% and objective response rate (ORR) of 16% among 162 evaluable patients, with activity observed in both squamous and non-squamous histologies. The ORR was higher (25%; 12 CR/PR; n=48) in patients with PD-L1 positive tumors, defined as those with ≥25% of tumor cells with membrane staining for PD-L1, compared to patients with PD-L1 negative tumors (10%; 7 CR/PR; n=74).

Fixed dosing rationale

A population PK model was developed for MEDI4736 using monotherapy data from Study 1108 (Phase I study; N=292; doses=0.1 to 10 mg/kg q2w or 15 mg/kg q3w; solid tumors). Population PK analysis indicated only a minor impact of body weight (WT) on the PK of MEDI4736 (coefficient of ≤0.5). The impact of body WT-based (10 mg/kg q2w) and fixed dosing (750 mg q2w) of MEDI4736 was evaluated by comparing predicted steady state PK concentrations (5th, median, and 95th percentiles) using the population PK model. A fixed dose of 750 mg was selected to approximate 10 mg/kg (based on a median body WT of approximately 75 kg). A total of 1000 patients were simulated using a body WT distribution of 40 to 120 kg. Simulation results demonstrated that body WT-based and fixed dosing regimens yield similar median steady state PK concentrations with slightly less overall between-patient variability with the fixed dosing regimen.

Similar findings have been reported by others (Narwal et al 2013, Ng et al 2006, Wang et al 2009, Zhang et al 2012). Wang and colleagues investigated 12 mAbs and found that fixed and body size-based dosing perform similarly, with fixed dosing being better for 7 of 12 antibodies (Wang et al 2009). In addition, they investigated 18 therapeutic proteins and peptides and showed that fixed dosing performed better for 12 of 18 in terms of reducing the between-patient variability in PK/pharmacodynamics parameters (Zhang et al 2012).

A fixed dosing approach is preferred by the prescribing community due to ease of use and reduced dosing errors. Given the expectation of similar PK exposure and variability, AstraZeneca considered it feasible to switch to fixed dosing regimens. Based on an average body WT of 75 kg, fixed doses of 1.12 g q3w (equivalent to 15 mg/kg q3w) of MEDI 4736 is included in the current study.

2.2.6 Targeting the PD1/PD-L1 pathway in NSCLC

The use of monoclonal antibodies that target immune checkpoints has unleashed intratumoral T cell mediated immune responses in a manner that has the potential to revolutionize lung cancer treatment. Two immune check point pathways that have been investigated and targeted for therapeutic purposes are the membrane receptors, cytotoxic T-lymphocyte-associated antigen 4 (CTLA-4), and programmed cell death protein 1 (PD1) and its ligand PD-L1. In contrast to anti-CTLA-4 antibodies, where there has been no significant single-agent activity in advanced NSCLC, blockade of the PD1/PD-L1 pathway resulted in significant single agent activity in heavily pretreated patients with NSCLC [11-18]. A trial of the anti- PD1 antibody, nivolumab, in 129 heavily pretreated patients with NSCLC was recently reported [14]. The objective response rate was 17% and was similar in squamous and non-squamous NSCLC. Impressively the duration of response was 17 months; an unprecedented duration of response compared to that seen after either chemotherapy or oncogene targeted therapy. These impressive response rates translated into a significant improvement in survival (1 year: 42% and 2 year: 24%). Although adverse events were generally mild, immune-related pneumonitis developed in 7% of patients and was associated with the death of three patients. Similar results were reported in a clinical trial that enrolled 495 patients with stage IIIB/IV NSCLC treated with the anti-PD1 antibody pembrolizumab [15]. The overall objective response rate was 19% and the median duration of response was 12.5 months. Interestingly the objective response rate and median duration of response were higher in 101 treatment naïve patients (24.8% and 23 months) compared to those in previously treated patients (18% and 10 months). Grade 3 or higher adverse events occurred in 9.5% of patients including grade 3 pneumonitis that developed in 9 patients (1.8%) and resulted in one of two treatment related deaths. Finally, Rizvi *et al* reported the results of a phase II trial of nivolumab monotherapy in 117

patients with refractory advanced squamous cell lung cancer [16]. The objective response rate was 14.5% and an additional 26% of patients had stable disease. Grade 3-4 events developed in 20 patients (17%) including pneumonitis that occurred in 4 patients and was associated with one treatment-related death. There are currently two anti-PD-L1 antibodies that have completed phase I expansion trials in patients with refractory advanced solid tumors: durvalumab and MPDL3280A [17, 18]. Both are fully humanized IgG1 antibodies engineered to bind with high affinity to PD-L1 (not PD-L2) and with an Fc modification that 2 prevents antibody-dependent cellular cytotoxicity, thus precluding lysis of activated T cells. MPDL3280A resulted in an objective response rate in 12 of 53 (23%) patients with refractory NSCLC and was durable in eleven [7]. Response was not significantly associated with tumor cell expression of PD-L1. No grade 3- 5 pneumonitis were reported. Rizvi et al reported on 198 patients with refractory heavily pretreated patients with NSCLC treated using durvalumab (the antibody selected for this proposal) in a global multicenter phase I expansion trial [18]. Objective responses by RECIST criteria were observed in 14% of patients (PD-L1+ve: 23%, PD-L1-ve: 10%). Responses were observed in patients with and without EGFR mutations, ALK re-arrangement or KRAS mutations. Responses occurred early and were durable in 76% of responders. Drug-related adverse events (AEs) of any grade occurred in 48% of patients [52]. Drug-related AEs ≥3 developed in 6% of patients. There was no drug-related colitis or hyperglycemia of any grade and importantly no grade ≥3 pneumonitis was reported. Because of the encouraging early results of anti-PDL1 antibodies in NSCLC and their favorable toxicity profiles, clinical trials have already been activated in early stage NSCLC. Two separate randomized phase III trials testing adjuvant therapy with durvalumab (NCT02273375/NCIC CTG BR.31) or MPDL3280A (Roche pharmaceutical) have been activated internationally including in the United States and Canada. Patients with completely resected NSCLC pathological stages 1B/II/IIIA will be randomized to either anti-PD-L1 antibody or placebo. Disease free survival is the primary endpoint in both trials

2.2.7 Radiation and tumor immunity

Radiation therapy is an essential component of the treatment of many solid tumors. Delivered alone, or in combination with chemotherapy, the aim of radiotherapy is to provide definitive and potentially curative tumor eradication for localized disease or palliation in the metastatic setting. In such instances the benefits of radiotherapy are generally attributed to a direct cytoreductive or cytocidal effect mediated by radiation induced DNA damage. However, emerging pre-clinical evidence suggests that radiotherapy, in addition to its direct cytocidal effects, can be a potent modulator of the local intratumoral and possibly systemic anti-tumor immune response, and thereby have specific anti-tumor effects beyond its direct effects on the cancer cells. Ionizing radiation may potentiate antitumor immunity by acting directly on tumor cells rendering them more immunogenic and by inducing adjuvant signals in the tumor microenvironment in a manner that favors an anti-tumor immune milieu. For example, radiation by acting directly on tumor cells can lead to “immunogenic cell death”; a phenomenon characterized not only by simple neo-antigen release from cancer cells but also by the release of various danger signals that favor dendritic cell maturation and activation, and the recruitment of tumor antigen specific CD8 T-cells [53-54]. These signals include the release of the nuclear protein high mobility group box 1, adenosine tri-phosphate as well as the translocation of calreticulin from the endoplasmic reticulum to the plasma membrane where it acts as an “eat me” signal for antigen presenting cells[55-57]. These cell death associated molecules bind their respective receptors on intra-tumoral immature dendritic cells leading to activation of the inflammasome with DC maturation, secretion of IL1ß and evolution of antigen specific CD8 T cells [52]. Radiation can enhance tumor antigen presentation by up-regulation in cancer cells of MHC class I molecules that are necessary for endogenous peptide presentation as well as by up-regulating the expression of tumor specific antigens such as cancer testis antigens [58-59]. Radiation can also render tumor cells more susceptible to T cell cytolytic activity by enhancing Fas gene expression[60]. Beyond its direct effect on cancer cells, ionizing radiation can modify the tumor microenvironment by triggering a cytokine/chemokine mediated

inflammatory response with the recruitment of immune/inflammatory cells including monocytes, macrophages, DCs as well as effector T cells [18]. Other radiation induced changes in the tumor microenvironment demonstrated in preclinical experiments include the up-regulation in endothelial cells of the adhesion molecules VCAM-1 and ICAM-1 that facilitate trafficking of immune cells into the tumor microenvironment[61].

Many of these radiation-induced effects on the cancer cell and its microenvironment have been demonstrated to occur in human cancer cell lines and preclinical models even with sub-lethal doses of radiation [62]. Despite its role in generating or potentiating an anti-tumor immune response, ionizing radiation can also contribute to an immuno-suppressive tumor microenvironment by the recruitment of myeloid derived suppressor cells, tumor associated macrophages and T-regs as well as by the inducing the secretion of the immunosuppressive cytokine, TGFß[63-65]. The net balance between the pro and anti-immunogenic effects of radiation therapy likely influences whether or not radiation results in an effective anti-tumor immune response. However, the currently available evidence supports the concept that radiation even at sub lethal doses can render cancer cells more immunogenic.

2.2.8 Radiation combined with immune checkpoint inhibition

Despite the success of radiation in controlling local disease, the majority of patients develop either regional or systemic relapse. Clearly the pro-immunogenic effects of radiation are insufficient to either initiate or sustain an effective and durable anti-tumor immune response. Combining radiotherapy with immune checkpoint blockade has been tested in several preclinical models. In an orthotopic mouse model of glioma, stereotactic radiation (10Gyx1) was given in combination with an anti-PD1 antibody[23]. Long-term cures were observed in some mice treated by combination therapy but in none treated by either modality alone. Post-mortem examination showed that the brains of cured animals contained increased cytotoxic T cells. Furthermore, when cured mice were re-challenged several months later by flank injections of isogenic tumor cells, they failed to establish flank tumors in contrast to tumor naïve mice consistent with a systemic tumor antigen-specific memory immune response. Using a breast cancer mouse model with a primary tumor on one flank and a secondary tumor on the other flank, Dewan et al tested various fractionation schemes of radiation (20Gyx1, 8Gyx3 and 6Gyx5) given with or without an anti-CTLA-4 antibody [20]. Radiation was delivered to the primary but not to the secondary site. Treatment with the antibody alone had no discernible effect on tumor growth. All fractionation schemes of radiation resulted in tumor growth delay in the irradiated primary tumor. Combination therapy resulted in enhanced tumor response at the primary site in all radiation treated animals. A tumor response at the secondary non-irradiated site (an abscopal effect) occurred only in mice treated with fractionated radiotherapy and was abrogated by T cell depletion[21]. Recently, Dovedi et al reported that in animal models of triple negative breast cancer, melanoma and colon cancer treatment with 5 daily fractions of radiation (2Gyx5) and anti-PDL1 antibody was curative in 66% of mice[22]. Long-term survival was associated with efficacious CD8+ T–cell responses and the induction of tumor antigen-specific memory immune response protecting against isogenic tumor re-challenge. The authors also found that treatment sequencing was critical for a favorable outcome with long-term survival associated with concurrent rather than sequential administration of combined therapy. The dose and fractionation of radiation necessary for optimal immune potentiation as well the exact sequencing of immune checkpoint inhibition have yet to be fully investigated. However, the preclinical studies by Dewan and Dovedi highlight the importance of using multiple fractions of radiation and concurrent administration of immune checkpoint blockade for generating efficacious CD8 T-cell responses necessary for tumor control and generation of antigen-specific immune memory to protect against re-challenge. This synergism between radiation and immune checkpoint inhibition is currently being tested in controlled clinical trials in some advanced solid tumors. Interestingly there are at least three well documented case reports of an abscopal effect observed in two patients with metastatic melanoma and two patients with metastatic lung cancer all of whom were treated by targeted hypofractionated radiation and ipilumumab [66-68]. Both patients with NSCLC had refractory disease and

required localized multiple fractions of radiation to a metastatic site along with anti-CTLA-4. A complete clinical response was documented in both patients including in metastatic sites outside the radiation field[29]. In the two patients with melanoma, tumor regression was associated with evidence of epitope spreading, strongly suggesting an immune contribution to the clinical outcome [66, 67].

3.0 PATIENT SELECTION

3.1 Inclusion Criteria

1. Patient has histologically or cytologically proven clinical stages I (tumors > 2 cm), II, and IIIA NSCLC and is considered eligible for surgical resection with curative intent. Patients with 2 primary non-small cell lung cancers are allowed.
2. Measureable disease, as defined by RECIST v1.1.
3. Written informed consent and HIPAA obtained from the subject prior to performing any protocol-related procedures.
4. Age $\geq$ 18 years at time of study entry
5. Eastern Cooperative Oncology Group (ECOG) performance status of 0 or 1.
6. Adequate normal organ and marrow function as defined below:
   - Haemoglobin ≥ 9.0 g/dL
   - Absolute neutrophil count (ANC) ≥ 1.5 x 10^9/L (> 1500 per mm^3)
   - Platelet count ≥ 100 x 10^9/L (>100,000 per mm^3)
   - Serum bilirubin ≤ 1.5 x institutional upper limit of normal (ULN). This will not apply to subjects with confirmed Gilbert's syndrome (persistent or recurrent hyperbilirubinemia that is predominantly unconjugated in the absence of hemolysis or hepatic pathology), who will be allowed only in consultation with their physician.
   - AST (SGOT)/ALT (SGPT), and alkaline phosphatase ≤ 2.5 x institutional upper limit of normal (ULN).
   - Serum creatinine CL>30 mL/min by the Cockcroft-Gault formula (Cockcroft and Gault 1976) or by 24-hour urine collection for determination of creatinine clearance:

     Males:

     $$\text{Creatinine CL (mL/min)} = \frac{\text{Weight (kg)} \times (140 - \text{Age})}{72 \times \text{serum creatinine (mg/dL)}}$$

     Females:

     $$\text{Creatinine CL (mL/min)} = \frac{\text{Weight (kg)} \times (140 - \text{Age})}{72 \times \text{serum creatinine (mg/dL)}} \times 0.85$$

7. Evidence of post-menopausal status or negative urinary or serum pregnancy test for female pre-menopausal patients. Women will be considered post-menopausal if they have been amenorrheic for 12 months without an alternative medical cause. The following age-specific requirements apply:

   Women <50 years of age would be considered post-menopausal if they have been amenorrheic for 12 months or more following cessation of exogenous hormonal treatments and if they have

luteinizing hormone and follicle-stimulating hormone levels in the post-menopausal range for the institution or underwent surgical sterilization (bilateral oophorectomy or hysterectomy).

Women ≥50 years of age would be considered post-menopausal if they have been amenorrheic for 12 months or more following cessation of all exogenous hormonal treatments, had radiation-induced menopause with last menses >1 year ago, had chemotherapy-induced menopause with last menses >1 year ago, or underwent surgical sterilization (bilateral oophorectomy, bilateral salpingectomy or hysterectomy).

8. Subject is willing and able to comply with the protocol for the duration of the study including undergoing treatment and scheduled visits and examinations including follow up.
9. No prior therapy for their lung cancer.

3.2 Exclusion Criteria

Subjects should not enter the study if any of the following exclusion criteria are fulfilled:

1. Participation in another clinical study with an investigational product during the last 3 weeks.
2. History of another primary malignancy except for:
    - Malignancy treated with curative intent and with no known active disease ≥3 years before the first dose of study drug and of low potential risk for recurrence.
    - Adequately treated non-melanoma skin cancer or lentigo maligna without evidence of disease.
    - Adequately treated carcinoma in situ without evidence of disease eg, cervical cancer in situ, in-situ urinary bladder cancer , treated localized prostate cancer and ductal carcinoma-in situ.
    - Indolent hematological malignancies
3. Current or prior use of immunosuppressive medication within 14 days before the first dose of durvalumab, with the exceptions of intranasal,inhaled, topical steroids, or local steroid injections (e.g., intra articular injection), corticosteroids or systemic corticosteroids at physiological doses, which are not to exceed 10 mg/day of prednisone, or an equivalent corticosteroid, and steroids as premedication for hypersensitivity reactions (e.g., CT scan premedication).
4. Any unresolved toxicity (CTCAE grade 2) from therapy for a prior malignancy with the exception of alopecia, vitiligo, and the laboratory values defined in the inclusion criteria.
    - Patients with Grade ≥2 neuropathy will be evaluated on a case-by-case basis after consultation with the Study Physician.
    - Subjects with irreversible toxicity that is not reasonably expected to be exacerbated by the investigational product may be included (e.g., hearing loss, peripherally neuropathy).
5. Any prior Grade ≥3 immune-related adverse event (irAE) while receiving any previous immunotherapy agent, or any unresolved irAE >Grade 1.
6. Active or prior documented autoimmune or inflammatory disorders (including inflammatory bowel disease [e.g., colitis or Crohn's disease systemic lupus erythematosus, Sarcoidosis syndrome, or Wegener syndrome [granulomatosis with polyangiitis, Graves' disease, rheumatoid arthritis, hypophysitis, uveitis, etc]). No active diverticulitis within the previous 3 months. The following are exceptions to this criterion:
    - Patients with vitiligo or alopecia
    - Patients with hypothyroidism (e.g., following Hashimoto syndrome) stable on hormone replacement
    - Any chronic skin condition that does not require systemic therapy
    - Patients without active disease in the last 5 years may be included but only after consultation with the study physician

- Patients with celiac disease controlled by diet alone

7. Active or prior documented inflammatory bowel disease (e.g., Crohn's disease, ulcerative colitis).
8. History of active primary immunodeficiency.
9. History of allogeneic organ transplant.
10. History of hypersensitivity to durvalumab or any excipient.
11. Uncontrolled intercurrent illness including, but not limited to, ongoing or active infection, symptomatic congestive heart failure, uncontrolled hypertension, unstable angina pectoris, cardiac arrhythmia, serious chronic gastrointestinal conditions associated with diarrhea, or psychiatric illness/social situations that would limit compliance with study requirement, substantially increase risk of incurring AEs or compromise the ability of the patient to give written informed consent.
12. Active infection including tuberculosis (clinical evaluation that includes clinical history, physical examination and radiographic findings, and TB testing in line with local practice), hepatitis B (known positive HBV surface antigen (HBsAg) result), hepatitis C, or human immunodeficiency virus (positive HIV 1/2 antibodies). Patients with a past or resolved HBV infection (defined as the presence of hepatitis B core antibody [anti-HBc] and absence of HBsAg) are eligible. Patients positive for hepatitis C (HCV) antibody are eligible only if polymerase chain reaction is negative for HCV RNA.
13. History of leptomeningeal carcinomatosis.
14. Receipt of live attenuated vaccination within 30 days prior to study entry or within 30 days of receiving durvalumab.
15. Female patients who are pregnant or breastfeeding or male or female patients of reproductive potential who are not willing to employ effective birth control from screening to 90 days after the last dose of durvalumab monotherapy.
16. Any condition that, in the opinion of the investigator, would interfere with evaluation of study treatment or interpretation of patient safety or study results
17. Subjects with uncontrolled seizures.
18. History of interstitial lung disease, idiopathic pulmonary fibrosis, pneumonitis (including drug induced), or evidence of active pneumonitis on screening chest CT scan.
19. Major surgical procedure within 28 days prior to Cycle 1, Day 1 or anticipation of need for a major surgical procedure during the course of the study.
20. Receipt of the last dose of therapy (chemotherapy, immunotherapy, endocrine therapy, targeted therapy, biologic therapy, tumor embolization, monoclonal antibodies, or other investigational agent) for an accepted other malignancy as defined in Section 3.3.2 within 30 days prior to the first dose of study drug for lung cancer.
21. Prior randomisation or treatment in a previous durvalumab clinical study. regardless of treatment arm assignment.

4.0 REGISTRATION AND RANDOMIZATION PROCEDURES

4.1 Registration

Eligible subjects will be identified by the Principal Investigator's and co-investigators' from the Thoracic or Medical Oncology clinics at NYP-Weill Cornell or collaborating institutions. Individuals will be approached and informed of the rationale for the study, investigational nature of the protocol and study drug and the voluntary nature of participation, potential risks and benefits, alternatives to participation, and study procedures. Individuals will have the opportunity to read the written informed consent document with the understanding that should they decide not to participate, they will still be able to receive any available therapy off-trial. Potential subjects will also have the opportunity to obtain the advice of their treating physician. Investigators or delegates under their direct supervision

will verify the subject's understanding of the investigational and voluntary nature of the study, the potential risks and benefits, study procedures, and alternatives prior to signing of the written informed consent. Those who wish to participate in the trial will be required to sign an informed consent form after all their questions are answered. Subjects will be registered to the trial once they have met the eligibility criteria. Documentation of the consent process and that written consent was conducted must be documented in the subject's medical record.

4.2 Randomization

Blocked randomization will be performed for the secondary aim. A series of randomized blocks of 4 will be generated with a 1:1 allocation ratio. This will provide assurance that after four patients are enrolled in the study, there will be two patients assigned to the antibody + radiation arm and two patients assigned to the antibody + no radiation arm. This procedure will allow for a similar number of antibody + radiation patients (Arm 2) and antibody + no radiation patients (Arm 1).

5.0 STUDY DESIGN

This is a randomized open label phase II trial of preoperative anti-PD-L1 antibody durvalumab with or without concurrent non-ablative radiation followed by surgical resection and postoperative maintenance durvalumab every 4 weeks for 12 cycles.

5.1 Treatment Plan

Patients with biopsy proven NSCLC clinical stages I (tumors > 2 cm), II, and IIIA will be randomly assigned to 2 preoperative cycles of the anti-PD-L-1 antibody durvalumab followed by surgical resection *or* non-ablative stereotactic radiotherapy given concurrently with the first cycle of the anti-PD-L1 antibody followed by an additional cycle of the antibody alone followed by surgical resection. In both arms of the trial, patients will receive adjuvant durvalumab every 4 weeks for a total of 12 cycles. Patients randomized to the antibody alone arm will receive two cycles of durvalumab at a dose of 1.12 g every three weeks. Patients in the radio-immunotherapy arm will also be treated with two cycles of durvalumab (1.12 g q 3 weeks) plus radiotherapy delivered in 3 daily fraction of 8Gy starting concurrently with the first cycle of durvalumab (see trial schema). This delivered non-ablative radiation dose of 24 Gy is equivalent to a biologically effective dose (BED) of 43.2Gy, which is significantly lower than the standard therapeutic dose for ablation of T1-T2 NSCLC (BED>100Gy). The objective is to induce immunogenic cell death, enhance antigen presentation and increase cytotoxic T lymphocyte activity without tumor ablation. This non-ablative dose of radiation is selected based on the results of studies by one of our co-investigators showing that the 8 Gyx3 regimen resulted in significant immune modulation in preclinical models (24). A research core biopsy for the collection of tumor tissue is suggested but not mandatory. If a tissue biopsy has already been obtained for diagnostic purposes, a core biopsy may be performed for research purposes only to obtain tumor tissue. If a tissue biopsy has not been performed yet for diagnostic purposes, a core biopsy may be performed for diagnosis and tissue will be obtained for research purposes. Tissue from the preoperative core samples and from the resected surgical specimens will be stored for future correlative studies. Although PDL-1 expression is not required for eligibility, preoperative and post-operative tissue will be stored for future determination of PDL-1 expression. All patients will proceed to surgical resection 2-6 weeks after the second cycle of durvalumab regardless of local response assessment and provided that repeat imaging shows no evidence of systemic disease. Postoperative durvalumab will commence 4-12 weeks postoperatively at a fixed dose of 1.5 g every 4 weeks for 12 cycles. Patients receiving adjuvant chemotherapy may commence adjuvant monthly anti-PD-L1 after completing chemotherapy.

5.2 Limiting normal tissue toxicities with non-ablative SBRT

The thoracic organs that are at risk for radiation injury (i.e., organs at risk [OARs] from non-ablative SBRT doses and are expected to be determinants of toxicity are lung, central airway/bronchi,

esophagus, heart, great vessels, spinal cord, and brachial plexus). There is a large body of knowledge on the RT tolerance of those organs to conventionally fractionated RT (1.8-2 Gy per fraction), describing the RT tolerance in terms of volume of the organ irradiated to a certain dose (e.g., V20, volume irradiated to 20 Gy or more) or as mean RT dose to the entire organ. Recently the Task Group 101 (TG-101) of the American Association of Physicists in Medicine (AAPM) has prepared a report to outline the best practice guidelines for SBRT, including protocols, equipment, resources, and quality assurance procedures.[6] The TG-101 guidelines by the AAPM will be respected. Because the nidus of irradiated tumor and peri-tumoral lung will be removed at the time of surgery, we expect the probability of radiation pneumonitis to be minimal.

- Immobilization: The SBRT frame serves to both immobilize the patient physically and provide an initial approximate target localization, which is refined by in-room image guided techniques. Abdominal compression can be used to minimize respiratory motion, but is not required.
- Simulation imaging: Three-dimensional data sets assembled from 4DCT for visualization and dose calculation for SBRT. CT will be the primary imaging modality for SBRT and will form the basis for treatment calculations. Simulation will take place with the patient in the treatment position. A typical scan should extend at least 5-10 cm (15cm for non-coplanar beams) superior and inferior beyond he treatment field. Along with the target, all OARs should be included and covered by the selected scan length so they can be considered by the treatment planning software and evaluated with dose-volume histograms. For SBRT applications, tomographic slice thickness of 1-3 mm will be used.
- Data acquisition for mobile lung tumors and respiratory motion management: Techniques to image moving targets include slow CT, breath-hold techniques, gated approaches, 4DCT used in conjunction with maximum-intensity projection, minimum-intensity projection, and respiration-correlated PET-CT.
- Treatment planning: Planning will make use of the ICRU 50 and 62 definitions for gross tumor volume (GTV), clinical tumor volume (CTV), planning target volume (PTV) and OAR. With SBRT the GTV and CTV are considered identical. The PTV addresses all the possible geometrical variations by adding a variable margin for setup uncertainties, machine tolerances, and intratreatment variations to the CTV. By using the 4DCT to treatment planning the CTV to PTV expansion will be 0.5 cm in all directions.
- Dose constraints: Dose constraints for OARs published by the TG-101 AAPM report will be respected.
- Image-guided localization: Volumetric image guidance will allow for the precise localization of bone and soft tissue targets. This will be achieved with MV of kV cone beam scanning.

5.3 Duration of follow-up

Post-operatively, patients will be followed monthly from the date of the last infusion dose until month 4, at month 6, and subsequently every 6 months for 2 years. Thereafter, patients will be followed yearly till year 5. Patients removed from the study for unacceptable adverse events will be followed until resolution or stabilization of the adverse event.

Follow-up imaging will be performed by CT scan every 6 months for 2 years then yearly thereafter till year 5. Recurrent disease should be pathologically confirmed whenever possible.

5.4 Withdrawal of Subjects from Study Treatment and/or Study

5.4.1 Permanent discontinuation of Durvalumab

An individual subject will not receive any further investigational product if any of the following occur in the subject in question:

1. Withdrawal of consent or lost to follow-up
2. Adverse event that, in the opinion of the investigator or the sponsor, contraindicates further dosing
3. Subject is determined to have met one or more of the exclusion criteria for study participation at study entry and continuing investigational therapy might constitute a safety risk
4. Pregnancy or intent to become pregnant
5. Any AE that meets criteria for discontinuation as defined in Section 6.0
6. Adverse event related to durvalumab that is Grade ≥ 3, with the exception of toxicities that do not meet criteria for discontinuation as defined in Section 6.0
7. Grade ≥ 3 infusion reaction
8. Subject noncompliance that, in the opinion of the investigator or sponsor, warrants withdrawal; eg, refusal to adhere to scheduled visits

Initiation of alternative anticancer therapy including another investigational agent
Subjects who are permanently discontinued from further receipt of investigational product, regardless of the reason (withdrawal of consent, due to an AE, other), will be identified as having permanently discontinued treatment. Subjects who are permanently discontinued from receiving investigational product will be followed for safety, including the collection of any protocol-specified blood specimens, unless consent is withdrawn or the subject is lost to follow-up or enrolled in another clinical study. All subjects will be followed for survival.

5.4.2 Withdrawal of consent

If consent is withdrawn, the subject will not receive any further investigational product or further study observation.
If a patient withdraws consent, they will be specifically asked if they are withdrawing consent to:

- all further participation in the study including any further follow up (e.g., survival contact telephone calls)
- withdrawal of consent to the use of their study generated data
- withdrawal to the use of any samples

5.5 STUDY PROCEDURES

The Schedules of Assessments during the screening and treatment period is provided below.

Table 1. Schedule of study assessments: Screening and Treatment Period

**Visit** (Assessments to be performed at the times stipulated in the table and as clinically required in the management of the subject.)	**Screening**	**All assessments to be performed pre-infusion unless stated otherwise**				
		**Baseline**	**Every 3 weeks**	**Pre-operative**	**Surgical resection** [m]	**Postoperatively every 4 weeks for 12 cycles**
**Day**	**-28 to -1**	**1**	**22**	**36-49**	**36-69**	**Day 1 of the week**
**Week**	**-4 to -1**	**1**	**4**	**6-7**	**6-10**	**11, 15, 19, 23, 27, 31, 35, 39, 43, 47, 51, 55**
			**(±3 days)**			
Written informed consent/assignment of subject identification number	X					
Preliminary eligibility fulfilment (investigator's opinion)	X					
Demography and history of tobacco and alcohol use	X					
Previous treatments for NSCLC	X					
Core Biopsy[a]	X					
Formal verification of eligibility criteria	X					
Medical and surgical history	X					
Hepatitis A antibody, hepatitis B surface antigen, hepatitis C antibody; HIV antibody	X					
Urine hCG or serum βhCG[b]	X	X	As clinically indicated			

Table 1. Schedule of study assessments: Screening and Treatment Period

**Visit** (Assessments to be performed at the times stipulated in the table and as clinically required in the management of the subject.)	**Screening**	**All assessments to be performed pre-infusion unless stated otherwise**				
		**Baseline**	**Every 3 weeks**	**Pre-operative**	**Surgical resection** [m]	**Postoperatively every 4 weeks for 12 cycles**
**Day**	**-28 to -1**	**1**	**22**	**36-49**	**36-69**	**Day 1 of the week**
**Week**	**-4 to -1**	**1**	**4**	**6-7**	**6-10**	**11, 15, 19, 23, 27, 31, 35, 39, 43, 47, 51, 55**
			**(±3 days)**			
ARM 1 Durvalumab administration (monotherapy)		X	X			X [k] (monotherapy)
ARM 2 Durvalumab administration plus SBRT 8 Gy		X[l]				X [k] (monotherapy)
Physical examination[c]	X	X	X	X		X
Vital signs (pre- during and post-infusion vital signs assessments)[d]	X	X	X	X		X
Weight	X	X	X	X		X
Electrocardiogram[e]	X	X				
Adverse event/serious adverse event assessment	X	X	All visits			
Concomitant medications	X	X	All visits			
ECOG performance status	X	X	X	X		X
Serum Chemistry (complete clin chem. panel including Liver enzymes)[f]	X	X	X	X		X

Table 1. Schedule of study assessments: Screening and Treatment Period

**Visit** (Assessments to be performed at the times stipulated in the table and as clinically required in the management of the subject.)	**Screening**	**All assessments to be performed pre-infusion unless stated otherwise**				
		**Baseline**	**Every 3 weeks**	**Pre-operative**	**Surgical resection** [m]	**Postoperatively every 4 weeks for 12 cycles**
**Day**	**-28 to -1**	**1**	**22**	**36-49**	**36-69**	**Day 1 of the week**
**Week**	**-4 to -1**	**1**	**4**	**6-7**	**6-10**	**11, 15, 19, 23, 27, 31, 35, 39, 43, 47, 51, 55**
			**(±3 days)**			
Gamma glutamyltransferase	X	X	As clinically indicted			X
Thyroid function tests (TSH and fT3 and fT4)[g]	X	X	X	X		X
Hematology[f]	X	X	X	X		X
Urinalysis[h]	X	X	X			X
Coagulation parameters[i]	X	As clinically indicted				X
Tumor assessment (CT/PET scan) [n]	X			X		
Tumor assessment (Chest CT scan non-contrast) [j]						X

[a] Tissue from core biopsies and resected specimens will be stored for future correlative studies . A research core biopsy for collection of tumor tissue is suggested but not mandatory.
[b] Pre-menopausal female subjects of childbearing potential only.
[c] Full physical examination at baseline; targeted physical examination at other timepoints.
[d] Vital signs are temperature, blood pressure, pulse rate, and respiratory rate. Subjects will have their blood pressure and pulse measured before, during and after the infusion at the following times (based on a 60-minute infusion):

- At the beginning of the infusion (at 0 minutes)
- At 30 minutes during the infusion (±5 minutes)
- At the end of the infusion (at 60 minutes ±5 minutes)
- In the 1 hour observation period post-infusion: 30 and 60 minutes after the infusion (ie, 90 and 120 minutes from the start of the infusion) (±5 minutes) – for the first infusion only and then for subsequent infusions as clinically indicated
  If the infusion takes longer than 60 minutes then blood pressure and pulse measurements should be collected every 30 minutes (±5 minutes) and as described above or more frequently if clinically indicated.

[e] At screening and at Day 1-baseline, and as clinically indicated. Baseline and abnormal ECG at any time in triplicate, at screening in single. ECGs should be taken within an hour prior to the start of the infusion at Baseline C1D1.
[f] If screening laboratory assessments are performed within 7 days prior to Day 1 they do not need to be repeated at Day 1. Results for safety bloods must be available and reviewed before commencing an infusion. Gamma glutamyltransferase (GGT) tested at Screening, Day 1 and as clinically indicated.
[g] Free T3 and free T4 will only be measured if TSH is abnormal. They should also be measured if there is clinical suspicion of an adverse event related to the endocrine system.
[h] Urinalysis performed at Screening, Day 1, Day 22, every 4 weeks during adjuvant durvalumab treatment, and as clinically indicated.
[i] Coagulation tests: prothrombin time, APTT and INR – only performed at Screening and as clinically indicated.
[j] Post-operative CT scans are performed every 6 months for 2 years then yearly until year 5.
[k] Subjects in Arm 1 and Arm 2 will receive adjuvant treatment with durvalumab monotherapy at 1.5 g every 4 weeks for 12 cycles starting 4-12 weeks postoperatively, following standard of care postoperative therapy, as required. Refer to Section 8.1.3 for subjects weighing ≤ 30 kg.
[l] SBRT 8 Gy will be given on days 1, 2, and 3 only. On day 1, SBRT will be given prior to the durvalumab administration.
[m] Surgical resection will take place 2 -6 weeks after the last dose of durvalumab. Tumor, adjacent normal lung, and lymph node specimens will be obtained from the surgical resection specimen, snap frozen.
[n] Standard of care CT/PET scans performed prior to obtaining informed consent and within 42 days prior to C1D1 may be used for screening.

Screening laboratory procedures performed within 7 days of Cycle 1 Day 1 (C1D1) do not need to be repeated on C1D1.

A 12-lead ECG will be recorded for all subjects on study days noted in Section 5.5, Schedule of Study Assessments. ECGs are required during Screening, at Baseline prior to starting study treatment on Cycle 1 Day 1 (C1D1), and at any other time point when clinically indicated. ECGs recorded during the screening period will be single tracing and ECGs recorded at Baseline prior to C1D1 will be obtained in triplicate (with 2-5 minute lag time between each). All 12-lead ECGs should be recorded while the subject is in the supine position. The same method of assessment should be used throughout the study. Twelve-lead ECGs will be obtained after the subject has been resting in a supine position for at least 5 minutes in each case. At Screening, a single ECG will be obtained on which QTcF must be <470 ms. In case of clinically significant ECG abnormalities, including a QTcF value >470 ms, 2 additional 12-lead ECGs should be obtained over a brief period (e.g., 30 minutes) to confirm the finding.

Vital signs (temperature, blood pressure, pulse rate, and respiratory rate) will be measured on study days noted in the Schedule of Assessments. On durvalumab treatment days, vital signs will be measured within an hour prior to start of durvalumab administration, at 30 minutes during the infusion (± 5 minutes), at the end of infusion (+ 5 minutes), and at 30 minutes (± 5 minutes) and 60 minutes (± 5 minutes) post-infusion. If the infusion takes longer than 60 minutes, then blood pressure and pulse measurements should follow the principles described here, or more frequently if clinically indicated. For subsequent doses the 1-hour observation period will not be required unless a subject experiences an infusion-related reaction.

Table 2. Schedule of study procedures: follow-up for subjects who have completed or discontinued Durvalumab treatment

Evaluation	Time Since Last Dose of Durvalumab					
	Day (±3)	Months (±1 week)				Every 6 Months for 2 years then yearly till year 5 (±2 weeks)
	30	2	3	4	6	
Physical examination[a]	X					
Vital signs (temperature, respiratory rate, blood pressure, pulse)	X					
Weight	X					
Urine hCG or serum βhCG	X					
AE/SAE assessment	X	X	X			
Concomitant medications	X	X	X			
ECOG performance status	X	X	X		X	X
Adjuvant anti-cancer therapy, if required, as standard of care	X	X	X	X	X	X
Phone contact with subjects				X		
Hematology	X	X	X			
Serum chemistry	X	X	X			
Thyroid function tests (TSH, and fT3 and fT4)[b]	X					
Tumour assessment (non-contrast Chest CT scan)	Tumour assessments should be performed every 6 months for 2 years then yearly until year 5.					

[a] Full physical exam
[b] Free T3 and free T4 will only be measured if TSH is abnormal. They should also be measured if there is clinical suspicion of an adverse event related to the endocrine system.
The following clinical laboratory tests will be performed (see Schedule of study assessments and Schedule of procedures: follow-up) for the timepoints of each test):

- Coagulation parameters: Activated partial thromboplastin time and International normalised ratio to be assessed at baseline and as clinically indicated
- Pregnancy test (female subjects of childbearing potential only)
  - Urine human chorionic gonadotropin
  - Serum beta-human chorionic gonadotropin (at screening only)
- Thyroid Stimulating Hormone
  - free T3 and free T4 only if TSH is abnormal or if there is a clinical suspicion of an AE related to the endocrine system
- Other laboratory tests
  - Hepatitis A antibody, hepatitis B surface antigen, hepatitis C antibody
  - HIV antibody

Hematology Laboratory Tests

Basophils	Mean corpuscular volume
Eosinophils	Monocytes
Hematocrit	Neutrophils
Hemoglobin	Platelet count
Lymphocytes	Red blood cell count
Mean corpuscular hemoglobin	Total white cell count
Mean corpuscular hemoglobin concentration	

Clinical chemistry(serum or plasma) Laboratory Tests

Albumin	Glucose
Alkaline phosphatase (ALP)	Lactate dehydrogenase
Alanine aminotransferase (ALT)	Lipase
Amylase	Magnesium
Aspartate aminotransferase (AST)	Potassium
Bicarbonate	Sodium
Calcium	Total bilirubin[a]
Chloride	Total protein
Creatinine	Urea or blood urea nitrogen, depending on local practice
Gamma glutamyltransferase[b] (GGT)	Uric acid

[a] If Total bilirubin is ≥2xULN (and no evidence of Gilbert's syndrome) then fractionate into direct and indirect bilirubin
[b] At baseline and as clinically indicated

Urinalysis Tests

Bilirubin	pH
Blood	Protein
Glucose	Specific gravity
Ketones	Colour and appearance

[a] Microscopy should be used as appropriate to investigate white blood cells and use the high power field for red blood cells

Assessments for subjects who have completed durvalumab treatment or have discontinued durvalumab due to toxicity in the absence of confirmed progressive disease are provided in confirmed progressive disease are provided in Table 2 above.

6.0 DOSE DELAYS/DOSE MODIFICATIONS

6.1 Dose Modification and Toxicity Management

Guidelines for the management of immune-mediated reactions, infusion-related reactions, and non-immune-mediated reactions for durvalumab are in the Dosing Modification and Toxicity Management Guidelines in Appendix B. Patients should be thoroughly evaluated and appropriate efforts should be made to rule out neoplastic, infectious, metabolic, toxin, or other etiologic causes of the imAE. Serologic, immunologic, and histologic (biopsy) data, as appropriate, should be used to support an imAE diagnosis. In the absence of a clear alternative etiology, events should be considered potentially immune related. In addition, there are certain circumstances in which durvalumab should be permanently discontinued (see section 5.4.1 and the Dosing Modification and Toxicity Management Guidelines in Appendix B).

Following the first dose of durvalumab, subsequent administration of durvalumab can be modified based on toxicities observed as described in the Dosing Modification and Toxicity Management Guidelines in APPENDIX B. Dose reductions are not permitted. Based on the mechanism of action of durvalumab leading to T-cell activation and proliferation, there is the possibility of observing immune related Adverse Events (irAEs) during the conduct of this study. Potential irAEs include immune mediated enterocolitis, dermatitis, hepatitis, and endocrinopathies. Subjects should be monitored for signs and symptoms of irAEs. In the absence of an alternate etiology (e.g., infection or PD) signs or symptoms of enterocolitis, dermatitis, hepatitis, and endocrinopathy should be considered to be immune-related.

Dose modification recommendations and toxicity management guidelines for immune-mediated reactions, for infusion-related reactions, and for non-immune-mediated reactions are detailed in Appendix B, Tables 1, 2, and 3 respectively.

In addition, management guidelines for adverse events of special interest (AESIs) are detailed in Appendix B, Table 1. All toxicities will be graded according to NCI CTCAE v4.03.

6.2 Study Restrictions and Concomitant Treatment

6.2.1 Contraception

Females of childbearing potential who are sexually active with a nonsterilised male partner must use 1 highly effective method of contraception from screening, and must agree to continue using such precautions for 90 days after the final dose of investigational product; cessation of birth control after this point should be discussed with a responsible physician. Non-sterilized male partners of a female patient must use male condom plus spermicide throughout this period. Periodic abstinence, the rhythm method, and the withdrawal method are not acceptable methods of birth control. Female patients should also refrain from breastfeeding throughout this period.

- Females of childbearing potential are defined as those who are not surgically sterile (i.e., bilateral tubal ligation, bilateral oophorectomy, or complete hysterectomy) or postmenopausal.
- Women will be considered post-menopausal if they have been amenorrheic for 12 months without an alternative medical cause. The following age-specific requirements apply:
  - Women <50 years of age would be considered post-menopausal if they have been amenorrheic for 12 months or more following cessation of exogenous hormonal treatments and if they have luteinizing hormone and follicle-stimulating hormone levels in the post-menopausal range for the institution or underwent surgical sterilization (bilateral oophorectomy or hysterectomy).
  - Women ≥50 years of age would be considered post-menopausal if they have been amenorrheic for 12 months or more following cessation of all exogenous hormonal treatments, had radiation-induced menopause with last menses >1 year ago, had chemotherapy-induced menopause with last menses >1 year ago, or underwent surgical sterilization (bilateral oophorectomy, bilateral salpingectomy or hysterectomy).
- Highly effective methods of contraception, defined as one that results in a low failure rate (i.e., less than 1% per year) when used consistently and correctly are described in Table 2. Note that some

contraception methods are not considered highly effective (e.g., male or female condom with or without spermicide; female cap, diaphragm, or sponge with or without spermicide; non-copper containing intrauterine device; progestogen-only oral hormonal contraceptive pills where inhibition of ovulation is not the primary mode of action [excluding Cerazette/desogestrel which is considered highly effective]; and triphasic combined oral contraceptive pills).

- Nonsterilised males who are sexually active with a female partner of childbearing potential must use use male condom plus spermicide (see Table 1) from Day 1 and for 90 days after receipt of the final dose of investigational product.

Table 1. Highly Effective Methods of Contraception (<1% Failure Rate)

• Barrier/Intrauterine methods	• Hormonal Methods
• Copper T intrauterine device<br>• Levonorgestrel-releasing intrauterine system (e.g., Mirena®)[a]	• Implants: Etonogestrel-releasing implants: e.g. Implanon® or Norplant®<br>• Intravaginal: Ethinylestradiol/etonogestrel-releasing intravaginal devices: e.g. NuvaRing®<br>• Injection: Medroxyprogesterone injection: e.g. Depo-Provera®<br>• Combined Pill: Normal and low dose combined oral contraceptive pill<br>• Patch: Norelgestromin/ethinylestradiol-releasing transdermal system: e.g. Ortho Evra®<br>• Minipillc: Progesterone based oral contraceptive pill using desogestrel: Cerazette® is currently the only highly effective progesterone-based

a This is also considered a hormonal method

6.2.2 Blood donation

Subjects should not donate blood while participating in this study

6.2.3 Permitted Concomitant Medications

Investigators may prescribe concomitant medications or treatments (e.g., acetaminophen, diphenhydramine) deemed necessary to provide adequate prophylactic or supportive care except for those medications identified as “excluded” as listed in Section 6.2.4.

Table 2. Supportive Medications

Supportive medication/class of drug:	Usage:
Concomitant medications or treatments (e.g., acetaminophen or diphenhydramine) deemed necessary to provide adequate prophylactic or supportive care, except for those medications identified as "prohibited," as listed above	To be administered as prescribed by the Investigator
Best supportive care (including antibiotics, nutritional support, correction of metabolic disorders, optimal symptom control, and pain management [including palliative radiotherapy to non-target lesions, etc])	Should be used, when necessary, for all patients
Inactivated viruses, such as those in the influenza vaccine	Permitted

6.2.4 Excluded Concomitant Medications

The following medications are considered exclusionary during the study.

1. Any investigational anticancer therapy other than the protocol specified therapies
2. mAbs against CTLA-4, PD-1, or PD-L1 other than those under investigation in this study.
3. Any concurrent chemotherapy, radiotherapy, immunotherapy, biologic or hormonal therapy for cancer treatment, other than the protocol specified therapy. Concurrent use of hormones for noncancer-related conditions (e.g., insulin for diabetes and hormone replacement therapy) is acceptable.
4. Immunosuppressive medications including, but not limited to systemic corticosteroids at doses not exceeding 10 mg/day of prednisone or equivalent, methotrexate, azathioprine, and TNF-α blockers. Use of immunosuppressive medications for the management of investigational product-related AEs or in subjects with contrast allergies is acceptable. In addition, use of inhaled and intranasal corticosteroids is permitted. A temporary period of steroids will be allowed if clinically indicated and considered to be essential for the management of non-immunotherapy related events experienced by the patient (e.g., chronic obstructive pulmonary disease, radiation, nausea, etc).
5. Live attenuated vaccines within 30 days of durvalumab dosing (i.e., 30 days prior to the first dose, during treatment with durvalumab and for 30 days post discontinuation of durvalumab). Inactivated viruses, such as those in the influenza vaccine, are permitted.
6. EGFR TKIs should not be given concomitantly and should be used with caution in the 90 days post last dose of durvalumab. Increased incidences of pneumonitis (with third generation EGFR TKIs) and increased incidence of transaminase increases (with 1st generation EGFR TKIs) has been reported when durvalumab has been given concomitantly.
7. Herbal and natural remedies which may have immune-modulating effects.

7.0 ASSESSMENT OF SAFETY

7.1. Safety Parameters

7.1.1.1 Definition of adverse events

The International Conference on Harmonization (ICH) Guideline for Good Clinical Practice (GCP) E6 (R1) defines an AE as:

Any untoward medical occurrence in a patient or clinical investigation subject administered a pharmaceutical product and which does not necessarily have a causal relationship with this treatment. An AE can therefore be any unfavorable and unintended sign (including an abnormal laboratory finding), symptom, or disease temporally associated with the use of a medicinal product, whether or not considered related to the medicinal product.

An AE includes but is not limited to any clinically significant worsening of a subject's pre-existing condition. An abnormal laboratory finding (including ECG finding) that requires an action or intervention by the investigator, or a finding judged by the investigator to represent a change beyond the range of normal physiologic fluctuation, should be reported as an AE.

Adverse events may be treatment emergent (i.e., occurring after initial receipt of investigational product) or nontreatment emergent. A nontreatment-emergent AE is any new sign or symptom, disease, or other untoward medical event that begins after written informed consent has been obtained but before the subject has received investigational product.

Elective treatment or surgery or preplanned treatment or surgery (that was scheduled prior to the subject being enrolled into the study) for a documented pre-existing condition, that did not worsen from baseline, is not considered an AE (serious or nonserious). An untoward medical event occurring during the prescheduled elective procedure or routinely scheduled treatment should be recorded as an AE or SAE. The term AE is used to include both serious and non-serious AEs.

7.1.2 Definition of serious adverse events

A serious adverse event is an AE occurring during any study phase (i.e., screening, run-in, treatment, wash-out, follow-up), at any dose of the study drugs that fulfils one or more of the following criteria:

- Results in death
- Is immediately life-threatening
- Requires in-patient hospitalization or prolongation of existing hospitalization
- Results in persistent or significant disability or incapacity
- Is a congenital abnormality or birth defect in offspring of the subject
- Is an important medical event that may jeopardize the patient or may require medical intervention to prevent one of the outcomes listed above.

  Medical or scientific judgment should be exercised in deciding whether expedited reporting is appropriate in this situation. Examples of medically important events are intensive treatment in an emergency room or at home for allergic bronchospasm, blood dyscrasias, or convulsions that do not result in hospitalizations; or development of drug dependency or drug abuse.

The causality of SAEs (their relationship to all study treatment/procedures) will be assessed by the investigator(s) and communicated to AstraZeneca

7.1.3 Definition of adverse events of special interest (AESI)

An AESI is one of scientific and medical interest specific to understanding of the investigational product and may require close monitoring and rapid communication by the investigator to AstraZeneca/Medimmune. An AESI may be serious or nonserious. The rapid reporting of AESIs allows ongoing analysis of these events in order to characterize and understand them in association with the use of this investigational product.

AESIs for durvalumab include but are not limited to events with a potential inflammatory or immune-

mediated mechanism and which may require more frequent monitoring and/or interventions such as steroids, immunosuppressants and/or hormone replacement therapy. These AESIs are being closely monitored in clinical studies with durvalumab monotherapy and combination therapy. An immune-related adverse event (irAE) is defined as an adverse event that is associated with drug exposure and is consistent with an immune-mediated mechanism of action and where there is no clear alternate etiology. Serologic, immunologic, and histologic (biopsy) data, as appropriate, should be used to support an irAE diagnosis. Appropriate efforts should be made to rule out neoplastic, infectious, metabolic, toxin, or other etiologic causes of the irAE.
If the Investigator has any questions in regards to an adverse event (AE) being an irAE, the Investigator should promptly contact the Study Physician.
AESIs observed with durvalumab include:

- Diarrhea/Colitis and intestinal perforation
- Pneumonitis/ILD
- Hepatitis/transaminase increases
- Neuropathy / neuromuscular toxicity (Guillain-Barré, and myasthenia gravis)
- Endocrinopathy (i.e. events of hypophysitis, hypopituitarism adrenal insufficiency, , hyper- and hypothyroidism, and type I diabetes mellitus),
- Rash/Dermatitis
- Nephritis/blood creatinine increases
- Pancreatitis (serum lipase and amylase increases)
- Myocarditis
- Myositis/Polymyositis
- Intestinal perforations

Other inflammatory responses that are rare with a potential immune-mediated aetiology include, but are not limited to:

- Pericarditis
- Sarcoidosis
- Uveitis
- Other events involving the eye and skin
- Hematological events
- Rheumatological events
- Vasculitis
- Non-infectious meningitis
- Non-infectious encephalitis

It is possible that events with an inflammatory or immune mediated mechanism could occur in nearly all organs. In addition, infusion-related reactions and hypersensitivity/anaphylactic reactions with a different underlying pharmacological etiology are also considered AESIs.

Further information on these risks (e.g. presenting symptoms) can be found in the current version of the durvalumab Investigator Brochure.

7.1.4 Pneumonitis

Adverse events of pneumonitis are of interest for AstraZeneca/Medimmune, as pneumonitis has been reported with anti-PD-1 MAbs [51]. Initial work-up should include high-resolution CT scan, ruling out infection, and pulse oximetry. Pulmonary consultation is highly recommended.
Guidelines for the management of subjects with immune-mediated events including pneumonitis are outlined in Appendix B, Table 1.

7.1.5 Hypersensitivity reactions

Hypersensitivity reactions as well as infusion-related reactions have been reported with anti PD-L1 and anti-PD-1 therapy [51, 69]. As with the administration of any foreign protein and/or other biologic agents, reactions following the infusion of MAbs can be caused by various mechanisms, including acute anaphylactic (immunoglobulin E-mediated) and anaphylactoid reactions against the MAb, and serum sickness. Acute allergic reactions may occur, may be severe, and may result in death. Acute allergic reactions may include hypotension, dyspnea, cyanosis, respiratory failure, urticaria, pruritus, angioedema, hypotonia, arthralgia, bronchospasm, wheeze, cough, dizziness, fatigue, headache, hypertension, myalgia, vomiting and unresponsiveness.
Guidelines for management of subjects with hypersensitivity (including anaphylactic reaction) and infusion related reactions are outlined in Appendix B, Table 1.

7.1.6 Hepatic function abnormalities (hepatotoxicity)

Increased transaminases have been reported during treatment with anti-PD-L1/anti-PD-1 antibodies [69]. Inflammatory hepatitis has been reported in 3% to 9% of subjects treated with anti CTLA-4 monoclonal antibodies (e.g., ipilimumab). The clinical manifestations of ipilimumab-treated subjects included general weakness, fatigue, nausea and/or mild fever and increased liver function tests such as AST, ALT, alkaline phosphatase, and/or total bilirubin.
Hepatic function abnormality is defined as any increase in ALT or AST to greater than 3 × ULN and concurrent increase in total bilirubin to be greater than 2 × ULN. Concurrent findings are those that derive from a single blood draw or from separate blood draws taken within 8 days of each other. Follow-up investigations and inquiries will be initiated promptly by the investigational site to determine whether the findings are reproducible and/or whether there is objective evidence that clearly supports causation by a disease (e.g., cholelithiasis and bile duct obstruction with distended gallbladder) or an agent other than the investigational product. Guidelines for management of subjects with hepatic function abnormality are outlined in Appendix B, Table 1.
Cases where a subject shows an AST or ALT ≥3xULN or total bilirubin ≥ 2xULN may need to be reported as SAEs, These cases should be reported as SAEs if, after evaluation they meet the criteria for a Hy's Law case or if any of the individual liver test parameters fulfill any of the SAE criteria.

Criteria for Hy's Law (FDA Guidance 2009)

- The drug causes hepatocellular injury, generally shown by a higher incidence of 3 fold or greater elevations above the ULN of ALT or AST
- Among trial subjects showing such aminotransferase elevations, often with aminotransferases much greater than 3 x ULN, one or more also show elevation of serum total bilirubin to >2 x ULN, without initial findings of cholestasis (elevated serum alkaline phosphatase)
- No other reason can be found to explain the combination of increased aminotransferases and total bilirubin, such as viral hepatitis A, B, or C; pre-existing or acute liver disease; or another drug capable of causing the observed injury.

7.1.7 Gastrointestinal disorders

Diarrhea/colitis is the most commonly observed treatment emergent SAE when tremelimumab is used as monotherapy. In rare cases, colon perforation may occur that requires surgery (colectomy) or can lead to a fatal outcome if not properly managed. Guidelines on management of diarrhea and colitis in patients receiving durvalumab are provided in Table 1.

7.1.8 Endocrine disorders

Immune-mediated endocrinopathies include hypophysitis, adrenal insufficiency, and hyper- and hypothyroidism. Guidelines for the management of patients with immune-mediated endocrine events are provided in Table 1.

7.1.9 Pancreatic disorders

Immune-mediated pancreatitis includes autoimmune pancreatitis, and lipase and amylase elevation. Guidelines for the management of patients with immune-mediated pancreatic disorders are provided in Table 1.

7.1.10 Neurotoxicity

Immune-mediated nervous system events include encephalitis, peripheral motor and sensory neuropathies, Guillain-Barré, and myasthenia gravis. Guidelines for the management of patients with immune-mediated neurotoxic events are provided in Table 1.

7.1.11 Nephritis

Consult with Nephrologist. Monitor for signs and symptoms that may be related to changes in renal function (e.g. routine urinalysis, elevated serum BUN and creatinine, decreased creatinine clearance, electrolyte imbalance, decrease in urine output, proteinuria, etc). Patients should be thoroughly evaluated to rule out any alternative etiology (e.g., disease progression, infections etc.).
Steroids should be considered in the absence of clear alternative etiology even for low grade events (Grade 2), in order to prevent potential progression to higher grade event. Guidelines for the management of patients with immune-mediated neurotoxic events are provided in Table 1.

7.1.12 Radiation Risks

This is a non-ablative subclinical radiation dose that is expected to be associated with minimal or no risk.

7.2 Assessment of safety parameters

7.2.1 Adverse Event Characteristics

CTCAE term (AE description) and grade: The descriptions and grading scales found in the revised NCI Common Terminology Criteria for Adverse Events (CTCAE) version 4.0 will be utilized for AE reporting. A copy of the CTCAE version 4.0 can be downloaded from the CTEP web site (http://ctep.cancer.gov).

Attribution of the AE:

- Definite – The AE is clearly related to the study treatment.
- Probable – The AE is likely related to the study treatment.
- Possible – The AE may be related to the study treatment.
- Unlikely – The AE is doubtfully related to the study treatment.
- Unrelated – The AE is clearly NOT related to the study treatment.

7.3 Recording of Adverse Events

All adverse events will be recorded on a patient specific adverse event log. The AE log will be maintained by the research staff and kept in the patient’s research chart.

Adverse events will be recorded using a recognized medical term or diagnosis that accurately reflects the event. Adverse events will be assessed by the investigator for severity, relationship to the investigational product, possible etiologies, and whether the event meets criteria of an SAE and therefore requires immediate notification to AstraZeneca/MedImmune Patient Safety.
The following variables will be collected for each AE:

- AE (verbatim)
- The date when the AE started and stopped
- Changes in NCI CTCAE grade and the maximum CTC grade attained

- Whether the AE is serious or not
- Investigator causality rating against durvalumab (yes or no)
- Action taken with regard to durvalumab
- Outcome

In addition, the following variables will be collected for SAEs as applicable:

- Date AE met criteria for serious AE
- Date Investigator became aware of serious AE
- Reason AE is serious
- Date of hospitalization
- Date of discharge
- Probable cause of death
- Date of death
- Autopsy performed
- Description of AE
- Causality assessment in relation to Study procedure(s

7.3.1 Study recording period and follow-up for AEs and SAEs

Adverse events and serious adverse events will be recorded from time of signature of informed consent, throughout the treatment period and including the follow-up period (90 days after the last dose of durvalumab or initiation of new anti-cancer therapy, whichever occurs first). During the course of the study all AEs and SAEs should be proactively followed up for each subject. Every effort should be made to obtain a resolution for all events, even if the events continue after discontinuation/study completion.

The investigator is responsible for following all SAEs until resolution, until the subject returns to baseline status, or until the condition has stabilized with the expectation that it will remain chronic, even if this extends beyond study participation.

Follow-up of unresolved adverse events

Any AEs that are unresolved at the subject's last visit in the study are followed up by the investigator for as long as medically indicated, but without further recording in the eCRF. After 90 days, only subjects with ongoing investigational product-related SAEs will continue to be followed for safety. AstraZeneca/MedImmune retains the right to request additional information for any subject with ongoing AE(s)/SAE(s) at the end of the study, if judged necessary.

7.3.2 Serious Adverse Event (SAE) Reporting

7.3.3 Reporting of SAE to IRB

All SAEs occurring on this study will be reported to the IRB according to the IRB policy. The IRB requires immediate reporting of all unexpected and study-related (definite or probable) adverse events. The following procedure will be followed for reporting SAE to the IRB:

- Complete the SAE Cover Sheet (See Appendix C)
- If the event is unexpected AND definitely or probably related to the study, complete the IRB Unexpected, Study-related Adverse Events, Incidents, and Information Reporting Form. This form should be submitted within 24 hours of investigator notification of the event.
- If the event is expected OR possibly or unrelated to the study, only the SAE Cover Sheet will be completed. These events will be reported to the IRB at the time of continuing renewal on the Adverse Event & IND Safety Reporting Cumulative Table.

Forms may also be downloaded from the IRB website at:
http://www.med.cornell.edu/research/for_pol/ins_rev_boa.html

7.3.4 Reporting of SAE to FDA
All SAEs will be reported, whether or not considered causally related to the investigational product, or to the study procedure(s). The investigator is responsible for informing the IRB of the SAE as per section 7.5.2. The reporting period for SAEs is the period immediately following the time that written informed consent is obtained through 90 days after the last dose of durvalumab or until the initiation of alternative anticancer therapy.
The Investigator will inform the FDA, via a MedWatch/AdEERs form, of any serious or unexpected adverse events that occur in accordance with the reporting obligations of 21 CFR 312.32, and will concurrently forward all such reports to AstraZeneca. A copy of the MedWatch/AdEERs report will be faxed to AstraZeneca at the time the event is reported to the FDA. It is the responsibility of the investigator to compile all necessary information and ensure that the FDA receives a report according to the FDA reporting requirement timelines and to ensure that these reports are also submitted to AstraZeneca at the same time.
* A *cover page* should accompany the *MedWatch/AdEERs* form indicating the following:

- "Notification from an Investigator Sponsored Study"
- The investigator IND number assigned by the FDA
- The investigator's name and address
- The trial name/title and AstraZeneca ISS reference number (ESR-##-#####)

* The investigator will also indicate, either in the SAE report or the cover page, the *causality* of events *in relation to all study medications* and if the SAE is *related to disease progression*, as determined by the principal investigator.
** Send SAE report and accompanying cover page by way of email to AstraZeneca's designated mailbox:* AEMailboxClinicalTrialTCS@astrazeneca.com

If a non-serious AE becomes serious, this and other relevant follow-up information will also be provided to AstraZeneca and the FDA.
Serious adverse events that do not require expedited reporting to the FDA still need to be reported to AstraZeneca preferably using the MedDRA coding language for serious adverse events. This information should be reported on a monthly basis and under no circumstance less frequently than quarterly.

7.3.5 Reporting of deaths
All deaths that occur during the study or within the protocol-defined 90-day post-last dose of durvalumab safety follow-up period will be reported as follows:

- Death that is clearly the result of disease progression should be documented but should not be reported as an SAE.
- Where death is not due (or not clearly due) to progression of the disease under study, the AE causing the death will be reported to as a SAE within 24 hours. The report should contain a comment regarding the co-involvement of progression of disease, if appropriate, and should assign main and contributory causes of death.
- Deaths with an unknown cause should always be reported as a SAE. .
- Deaths that occur following the protocol-defined 90-day post-last-dose of durvalumab safety follow-up period will be documented, but will not be reported as an SAE.

7.3.6 Other events requiring reporting

7.3.7 Overdose

An overdose is defined as a subject receiving a dose of durvalumab in excess of that specified in the Investigator's Brochure, unless otherwise specified in this protocol.

Any overdose of a study subject with durvalumab, with or without associated AEs/SAEs, is required to be reported within 24 hours of knowledge of the event to AstraZeneca/MedImmune Patient Safety or designee using the designated Safety e-mailbox AEMailboxClinicalTrialTCS@astrazeneca.com. If the overdose results in an AE, the AE will also be recorded as an AE. Overdose does not automatically make an AE serious, but if the consequences of the overdose are serious, for example death or hospitalization, the event is serious and will be recorded and reported as an SAE. There is currently no specific treatment in the event of an overdose of durvalumab. The investigator will use clinical judgment to treat any overdose.

7.3.8 Hepatic function abnormality

Hepatic function abnormality (as defined section 7.1.6) in a study subject, with or without associated clinical manifestations, is required to be reported as "hepatic function abnormal" *within 24 hours of knowledge of the event* to AstraZeneca/MedImmune Patient Safety using the designated Safety e-mailbox, unless a definitive underlying diagnosis for the abnormality (e.g., cholelithiasis or bile duct obstruction) that is unrelated to investigational product has been confirmed.

- If the definitive underlying diagnosis for the abnormality has been established and is unrelated to investigational product, the decision to continue dosing of the study subject will be based on the clinical judgment of the investigator.
- If no definitive underlying diagnosis for the abnormality is established, dosing of the study subject must be interrupted immediately. Follow-up investigations and inquiries will be initiated by the Investigator without delay.

Each reported event of hepatic function abnormality will be followed by the investigator and evaluated by AstraZeneca.

7.3.9 Pregnancy

Maternal exposure

If a patient becomes pregnant during the course of the study, the IPs should be discontinued immediately.

Pregnancy itself is not regarded as an AE unless there is a suspicion that the IP under study may have interfered with the effectiveness of a contraceptive medication. Congenital abnormalities or birth defects and spontaneous miscarriages should be reported and handled as SAEs. Elective abortions without complications should not be handled as AEs. The outcome of all pregnancies (spontaneous miscarriage, elective termination, ectopic pregnancy, normal birth, or congenital abnormality) should be followed up and documented even if the patient was discontinued from the study.

If any pregnancy occurs in the course of the study, then the Investigator or other site personnel should inform the appropriate AstraZeneca representatives within 1 day, ie, immediately, but **no later than 24 hours** of when he or she becomes aware of it.

The designated AstraZeneca representative will work with the Investigator to ensure that all relevant information is provided to the AstraZeneca Patient Safety data entry site within 1 to 5 calendar days for SAEs and within 30 days for all other pregnancies.

The same timelines apply when outcome information is available.

7.3.10 Paternal exposure

Male patients should refrain from fathering a child or donating sperm during the study and for 90 days after the last dose of durvalumab monotherapy.

Pregnancy of the patient's partner is not considered to be an AE. However, the outcome of all pregnancies (spontaneous miscarriage, elective termination, ectopic pregnancy, normal birth, or congenital abnormality) occurring from the date of the first dose until 90 days after the last dose should, if possible, be followed up and documented.

Where a report of pregnancy is received, prior to obtaining information about the pregnancy, the Investigator will obtain the consent of the patient's partner. An IRB approved consent will be obtained prior to use.

7.4 Data Safety Monitoring Plan

Subjects will be carefully monitored and evaluated for adverse events by history, physical examination, and laboratory investigations prior to their scheduled durvalumab infusion and on a monthly basis after surgical resection for 4 months. All toxicities encountered during the study will be evaluated on an ongoing basis according to the NCI Common Toxicity Criteria version 4.0. All study treatment associated adverse events that are serious, at least possibly related and unexpected, and caused subject harm, will be reported to the IRB. Any modifications necessary to ensure subject safety and decisions to continue, or close the trial to accrual are also discussed during these meetings.

If there are 3 or more postoperative deaths in the first 20 patients this would be considered outside of our historical control, and thus the study would be stopped. The Weill Cornell Medical College DSMB will perform data and safety monitoring for this study quarterly. The Weill Cornell Medical College DSMB will review interim data to evaluate research subject safety, rates of accrual, and efficacy of the experimental intervention. After each evaluation, the Board will provide the principal investigator with recommendations for protocol modification, continuation or termination.

8. PHARMACEUTICAL INFORMATION

A list of the adverse events and potential risks associated with Investigational Agent can be found in Section 7.1.

8.1 Investigational Agent

8.1.1 Durvalumab

The Investigational Products Supply section of AstraZeneca/MedImmune will supply durvalumab to the investigator as a 500-mg vial solution for infusion after dilution.

8.1.2 Formulation/packaging/storage

Durvalumab will be supplied by AstraZeneca as a 500 mg vial solution for infusion after dilution. The solution contains 50 mg/mL durvalumab, 26 mM histidine/histidine hydrochloride, 275 mM trehalose dihydrate, and 0.02% (weight/volume) polysorbate 80; it has a pH of 6.0 and density of 1.054 g/ml. The nominal fill volume is 10 mL. Investigational product vials are stored at 2°C to 8°C (36°F to 46°F) and must not be frozen. Drug product should be kept in original packaging until use to prevent prolonged light exposure.

8.1.3 Study drug preparation

For patients weighing > 30 kg a fixed dose of durvalumab at 1.12 g every 3 weeks for 2 cycles will be administered pre-operatively and 1.5 g every 4 weeks for 12 cycles post-operatively.

For patients ≤ 30 kg weight based dosing is required. For the 1.5 g dose a 20 mg/kg dose Q4W would be used and for the 1.12 g dose a 15 mg/kg dose Q3W would be used.

Preparation of durvalumab doses for administration with an IV bag

The dose of durvalumab for administration will be prepared using aseptic technique. Total time from needle puncture of the durvalumab vial to the start of administration should not exceed:

- 24 hours at 2°C to 8°C (36°F to 46°F)
- 4 hours at room temperature

If in-use storage time exceeds these limits, a new dose must be prepared from new vials. Infusion solutions must be allowed to equilibrate to room temperature prior to commencement of administration.

No incompatibilities between durvalumab and polyvinylchloride or polyolefin IV bags have been observed.

A dose of 1.5 g durvalumab for patients >30 kg will be administered using an IV bag containing 0.9% (w/v) saline with a final durvalumab concentration ranging from 1 to 20 mg/mL, and delivered through an IV administration set with a 0.2- or 0.22-µm in-line filter. Add 30.0 mL of durvalumab (ie, 1.5 g of durvalumab) to the IV bag. The IV bag size should be selected such that the final concentration is within 1 to 20 mg/mL Mix the bag by gently inverting to ensure homogeneity of the dose in the bag.

A dose of 1.12 g durvalumab for patients >30 kg will be administered using an IV bag containing 0.9% (w/v) saline with a final durvalumab concentration ranging from 1 to 20 mg/mL, and delivered through an IV administration set with a 0.2- or 0.22-µm in-line filter. Add 22.4 mL of durvalumab (ie, 1.12 g of durvalumab) to the IV bag. The IV bag size should be selected such that the final concentration is within 1 to 20 mg/mL. Mix the bag by gently inverting to ensure homogeneity of the dose in the bag.

Weight-based dosing (for patients ≤ 30 kg) will be a administered using an IV bag containing 0.9% (w/v) saline with a final durvalumab concentration ranging from 1 to 20 mg/mL, and delivered through an IV administration set with a 0.2- or 0.22-µm in-line filter. For the 1.5 g dose a 20 mg/kg dose Q4W would be used and for the 1.12 g dose a 15 mg/kg dose Q3W would be used. The Appendix A includes an example of a weight-based dose calculation. Patient weight at baseline should be used for dosing calculations in patients ≤30 kg unless there is a ≥10% change in weight. Dosing day weight can be used for dosing calculations instead of baseline weight per institutional standard.

Durvalumab will be administered at room temperature by controlled infusion into a peripheral or central vein. Following preparation of durvalumab, the entire contents of the IV bag should be administered as an IV infusion over approximately 60 minutes (±5 minutes), using a 0.2 or 0.22-µm in-line filter. Less than 55 minutes is considered a deviation.

The IV line will be flushed with a volume of IV solution (0.9% [w/v] saline) equal to the priming volume of the infusion set used after the contents of the IV bag are fully administered, or complete the infusion according to institutional policy to ensure the full dose is administered and document if the line was not flushed.

Standard infusion time is 1 hour. However, if there are interruptions during infusion, the total allowed time should not exceed 8 hours at room temperature. The table below summarizes time allowances and temperatures.

Durvalumab hold and infusion times

Maximum time from needle puncture of vial to start of administration	4 hours at room temperature,<br>24 hours at 2°C to 8°C (36°F to 46°F)
Maximum time for IV bag infusion, including interruptions	8 hours at room temperature

In the event that either preparation time or infusion time exceeds the time limits outlined above, a new dose must be prepared from new vials. Durvalumab does not contain preservatives, and any unused portion must be discarded.

8.1.4 Monitoring of dose administration

Subjects will be monitored before, during and after the infusion with assessment of vital signs at the times specified in the Schedule of Assessment. Subjects are monitored (pulse rate, blood pressure) every 30 minutes during the infusion period (including times where infusion rate is slowed or temporarily stopped).

In the event of a ≤Grade 2 infusion-related reaction, the infusion rate of study drug may be decreased by 50% or interrupted until resolution of the event (up to 4 hours) and re-initiated at 50% of the initial rate until completion of the infusion. For subjects with a ≤Grade 2 infusion-related reaction, subsequent infusions may be administered at 50% of the initial rate. Acetaminophen and/or an antihistamine (e.g., diphenhydramine) or equivalent medications per institutional standard may be administered at the discretion of the investigator. If the infusion-related reaction is Grade 3 or higher in severity, study drug will be discontinued. The standard infusion time is one hour, however if there are interruptions during infusion, the total allowed time from infusion start to completion of infusion should not exceed4 hours at room temperature. As with any antibody, allergic reactions to dose administration are possible. Appropriate drugs and medical equipment to treat acute anaphylactic reactions must be immediately available. For management of patients who experience an infusion reaction, please refer to the toxicity and management guidelines in Appendix B.

8.1.5 Agent Accountability

The investigator, or a responsible party designated by the investigator, will maintain a careful record of the inventory and disposition of all agents received from Sponsor on a Drug Accountability Record Form (DARF).

9.0 MEASUREMENT OF EFFECT

9.1 Assessment of disease-free survival

All patients will be followed in our thoracic oncology follow-up clinic for disease recurrence using history, physical examination and CT scanning every 6 months for 2 years , then yearly thereafter till year 5. This follow-up protocol represents our institutional standard of care. Recurrence will be histologically or cytologically confirmed whenever possible. For the purpose of this trial DFS is defined as confirmed disease recurrence or death from any cause.

9.2 Assessment of clinical response.

All patients will have pre-treatment and post-treatment standard imaging including a positron emission tomography (PET)/computerized tomography scan (PET/CT scan) to determine disease stage and assess response to therapy. All imaging will be downloaded to our imaging data evaluation and analysis laboratory (IDEAL); a state of the art imaging facility operated by the Weill Cornell Department of Radiology. Independent radiologists blinded to treatment allocation will assess tumor shrinkage using conventional RECIST criteria 1.1.

Complete Response (CR): Disappearance of all target lesions. Any pathological lymph nodes (whether target or non-target) must have reduction in short axis to <10 mm.

Partial Response (PR): At least a 30% decrease in the sum of diameters of target lesions, taking as reference the baseline sum diameters.

Progressive Disease (PD): At least a 20% increase in the sum of diameters of target lesions, taking as reference the smallest sum on study (this includes the baseline sum if that is the smallest on study). In addition to the relative increase of 20%, the sum must also demonstrate an absolute increase of at least 5 mm. (Note: the appearance of one or more new lesions is also considered progression).

Stable Disease (SD): Neither sufficient shrinkage to qualify for PR nor sufficient increase to qualify for PD, taking as reference the smallest sum diameters while on study.

9.3 Assessment of pathological response

Major Pathologic Response (MPR): ≤10% residual viable tumor in the resected specimen on histopathological examination.

Complete Pathological Response (CPR): The absence of tumor cells in the resected specimen on histopathological examination.

10.0 DATA REPORTING / REGULATORY CONSIDERATIONS

10.1 Data Collection

The data collection plan for this study is to utilize REDCap to capture all treatment, toxicity, and efficacy data for all enrolled patients.

10.1.1 REDCap

REDCap (Research Electronic Data Capture) is a free data management software system that is fully supported by the Weill-Cornell Medical Center CTSC. It is a tool for the creation of customized, secure data management systems that include Web-based data-entry forms, reporting tools, and a full array of security features including user and group based privileges, authentication using institution LDAP system, with a full audit trail of data manipulation and export procedures. REDCap is maintained on CTSC-owned servers that are backed up nightly and support encrypted (SSL-based) connections. Nationally, the software is developed, enhanced and supported through a multi-institutional consortium led by the Vanderbilt University CTSA.

10.2 Regulatory Considerations

All protocol amendments and consent form modifications will be made by the Principal Investigator. AstraZeneca will have the opportunity to review and approve the changes prior to submission of these changes to the local IRB and distribution to participating sites.

10.3 Ethical conduct of the study

The study will be performed in accordance with ethical principles that have their origin in the Declaration of Helsinki and are consistent with ICH/Good Clinical Practice, and applicable regulatory requirements Subject data protection.

11. STATISTICAL CONSIDERATIONS

11.1 Specific Aims

11.1.1 Primary Aim:

The primary aim of this study is to determine that subablative SBRT at a dose of 8Gyx3 given concurrently with neoadjuvant durvalumab will increase the major pathological response (MPR) proportion from an estimated 15% in Arm 1 (Durva alone) to 35% in Arm 2 (SBRT+Durvalumab). A two-group chi-square test with a 0.20 one-sided significance level will have approximately 83% power to detect the difference between the Arm 1 MPR proportion of 0.15 and the arm 2 MPR proportion of 0.35 when the sample size is 30 in each arm.

11.1.2 Secondary Aim:

The first secondary aim of this study is to demonstrate that the use of durvalumab (with/without radiation) is associated with an improvement in the 2-year disease-free survival (DFS) proportion from 75% (historical control) to approximately 88% with the use of the new antibody. The historical control rate was determined from the DFS rate reported by Strauss et al from CALGB 9633, a US based randomized trial of adjuvant chemotherapy versus observation in patients with pathological stages IB and II NSCLC [34]. The reported 2-year DFS was 68% in the observation arm and 75% in the chemotherapy arm [34]. Our institutional data are consistent with these results. Among patients in our database who were assigned a clinical stage, there were 215 patients with clinical stages IB and II some of whom received adjuvant chemotherapy. DFS for these 215 patients is shown in Figure 2 and was 75% at 2 years. We did not use DFS reported from other randomized trials since these were international trials and approximately 20-25% of patients enrolled had stage IIIA disease. In our proposed trial, an improvement in DFS by 13% (75% to 88% at 2 years) represents a 17% relative improvement in DFS; a difference that we believe to be both achievable and clinically meaningful. These results will necessarily lead to larger randomized trials for further confirmation.

Another secondary aim is to compare the clinical response between patients receiving the durvalumab alone (Arm 1) and patients receiving the antibody with radiation (Arm 2). Assuming clinical response proportions of 15% and 35% in Arms 1 and 2, respectively, 95% confidence intervals for the clinical response proportions can be constructed to be within ± 12.8% and ± 17.1% of the observed clinical response proportions in each arm, respectively.

A third secondary aim is to determine the safety of 2 cycles of preoperative durvalumab with and without radiation followed by surgery and adjuvant durvalumab for 12 months post-operatively.

The fourth secondary aim (exploratory) is to compare DFS between the two arms; however, this comparison will be for exploratory purposes only (this comparison will not be statistically powered to detect specific differences between the two arms).

11.2 Analysis Plan for Endpoints

11.2.1 Primary Endpoint:

The primary endpoint of the MPR proportion will be calculated for both treatment arms and 80% one-sided confidence intervals will be estimated via binomial proportions. The chi-square test or Fisher's exact test will be used, as appropriate, to compare the MPR proportions between the two arms. We will also generate the odds ratio and a corresponding 95% confidence interval to contrast the difference in MPR proportions between the two arms. Logistic regression will be used to generate the odds ratio and corresponding CI.

11.2.2 Secondary Endpoints:

The first secondary endpoint of 2-year DFS proportion will be calculated for the pooled group of patients (N=60) and a 95% confidence interval will be estimated via binomial proportions. An additional calculation will also be stratified by Arm 1 vs. Arm 2. The chi-square test or binomial exact test, whichever is appropriate, will be used to compare the 2-year DFS proportion in the 60 patients to the historical 2-year DFS proportion of 75%. The chi-square test or Fisher's exact test will be used, as appropriate, to compare the 2-year DFS proportion between the two arms.

The clinical response proportion will be calculated for both treatment arms and 95% confidence intervals will be estimated via binomial proportions. The chi-square test or Fisher's exact test will be used, as appropriate, to compare the clinical response proportions between the two arms.

The safety of durvalumab with and without low dose SBRT in the neoadjuvant setting in NSCLC will be investigated. All AEs in both arms ≥G3 with and without possible or probable attribution to study drug will be tabulated. The frequency of subjects experiencing toxicities will be tabulated for each arm. Toxicities will be assessed and graded according to CTCAE v. 4.0 terminology. Exact 95% confidence intervals around the toxicity proportions will be calculated to assess the precision of the obtained estimates. All analyses will be performed in SAS Version 9.4 (SAS Institute, Inc., Cary, North Carolina) and STATA Version 13.0 (Stata Corporation, College Station, Texas).

DFS will also be estimated using the Kaplan-Meier method, and 95% confidence intervals for DFS estimates will be calculated using Greenwood's formula. Median DFS will also be estimated along with a 95% confidence interval. These calculations will also be stratified by Arm 1 vs. Arm 2 for the purpose of the secondary aim. The log-rank test will be used to compare Kaplan-Meier DFS between the two arms (for exploratory purposes only; this comparison will not be statistically powered to detect specific differences between the two arms).

REFERENCES

1. Siegel, R., Ma, J., Zou, Z. & Jemal, A. Cancer statistics, 2014. Cancer J Clin: 64, 9-29, (2014).
2. Arriagada, R. et al. Long-term results of the international adjuvant lung cancer trial evaluating adjuvant Cisplatin-based chemotherapy in resected lung cancer. JCO 28, 35-42, (2010).
3. Winton T, Livingston R, Johnson D, Rigas J, Johnston M, Butts C, Cormier Y, Goss G, Inculet R, Vallieres E, et al. National Cancer Institute of Canada Clinical Trials Group; National Cancer Institute of the United States Intergroup JBR.10 Trial Investigators. Vinorelbine plus cisplatin vs. observation in resected non-small-cell lung cancer.N Engl J Med. 2005 Jun 23;352(25):2589-97.
4. Douillard, J. Y. et al. Adjuvant vinorelbine plus cisplatin versus observation in patients with completely resected stage IB-IIIA non-small-cell lung cancer: a randomised controlled trial. The Lancet. Oncology 7, 719-727,(2006).
5. Strauss GM, Herndon JE 2nd, Maddaus MA, Johnstone DW, Johnson EA, et al.Adjuvant paclitaxel plus carboplatin compared with observation in stage IB non-small-cell lung cancer: CALGB 9633 with the Cancer and Leukemia Group B, Radiation Therapy Oncology Group, and North Central Cancer Treatment Group Study Groups. J Clin Oncol. 2008 Nov 1;26(31):5043-51.
6. Lou, F., Sima, C. S., Rusch, V. W., Jones, D. R. & Huang, J. Differences in patterns of recurrence in early-stage versus locally advanced non-small cell lung cancer. The Annals of thoracic surgery 98.1755-1760 (2014).
7. Kiankhooy, A. et al. Predictors of early recurrence for node-negative t1 to t2b non-small cell lung cancer. The Annals of thoracic surgery 98, 1175-1183, (2014).
8. Radiant Trial. A randomized, double-blind phase 3 trial of adjuvant erlotinib versus placebo following complete tumor resection with or without aduvant chemotherapy in patients with stage IB-IIIA EGFR postive NSCLC ASCO (2014).
9. H.A. Wakelee, S.E. Dahlberg, S.M. Keller, W.J. Tester, D.R. Gandara, S.L. Graziano, A. Adjei, N. Leighl, S.C. Aisner, J.M. Rothman, J. Patel, M.D. Sborov, S.R. McDermott, R. Perez-Soler, A.M. Traynor, C. Butts, T. Evans, L. Horn, S.S. Ramalingam, J. Schiller . Randomized Phase III Trial of Adjuvant Chemotherapy with or without Bevacizumab in Resected Non-Small Cell Lung Cancer (NSCLC): Results of E1505 (. WCLC , Denver 2015.
10. Vansteenkiste, J., et. al & Altorki, N. A double-blind, randomized, placebo-controlled phase III study to assess the efficacy of reMAGE-A3 + AS15 antigen specific cancer immunotherapy as adjuvant therapy in patients wirh resectable MAGE A3-positive NSCLC. ESOM (2014).
11. Jure-Kundel, et al. Synergy between chemothrepautic agents and CTLA-4 blockade in preclinical tumor models. Cancer immunology, immunotherapy: CII 62, 1533-1545, (2013).
12. Masters, G., Barreto, L., Girit, E. & al., e. Antitumor activity of CTLA-4 blockade alone or combined with paclitaxel, etoposide, or gemcitabine in murine models. *J immunothera* **32**, 994 (2009).
13. Topalian, S., Sznol, M. & Brahmer, J. Anti-PD-L1 in patients with advanced solid tumors: survival and long-term safety in a phase I trial. *JCO:***31** (2013).
14. Gettinger SN, Horn L, Gandhi L, Spigel DR, Antonia SJ, Rizvi NA, Powderly JD, et al. Overall Survival and Long-Term Safety of Nivolumab (Anti-Programmed Death 1 Antibody, BMS-936558, ONO-4538) in Patients With Previously Treated Advanced Non-Small-Cell Lung Cancer. J Clin Oncol. 2015 Jun 20; 33(18):2004-12.
15. Garon EB, Rizvi NA, Hui R, Leighl N, Balmanoukian AS, Eder JP, Patnaik A, Aggarwal C, Gubens M, Horn L, Carcereny E, Ahn MJ, Felip E, Lee JS, Hellmann MD, Hamid O, Goldman JW, Soria JC, Dolled-Filhart M, Rutledge RZ, Zhang J, Lunceford JK, Rangwala R,

Lubiniecki GM, Roach C, Emancipator K, Gandhi L; KEYNOTE-001 Investigators.Pembrolizumab for the treatment of non-small-cell lung cancer.N Engl J Med. 2015 May 21;372(21):2018-28.
16. Rizvi NA, Mazières J, Planchard D, Stinchcombe TE, Dy GK, Antonia SJ, Horn L, Lena H, Minenza E, Mennecier B, Otterson GA, Campos LT, Gandara DR, Levy BP, Nair SG, et al. Activity and safety of nivolumab, an anti-PD-1 immune checkpoint inhibitor, for patients with advanced, refractory squamous non-small-cell lung cancer (CheckMate 063): a phase 2, single-arm trial. Lancet Oncol. 2015 Mar;16(3):257-65.
17. Herbst, R. S. *et al.* Predictive correlates of response to the anti-PD-L1 antibody MPDL3280A in cancer patients. *Nature* **515**, 563-567, (2014).
18. Naiyer A. Rizvi, Julie R. Brahmer, Sai-Hong Ignatius Ou, Neil Howard Segalet al; Safety and clinical activity of MEDI4736, an anti-programmed cell death-ligand 1 (PD-L1) antibody, in patients with non-small cell lung cancer (NSCLC). J Clin Oncol 33, 2015 (suppl; abstr 8032).
19. Zeng, A. P. See, J. Phallen et al., “Anti-PD-1 blockade and stereotactic radiation produce long-term survival in mice with intracranial gliomas,” International Journal of Radiation Oncology Biology Physics, vol. 86, no. 2, pp. 343–349, 2013.
20. Dewan, M. Z. et al. Fractionated but not single-dose radiotherapy induces an immune-mediated abscopal effect when combined with anti-CTLA-4 antibody. Clin Cancer Res 15, 5379-5388, (2009).
21. Demaria, S. et al. Ionizing radiation inhibition of distant untreated tumors (abscopal effect) is immune mediated. Int. J. Radiat. Oncol. Biol. Phys. 58, 862-870 (2004).
22. Dovedi SJ, Adlard AL, Lipowska-Bhalla G, McKenna C, Jones S, Cheadle EJ, Stratford IJ, Poon E, Morrow M, Stewart R, Jones H, Wilkinson RW, Honeychurch J, Illidge TM. Acquired resistance to fractionated radiotherapy can be overcome by concurrent PD-L1 blockade. Cancer Res. 2014 Oct 1;74(19):5458-68
23. Deng, L. et al. Irradiation and anti-PD-L1 treatment synergistically promote antitumor immunity in mice.JCI 124, 687-695, (2014).
24. Mu CY, Huang JA, Chen Y, Chen C, Zhang XG. High expression of PD-L1 in lung cancer may contribute to poor prognosis and tumor cells immune escape through suppressing tumor infiltrating dendritic cells maturation. Med Oncol. 2011 Sep; 28 (3):682-8.
25. Thompson RH, Gillett MD, Chevillem JC, Lohsem CM, Dong H, Webster WS, et al. Costimulatory molecule B7-H1 in primary and metastatic clear cell renal cell carcinoma. Cancer. 2005; 104(10):2084-91.
26. Thompson RH, Kuntz SM, Leibovich BC, Dong H, Lohse CM, Webster WS, et al. Tumor B7-H1 is associated with poor prognosis in renal cell carcinoma patients with long-term follow-up. Cancer Res. 2006;66(7):3381-5.
27. Krambeck AE, Dong H, Thompson RH, Kuntz SM, Lohse CM, Leibovich BC, et al. Survivin and B7-H1 are collaborative predictors of survival and represent potential therapeutic targets for patients with renal cell carcinoma. Clin Cancer Res. 2007;13(6):1749- 56.
28. Nomi T, Sho M, Akahori T, Hamada K, Kubo A, Kanehiro H, et al. Clinical significance and therapeutic potential of the programmed death-1 ligand/programmed death-1 pathway in human pancreatic adenocarcinoma. Clin Cancer Res. 2007 Apr 1;13(7):2151-7.
29. Loos M, Giese NA, Kleeff J, Giese T, Gaida MM, Bergmann F, et al. Clinical significance and regulation of the costimulatory molecule B7-H1 in pancreatic adenocarcinoma. Cancer Lett. 2008;268(1):98-109.
30. Wang L, Ma Q, Chen X, Guo K, Li J, Zhang M. Clinical significance of B7-H1 and B7-1 expressions in pancreatic carcinoma. World J Surg. 2010;34(5):1059-65.
31. Hamanishi J, Mandai M, Iwasaki M, Okazaki T, Tanaka Y, Yamaguchi K, et al. Programmed cell death 1 ligand 1 and tumor-infiltrating CD8+ T lymphocytes are prognostic factors of human ovarian cancer. Proc Nat Acad Sci U S A. 2007;104(9):3360-5.

32. Keir ME, Butte MJ, Freeman GJ, Sharpe AH. PD-1 and its ligands in tolerance and immunity. Annu Rev Immunol. 2008;26:677-704.
33. Park J, Omiya R, Matsumura Y, Sakoda Y, Kuramasu A, Augustine MM, et al. B7-H1/CD80 interaction is required for the induction and maintenance of peripheral T-cell tolerance. Blood. 2010;116(8):1291-8.
34. Zou W, Chen L. Inhibitory B7-family molecules in the tumour microenvironment. Nat Rev Immunol. 2008;8(6):467-77.
35. Blank C, Kuball J, Voelkl S, Wiendl H, Becker B, et al. Blockade of PD-L1 (B7-H1) augments human tumor-specific T cell responses in vitro. Int J Cancer. 2006 Jul 15;119(2):317-27.
36. F. Pages, J. Galon, M.C. Dieu-Nosjean, E. Tartour, C. Sautes-Fridman, W.H. Fridman Immune infiltration in human tumors: a prognostic factor that should not be ignored Oncogene, 29 (2010), pp. 1093–1102.
37. Hirano F1, Kaneko K, Tamura H, Dong H, Wang S, Ichikawa M, Rietz C, Flies DB, et al. Blockade of B7-H1 and PD-1 by monoclonal antibodies potentiates cancer therapeutic immunity. Cancer Res. 2005 Feb 1;65(3):1089-96.
38. Iwai Y1, Ishida M, Tanaka Y, Okazaki T, Honjo T, Minato N. Involvement of PD-L1 on tumor cells in the escape from host immune system and tumor immunotherapy by PD-L1 blockade. Proc Natl Acad Sci U S A. 2002 Sep 17;99(19):12293-7. Epub 2002 Sep 6.
39. Okudaira K, Hokari R, Tsuzuki Y, Okada Y, Komoto S, Watanabe C, et al. Blockade of B7-H1 or B7-DC induces an anti-tumor effect in a mouse pancreatic cancer model. Int J Oncol. 2009 Oct;35(4):741-9.
40. Zhang C, Wu S, Xue X, Li M, Qin X, Li W, Han W, Zhang Y. Anti-tumor immunotherapy by blockade of the PD-1/PD-L1 pathway with recombinant human PD-1-IgV. Cytotherapy. 2008;10(7):711-9.
41. Schmidinger M, Heina M, Zielinski C. Aldesleukin in advanced renal cell carcinoma. Expert Rev Anticancer Ther. 2004 Dec;4(6):957-80.
42. Madan R, Gulley J. Sipuleucel-T: harbinger of a new age of therapeutics for prostate cancer. Expert Rev Vaccines. 2011 Feb; 10(2): 141–150.
43. Fife BT, Bluestone JA. Control of peripheral T-cell tolerance and autoimmunity via the CTLA-4 and PD-1 pathways. Immunol Rev. 2008 Aug;224:166-82.
44. Dong H, Strome2 S, Diva, Tamura H, et al. Tumor-associated B7-H1 promotes T-cell apoptosis: A potential mechanism of immune evasion. Nature Medicine 8, 793 - 800 (2002)
45. Latchman YE, Liang SC, Wu Y, Chernova T, Sobel RA, Klemm M, Kuchroo VK, Freeman GJ, Sharpe AH. PD-L1-deficient mice show that PD-L1 on T cells, antigen-presenting cells, and host tissues negatively regulates T cells. Proc Natl Acad Sci U S A. 2004 Jul 20;101(29):10691-6.
46. Nishimura H, Minato N, Nakano T, Honjo T. Immunological studies on PD-1 deficient mice: implication of PD-1 as a negative regulator for B cell responses. Int Immunol. 1998 Oct;10(10):1563-72.
47. Nishimura H, Honjo T. PD-1: an inhibitory immunoreceptor involved in peripheral tolerance. Trends Immunol. 2001 May;22(5):265-8.
48. Brahmer JR, Drake CG, Wollner I, Powderly JD, Picus J, Sharfman WH, et al. Phase I study of single-agent anti-programmed death-1 (MDX-1106) in refractory solid tumors: safety, clinical activity, pharmacodynamics, and immunologic correlates. J Clin Oncol. 2010 Jul 1;28(19):3167-75.
49. Berger R., Rotem-Yehudar R., Slama G., Landes S., Kneller A., Leiba M., et al. (2008) Phase I safety and pharmacokinetic study of CT-011, a humanized antibody interacting with PD-1, in patients with advanced hematologic malignancies. Clin Cancer Res 14: 3044–3051.
50. Wolchok JD, Neyns B, Linette G, Negrier S, Lutzky J. Ipilimumab monotherapy in patients

with pretreated advanced melanoma: a randomised, double-blind, multicentre, phase 2, dose-ranging study. Lancet Oncol. 2010 Feb;11(2):155-64.
51. Topalian SL, Hodi FS, Brahmer JR, Gettinger SN, Smith DC, McDermott DF, et al. Safety,activity, and immune correlates of anti-PD-1 antibody in cancer. N Engl J Med. 2012 Jun 28;366(26):2443-54.
52. Ghiringhelli F, Apetoh L, Tesniere A, et al. Activation of the NLRP3 inflammasome in dendritic cells induces IL-1beta- dependent adaptive immunity against tumors. Nat Med. 2009;15: 1170 1178.
53. Liao YP, Wang CC, Butterfield LH, et al. Ionizing radiation affects human MART-1 melanoma antigen processing and presentation by dendritic cells. J Immunol. 2004;173:2462–2469.
54. Lee Y, Auh SL, Wang Y, et al. Therapeutic effects of ablative radiation on local tumor require CD8+ T cells: changing strategies for cancer treatment. Blood. 2009;114:589–595.
55. Dumitriu IE, Bianchi ME, Bacci M, et al. The secretion of HMGB1 is required for the migration of maturing dendritic cells. J Leukoc Biol. 2007;81:84–91.
56. Luo, B. & Lee, A. S. The critical roles of endoplasmic reticulum chaperones and unfolded protein response in tumorigenesis and anticancer therapies. Oncogene 32, 805-818, (2013).
57. M. Obeid, A. Tesniere, F. Ghiringhelli et al., "Calreticulin exposure dictates the immunogenicity of cancer cell death," Nature Medicine, vol. 13, no. 1, pp. 54–61, 2007.
58. Reits EA,HodgeJW,HerbertsCA,etal. Radiation modulates the peptide repertoire, enhances MHC class I expression, and induces successful antitumor immunotherapy. J Exp Med. 2006;203(5):1259 1271.
59. Sharma A, Bode B, Wenger RH, et al. Gamma-radiation promotes immunological recognition of cancer cells through increased expression of cancer-testis antigens in vitro and in vivo. PLoS One. 2011;6:e28217.
60. Chakraborty M, Abrams SI, Camphausen K, et al. Irradiation of tumor cells up-regulates Fas and enhances CTL lytic activity and CTL adoptive immunotherapy. J Immunol. 2003;170(12):6338-6347.
61. Gaugler MH, Squiban C, van der Meeren A, et al. Late and persistent up-regulation of intercellular adhesion molecule-1 (ICAM-1) expression by ionizing radiation in human endothelial cells in vitro. Int J Radiat Biol. 1997;72:201–209.
62. C.T.Garnett,C.Palena,M.Chakarborty,K.-Y.Tsang,J.Schlom, and J. W. Hodge, "Sublethal irradiation of human tumor cells modulates phenotype resulting in enhanced killing by cytotoxic T lymphocytes," Cancer Research, vol. 64, no. 21, pp. 7985–7994, 2004.
63. GabrilovichDI,NagarajS.Myeloid-derived suppressor cells as regulators of the immune system. Nat Rev Immunol. 2009;9(3):162-174.
64. SchaueD,XieMW,RatikanJA,McBrideWH. Regulatory T cells in radiotherapeutic responses. Front Oncol. 2012;2:90.
65. Barcellos-HoffMH,DerynckR,TsangML, Weatherbee JA. Transforming growth factor-beta activation in irradiated murine mammary gland. J Clin Invest. 1994;93(2):892-899.
66. Postow, M. A. et al. Immunologic correlates of the abscopal effect in a patient with melanoma. NEJM 366, 925-931, (2012).
67. Stamell EF, Wolchok JD, Gnjatic S, et al. The abscopal effect associated with a systemic anti-melanoma immune response. Int J Radiat Oncol Biol Phys. 2012;85:293–295.
68. Demaria S, Golden EB, Formenti SC. Role of Local Radiation Therapy in Cancer Immunotherapy. JAMA Oncol. 2015 Aug 13.
69. Brahmer JR, Tykodi SS, Chow LQM, Hwu WJ, Topalian SL, Hwu P, et al. Safety and activity of anti-PD-L1 antibody in patients with advanced cancer. N Engl J Med. 2012 Jun28;366(26):2455-65.
70. Spring LM, Fell G, Arfe A, Sharma C, Greenup RA, Reynolds KL, Smith BL, et al.

Pathological complete response after neoadjuvant chemotherapy and impact on breast cancer recurrence and survival: a comprehensive meta-analysis. Clin Cancer Res. 2020 Feb 11.

71. Forde PM, Chaft JE, Smith KN, Anagnostou V, Cottrell TR, Hellmann MD., et al. Neoadjuvant PD-1 Blockade in Resectable Lung Cancer. N Engl J Med. 2018 May 24;378(21):1976-1986.

Food and Drug Administration Guidance for Industry (issued July 2009) ‘Drug-induced liver injury: Premarketing clinical evaluation’. Available from URL: http://www.fda.gov/downloads/Drugs/GuidanceComplianceRegulatoryInformation/Guidances/UCM174090.pdf. Accessed 10 December 2013.

US Department of Health and Human Services, National Institutes of Health, National Cancer Institute. Common Terminology Criteria for Adverse Events Version 4.03. http://evs.nci.nih.gov/ftp1/CTCAE/About.html

References:

Antonia et al 2014a

Antonia S, Goldberg S, Balmanoukian A, Narwal R, Robbins P, D’Angelo G, et al. A Phase 1 open-label study to evaluate the safety and tolerability of MEDI4736, an anti-programmed cell death-ligand 1 (PD-L1) antibody, in combination with tremelimumab in patients with advanced non-small cell lung cancer (NSCLC). Poster presented at European Society of Medical Oncology (ESMO) Meeting, 2014 Sep 26-30; Madrid, Spain.

Narwal et al 2013

Narwal R, Roskos LK, Robbie GJ. Population pharmacokinetics of sifalimumab, an investigational anti-interferonalpha monoclonal antibody, in systemic lupus erythematosus. Clin Pharmacokinet 2013;52:1017–27.

Ng et al 2006

Ng CM, Lum BL, Gimenez V, Kelsey S, Allison D. Rationale for fixed dosing of pertuzumab in cancer patients based on population pharmacokinetic analysis. Pharm Res 2006;23(6):1275–84.

Wang et al 2009

Wang DD, Zhang S, Zhao H, Men AY, Parivar K. Fixed dosing versus body size based dosing of monoclonal antibodies in adult clinical trials. J Clin Pharmacol 2009;49(9):1012–24.

Zhang et al 2012

Zhang S, Shi R, Li C, Parivar K, Wang DD. Fixed dosing versus body size-based dosing of therapeutic peptides and proteins in adults. J Clin Pharmacol 2012;52(1):18–28.

APPENDIX A
DURVALUMAB DOSE CALCULATIONS

Durvalumab Dosing
The dosing should be done depending on subject weight (if subject is ≤ 30kg):

1. Cohort dose: X mg/kg

2. Subject weight: Y kg

3. Dose for subject: XY mg = X (mg/kg) × Y (kg)

4. Dose to be added into infusion bag:

Dose (mL) = XY mg / 50 (mg/mL)

where 50 mg/mL is durvalumab nominal concentration

The corresponding volume durvalumab should be rounded to the nearest tenth mL (0.1 mL). Dose adjustments for each cycle only needed for greater than 10% change in weight.

5. The number of vials required for dose preparation is the next greatest whole number of vials from the following formula:
Number of vials = Dose (mL) / 10 (mL/vial)

Please ensure the correct dose level is used. Below is an example for a 20 mg/kg weight-based dose

Example:

1. Cohort dose: 20 mg/kg

2. Subject weight: 30 kg

3. Dose for subject: 600 mg = 20 (mg/kg) × 30 (kg)

4. Dose to be added into infusion bag:

Dose (mL) = 600 mg / 50 (mg/mL) = 12.0 mL

5. The number of vials required for dose preparation:

Number of vials = 12.0 (mL) / 10.0 (mL/vial) = 2

APPENDIX B
DOSING MODIFICATIONS AND TOXICITY MANAGEMENT GUIDELINES
Tables 1, 2 and 3

**Dosing Modification and Toxicity Management Guidelines for Immune-mediated, Infusion Related, and Non Immune-mediated Reactions (MEDI4736 Monotherapy or Combination Therapy With Tremelimumab or Tremelimumab Monotherapy) 17 October 2019 Version**		
**General Considerations regarding Immune-Mediated Reactions**		
	**Dose Modifications**	**Toxicity Management**
Immune-related Adverse Events (Overall Management For toxicities not noted below)	Drug administration modifications of study drug/study regimen will be made to manage potential immune-related AEs based on severity of treatment-emergent toxicities graded per NCI CTCAE v4.03 (unless indicated otherwise.)<br>In addition to the criteria for permanent discontinuation of study drug/regimen based on CTC grade/severity (table below) , permanently discontinue study drug/study regimen for the following conditions:<br>• Inability to reduce corticosteroid to a dose of ≤10 mg of prednisone per day (or equivalent) **within 12 weeks** of the start of the immune-mediated adverse event (imAE)<br>• Grade 3 recurrence of a previously experienced treatment-related imAE following resumption of dosing.<br><br>Grade 1 No dose modification<br>Grade 2 Hold study drug/study regimen dose until Grade 2 resolution to ≤ Grade 1<br>• If toxicity worsens then treat as Grade 3 or Grade 4<br>•Study drug/study treatment can be resumed once event stabilizes to Grade ≤1 after completion of	It is recommended that management of immune-mediated Adverse Events (imAEs) follow the guidelines presented in this table<br>• It is possible that events with an inflammatory or immune mediated mechanism could occur in nearly all organs, some of them not noted specifically in these guidelines.<br>Whether specific immune-mediated events (and/or laboratory indicators of such events) are noted specifically in these guidelines or not, patients should be thoroughly evaluated to rule out any alternative etiology (e.g., disease progression, concomitant medications, infections, etc.) to a possible immune-mediated event. In the absence of a clear alternative etiology, all such events should be managed as if they were immune related. General recommendations follow.<br>Symptomatic and topical therapy should be considered for low-grade (Grade 1 or 2, unless otherwise specified) events<br>• For persistent (greater than 3 to 5 days) low-grade (Grade 2) or severe (Grade ≥3) events promptly start prednisone 1-2mg/kg/day PO or IV equivalent<br>• Some events with high likelihood for morbidity and/or mortality – e.g., myo-carditis, or other similar events even

		steroid taper<br>Patients with endocrinopathies who may require prolonged or continued steroid replacement can be retreated with study drug/study regimen on the following conditions: 1) the event stabilizes and is controlled , 2) the patient is clinically stable as per Investigator or treating physician's clinical judgement, and 3) doses of prednisone are at less than or equal to 10mg/day or equivalent.	if they are not currently noted in the guidelines – should progress rapidly to high dose IV corticosteroids (methylprednisolone at 2 to 4 mg/kg/day) even if the event is Grade 2, and if clinical suspicion is high and/or there has been clinical confirmation. Consider, as necessary, discussing with the study physician, and promptly pursue specialist consultation.<br>• If symptoms recur or worsen during corticosteroid tapering (28 days of taper), increase the corticosteroid dose (prednisone dose [e.g. up to 2-4mg/kg/day PO or IV equivalent]) until stabilization or improvement of symptoms, then resume corticosteroid tapering at a slower rate ($\geq$ 28 days of taper)<br>• More potent immunosuppressives such as TNF inhibitors (e.g. infliximab; also refer to the individual sections of the immune related adverse event for specific type of immunosuppressive) should be considered for events not responding to systemic steroids. Progression to use of more potent immunosuppressives should proceed more rapidly in events with high likelihood for morbidity and/or mortality – e.g., myocarditis, or other similar events even if they are not currently noted in the guidelines – when these events are not responding to systemic steroids.<br>• With long-term steroid and other immunosuppressive use, consider need for Pneumocystis jirovecii pneumonia (PJP, formerly known as Pneumocystis carinii pneumonia) prophylaxis, gastrointestinal protection, and glucose monitoring.<br>• Discontinuation of study drug is not mandated for Grade 3 / Grade 4 inflammatory reactions attributed to local tumour response (e.g. inflammatory reaction at sites of metastatic disease, lymph nodes etc.). Continuation of study drug in this situation should be based upon a benefit/risk analysis for that patient
	Grade 3	Depending on the individual toxicity, may permanently discontinue study drug/study regimen. Please refer to guidelines below	
	Grade 4	Permanently discontinue study drug/study regimen<br>Note: For asymptomatic amylase or lipase levels of >2.0×ULN, hold study drug/regimen and if complete work up shows no evidence of pancreatitis, study drug/study regimen may be continued or resumed.<br>Note: Study drug/study regimen should be permanently discontinued in Grade 3 events with high likelihood for morbidity and/or mortality – e.g., myocarditis, or other similar events even if they are not currently noted in the guidelines. Similarly, consider whether study drug/study regimen should be permanently discontinued in Grade 2 events with high likelihood for morbidity and/or mortality – e.g., myocarditis, or other similar events even if they are not currently noted in the guidelines – when they do not rapidly improve to Grade <1 upon treatment with systemic steroids and following full taper<br>Note: There are some exceptions to permanent discontinuation of study drug for Grade 4 events (i.e., hyperthyroidism, hypothyroidism, Type1 diabetes mellitus).	

**Dosing Modification and Toxicity Management Guidelines for Immune-mediated, Infusion Related, and Non Immune-mediated Reactions for Durvalumab Monotherapy**			
	**Grade of the Event (NCI CTCAE version 4.03)**	**Dose Modifications**	**Toxicity Management**
Pneumonitis//Interstitial Lung Disease (ILD)	Grade of Pneumonitis (CTCAE version 4.03)	General Guidance	• Monitor patients for signs and symptoms of pneumonitis or ILD (new onset or worsening shortness of breath or cough). Patients should be evaluated with imaging and pulmonary function tests including other diagnostic procedures as described below<br>• Initial work-up may include clinical evaluation, monitoring of oxygenation via pulse oximetry (resting and exertion), laboratory work-up and high-resolution CT scan.
	Grade 1<br>(Asymptomatic, clinical or diagnostic observations only, intervention not indicated)	No dose modification required. However, consider holding study drug/study regimen dosing as clinically appropriate and during diagnostic work-up for other etiologies	For Grade 1 (Radiographic Changes Only)<br>• Monitor and closely follow up in 2-4 days for clinical symptoms, pulse oximetry (resting and exertion) and laboratory work-up and then as clinically indicated<br>• Consider Pulmonary and Infectious Disease consults.
	Grade 2<br>(Symptomatic, medical intervention indicated, limiting instrumental ADL)	Hold study drug/study regimen dose until Grade 2 resolution to ≤ Grade 1<br>• If toxicity worsens then treat as Grade 3 or Grade 4<br>• If toxicity improves to ≤ Grade 1 then the decision to reinitiate study drug/regimen will be based upon treating physician's clinical judgment and after completion of steroid taper.	For Grade 2 (Mild to Moderate New Symptoms)<br>• Monitor symptoms daily and consider hospitalization<br>• Promptly start systemic steroids (e.g., prednisone 1-2mg/kg/day PO or IV equivalent)<br>• Reimaging as clinically indicated<br>• If no improvement within 3-5 days, additional workup should be considered and prompt treatment with IV methylprednisolone 2-4mg/kg/day started<br>• If still no improvement within 3-5 days despite IV

Dosing Modification and Toxicity Management Guidelines for Immune-mediated, Infusion Related, and Non Immune-mediated Reactions for Durvalumab Monotherapy			
	**Grade of the Event (NCI CTCAE version 4.03)**	**Dose Modifications**	**Toxicity Management**
			methylprednisolone at 2-4/g/kg/day, promptly start immunosuppressive therapy such as TNF inhibitors (e.g. infliximab at 5mg/kg every 2 weeks). Caution: Important to rule out sepsis and refer to infliximab label for general guidance before using infliximab<br>• Once improving, gradually taper steroids over ≥ 28 days and consider prophylactic antibiotics, antifungal or anti PJP treatment (refer to current NCCN guidelines for treatment of cancer-related infections).[a]<br>• Consider Pulmonary and Infectious Disease Consults<br>• Consider as necessary discussing with study physician
	Grade 3 or 4<br>(Grade 3: Severe symptoms; limiting self-care ADL; oxygen indicated;<br><br>Grade 4: life threatening respiratory compromise, urgent intervention indicated [e.g. tracheostomy or intubation])	Permanently discontinue study drug/study regimen	For Grade 3 or 4 (severe or new symptoms, new/worsening hypoxia, life threatening<br>• Promptly initiate empiric IV methylprednisolone 1 to 4 mg/kg/day or equivalent<br>• Obtain Pulmonary and Infectious Disease consults; consult, as necessary, discussing with study physician.<br>• Hospitalize the patient<br>• Supportive Care (oxygen, etc.)<br>• If no improvement within 3-5 days, additional workup should be considered and prompt treatment with additional immunosuppressive therapy such as TNF inhibitors (e.g. infliximab at 5mg/kg every 2 weeks dose) started. Caution: rule out sepsis and refer to infliximab label for general guidance before using infliximab

Dosing Modification and Toxicity Management Guidelines for Immune-mediated, Infusion Related, and Non Immune-mediated Reactions for Durvalumab Monotherapy			
	**Grade of the Event (NCI CTCAE version 4.03)**	**Dose Modifications**	**Toxicity Management**
			• Once improving, gradually taper steroids over ≥28 days and consider prophylactic antibiotics, antifungals and in particular, anti PJP treatment (please refer to current NCCN guidelines for treatment of cancer-related infections)[i]
Diarrhea/ Colitis<br>Large intestine perforation/Intestine perforation	Grade of Diarrhea (CTCAE version 4.03)	General Guidance	− Monitor for symptoms that may be related to diarrhea/enterocolitis (abdominal pain, cramping, or changes in bowel habits such as increased frequency over baseline or blood in stool) or related to bowel perforation (such as sepsis, peritoneal signs and ileus)<br>− When symptoms or evaluation indicate a perforation is suspected, consult a surgeon experienced in abdominal surgery immediately without any delay.<br>− Patients should be thoroughly evaluated to rule out any alternative etiology (e.g., disease progression, other medications, infections including testing for clostridium difficile toxin, etc.)<br>− Steroids should be considered in the absence of clear alternative etiology, even for low grade events, in order to prevent potential progression to higher grade event, including perforation.<br>− Use analgesics carefully; they can mask symptoms of perforation and peritonitis

Dosing Modification and Toxicity Management Guidelines for Immune-mediated, Infusion Related, and Non Immune-mediated Reactions for Durvalumab Monotherapy			
	**Grade of the Event (NCI CTCAE version 4.03)**	**Dose Modifications**	**Toxicity Management**
	Grade 1 Diarrhea: (stool frequency of <4 over baseline per day)<br>(Colitis: asymptomatic; clinical or diagnostic observations only)	No dose modification	For Grade 1 diarrhea :<br>− Close monitoring for worsening symptoms<br>− Consider symptomatic treatment including hydration, electrolyte replacement, dietary changes (e.g., American Dietetic Association colitis diet), and loperamide. Use of probiotics as per treating physician's clinical judgment.
	Grade 2 Diarrhea: (stool frequency of 4-6 over baseline per day) (Colitis: abdominal pain; mucus or blood in stool)<br>(Perforation: symptomatic; medical intervention indicated*)	Hold study drug/study regimen until resolution to ≤ Grade 1<br>• If toxicity worsens then treat as Grade 3 or Grade 4<br>• If toxicity improves to < Grade 1 then study drug/study regimen can be resumed after completion of steroid taper.	For Grade 2 diarrhea:<br>− Consider symptomatic treatment including hydration, electrolyte replacement, dietary changes (e.g., American Dietetic Association colitis diet), and loperamide and/or budesonide<br>− Promptly start prednisone 1 to 2 mg/kg/day PO or IV equivalent<br>− If event is not responsive within 3-5 days or worsens despite prednisone at 1-2 mg/kg/day PO or IV equivalent, GI consult should be obtained for consideration of further workup such as imaging and/or colonoscopy to confirm colitis and rule out perforation, and prompt treatment with IV methylprednisolone 2-4mg/kg/day started.<br>− If still no improvement within 3-5 days despite 2-4mg/kg IV methylprednisolone, promptly start

Dosing Modification and Toxicity Management Guidelines for Immune-mediated, Infusion Related, and Non Immune-mediated Reactions for Durvalumab Monotherapy			
	**Grade of the Event (NCI CTCAE version 4.03)**	**Dose Modifications**	**Toxicity Management**
	* "medical intervention" is not invasive		immunosuppressives such as (infliximab at 5mg/kg once every 2 weeks[1]). . **Caution**: Important to rule out bowel perforation and refer to infliximab label for general guidance before using infliximab<br>– Consider, as necessary, discussing with study physician if no resolution to ≤ Grade 1 in 3 to 4 days.<br>– Once the patient is improving, gradually taper steroids over ≥28 days and consider prophylactic antibiotics, antifungals and anti PJP treatment (please refer to current NCCN guidelines for treatment of cancer-related infections).

Dosing Modification and Toxicity Management Guidelines for Immune-mediated, Infusion Related, and Non Immune-mediated Reactions for Durvalumab Monotherapy			
	**Grade of the Event (NCI CTCAE version 4.03)**	**Dose Modifications**	**Toxicity Management**
	Grade 3 or 4 diarrhea<br><br>(Grade 3 Diarrhea: Stool frequency of ≥7 over baseline per day;<br><br>Grade 4 Diarrhea: life threatening consequences)<br>(Grade 3 Colitis: severe abdominal pain, change in bowel habits, medical intervention indicated, peritoneal signs;<br>Grade 4 Colitis: life-threatening consequences, urgent intervention indicated) (Grade 3 Perforation: severe symptoms,	Grade 3<br>Permanently discontinue study drug/study regimen- for Grade 3 if toxicity does not improve to Grade ≤1 within 14 days; study drug/study regimen can be resumed after completion of steroid taper.<br><br>Grade 4<br>Permanently discontinue<br>study drug/study regimen.	For Grade 3 or 4 diarrhea:<br>– Promptly initiate empiric IV methylprednisolone 2 to 4 mg/kg/day or equivalent<br>– Monitor stool frequency and volume and maintain hydration<br>– Urgent GI consult and imaging and/or colonoscopy as appropriate<br>– If still no improvement within 3-5 days of IV methylprednisolone 2 to 4mg/kg/day or equivalent, promptly start further immunosuppressives (e.g. infliximab at 5mg/kg once every 2 weeks). Caution: Ensure GI consult to rule out bowel perforation and refer to infliximab label for general guidance before using infliximab. If perforation is suspected, consult a surgeon experienced in abdominal surgery immediately without any delay.<br><br>– Once improving, gradually taper steroids over ≥28 days and consider prophylactic antibiotics, antifungals and anti PJP treatment (please refer to current NCCN guidelines for treatment of cancer-related infections).[a]

Dosing Modification and Toxicity Management Guidelines for Immune-mediated, Infusion Related, and Non Immune-mediated Reactions for Durvalumab Monotherapy			
	**Grade of the Event (NCI CTCAE version 4.03)**	**Dose Modifications**	**Toxicity Management**
	elective* operative intervention indicated; Grade 4 Perforation: life-threatening consequences, urgent intervention indicated)<br><br>*This guidance anticipates that Grade 3 operative interventions of perforations are usually not elective		
Hepatitis (Elevated LFTs)<br>**Infliximab should not be used for management of Immune Related Hepatitis**	**Any Elevations in AST, ALT or TB as Described Below**		**For Any Elevations Described:**<br>– Monitor and evaluate liver function test: AST, ALT, ALP and total bilirubin<br>– Evaluate for alternative etiologies (e.g., viral hepatitis, disease progression, concomitant medications)
	AST or ALT > ULN and ≤3.0×ULN if baseline normal, 1.5-3.0×baseline if baseline abnormal;	• No dose modifications.<br>If it worsens, then treat as described for elevations in the row below.	– Continue LFT monitoring per protocol

Dosing Modification and Toxicity Management Guidelines for Immune-mediated, Infusion Related, and Non Immune-mediated Reactions for Durvalumab Monotherapy			
	**Grade of the Event (NCI CTCAE version 4.03)**	**Dose Modifications**	**Toxicity Management**
	and/or TB > ULN and ≤1.5×ULN if baseline normal, >1.0-1.5×baseline if baseline abnormal		
	AST or ALT >3.0×ULN and ≤5.0×ULN if baseline normal, >3-5×baseline if baseline abnormal; and/or TB >1.5×ULN and ≤3.0×ULN if baseline normal, >1.5- 3.0×baseline if baseline abnormal	• Hold study drug/study regimen dose until resolution to AST or ALT ≤3.0×ULN and/or TB ≤1.5×ULN if baseline normal, or to AST or ALT ≤3.0×baseline and/or TB ≤1.5×baseline if baseline abnormal.<br>• If toxicity worsens, then treat as described for elevation in the row below.<br>If toxicity improves to AST or ALT ≤3.0×ULN and/or TB ≤1.5×ULN if baseline normal, or to AST or ALT ≤3.0×baseline and/or TB ≤1.5×baseline if baseline abnormal, resume study drug/study regimen after completion of steroid taper.	Regular and frequent checking of LFTs (e.g., every 1 to 2 days) until elevations of these are improving or resolved.<br>– If no resolution to AST or ALT ≤3.0×ULN and/or TB ≤1.5×ULN if baseline normal, or to AST or ALT ≤3.0×baseline and/or TB ≤1.5×baseline if baseline abnormal, in 1 to 2 days, consider, as necessary, discussing with study physician.<br>– If event is persistent (>3 to 5 days) or worsens, promptly start prednisone 1 to 2 mg/kg/day PO or IV equivalent.<br>– If still no improvement within 3 to 5 days despite 1 to 2 mg/kg/day of prednisone PO or IV equivalent, consider additional work up and start prompt treatment with IV methylprednisolone 2 to 4 mg/kg/day.<br>– If still no improvement within 3 to 5 days despite 2 to 4 mg/kg/day of IV methylprednisolone, promptly start immunosuppressives (i.e., mycophenolate mofetil).[a] Discuss with study physician if mycophenolate mofetil is not available. **Infliximab should NOT be used.**

Dosing Modification and Toxicity Management Guidelines for Immune-mediated, Infusion Related, and Non Immune-mediated Reactions for Durvalumab Monotherapy			
	**Grade of the Event (NCI CTCAE version 4.03)**	**Dose Modifications**	**Toxicity Management**
			– Once the patient is improving, gradually taper steroids over ≥28 days and consider prophylactic antibiotics, antifungals, and anti-PJP treatment (refer to current NCCN guidelines for treatment of cancer-related infections).[a]
	AST or ALT >5.0×ULN if baseline normal, >5×baseline if baseline abnormal; and/or TB >3.0×ULN if baseline normal; >3.0×baseline if baseline abnormal	For elevations in transaminases ≤8×ULN and/or in TB ≤5×ULN if baseline normal, or for elevations in transaminases ≤8×baseline and/or TB ≤5×baseline if baseline abnormal:<br>• Hold study drug/study regimen dose until resolution to AST or ALT ≤3.0×ULN and/or TB ≤1.5×ULN if baseline normal, or to AST or ALT ≤3.0×baseline and/or TB ≤1.5×baseline if baseline abnormal<br>• Resume study drug/study regimen if elevations downgrade to AST or ALT ≤3.0×ULN and/or TB ≤1.5×ULN if baseline normal, or to AST or ALT ≤3.0×baseline and/or TB ≤1.5×baseline if baseline abnormal, within 14 days and after completion of	– Promptly initiate empiric IV methylprednisolone at 1 to 4 mg/kg/day or equivalent.<br>– If still no improvement within 3 to 5 days despite 1 to 4 mg/kg/day methylprednisolone IV or equivalent, promptly start treatment with immunosuppressive therapy (i.e., mycophenolate mofetil). Discuss with study physician if mycophenolate is not available. **Infliximab should NOT be used.**<br>– Request Hepatology consult, and perform abdominal workup and imaging as appropriate.<br>– Once the patient is improving, gradually taper steroids over ≥28 days and consider prophylactic antibiotics, antifungals, and anti-PJP treatment (refer to current NCCN guidelines for treatment of cancer-related infections).[a]

Dosing Modification and Toxicity Management Guidelines for Immune-mediated, Infusion Related, and Non Immune-mediated Reactions for Durvalumab Monotherapy			
	**Grade of the Event (NCI CTCAE version 4.03)**	**Dose Modifications**	**Toxicity Management**
		steroid taper.<br>• Permanently discontinue study drug/study regimen if the elevations do not downgrade as described in bullet above within 14 days<br>For elevations in transaminases >8×ULN or elevations in TB >5×ULN if baseline normal, or for elevations in transaminases >8×baseline and/or TB >5×baseline if baseline abnormal, permanently discontinue study drug/study regimen.<br>Permanently discontinue study drug/study regimen for any case meeting Hy's law criteria (AST and/or ALT >3 × ULN + bilirubin >2 × ULN without initial findings of cholestasis (i.e., elevated alkaline P04) and in the absence of any alternative cause.[b]	

Dosing Modification and Toxicity Management Guidelines for Immune-mediated, Infusion Related, and Non Immune-mediated Reactions for Durvalumab Monotherapy			
	**Grade of the Event (NCI CTCAE version 4.03)**	**Dose Modifications**	**Toxicity Management**
Nephritis or Renal Dysfunction (Elevated Serum Creatinine)	Grade of Elevated Serum Creatinine (CTCAE version 4.03)<br><br>Any Grade	General Guidance	− Consult with Nephrologist<br>− Monitor for signs and symptoms that may be related to changes in renal function (e.g. routine urinalysis, elevated serum BUN and creatinine, decreased creatinine clearance, electrolyte imbalance, decrease in urine output, proteinuria, etc.)<br>− Patients should be thoroughly evaluated to rule out any alternative etiology (e.g., disease progression, infections etc.)<br>− Steroids should be considered in the absence of clear alternative etiology even for low grade events (Grade 2) , in order to prevent potential progression to higher grade event
	Grade 1 [Serum Creatinine > 1-1.5X baseline; > ULN to 1.5X ULN]	No dose modification	For Grade 1 elevated creatinine:<br>− Monitor serum creatinine weekly and any accompanying symptom<br>• If creatinine returns to baseline, resume its regular monitoring per study protocol.<br>• If it worsens, depending on the severity , treat as Grade 2 or Grade 3 or 4<br>- Consider symptomatic treatment including hydration, electrolyte replacement, diuretics, etc.

Dosing Modification and Toxicity Management Guidelines for Immune-mediated, Infusion Related, and Non Immune-mediated Reactions for Durvalumab Monotherapy			
	**Grade of the Event (NCI CTCAE version 4.03)**	**Dose Modifications**	**Toxicity Management**
	Grade 2 [Serum Creatinine>1.5-3.0X baseline; >1.5X-3.0XULN]	Hold study drug/study regimen until resolution to ≤ Grade 1 or baseline<br>• If toxicity worsens then treat as Grade 3 or Grade 4<br>• If toxicity improves to ≤ Grade 1 or baseline then resume study drug/study regimen after completion of steroid taper	For Grade 2 elevated creatinine:<br>– Consider symptomatic treatment including hydration, electrolyte replacement, diuretics, etc.<br>– Carefully monitor serum creatinine every 2-3 days and as clinically warranted<br>– Consult Nephrologist and consider renal biopsy if clinically indicated<br>– If event is persistent (> 3-5 days) or worsens, promptly start prednisone 1 to 2 mg/kg/day or IV equivalent<br>– If event is not responsive within 3-5 days or worsens despite prednisone at 1-2 mg/kg/day or IV equivalent, additional workup should be considered and prompt treatment with IV methylprednisolone at 2-4mg/kg/day started.<br>– Once improving gradually taper steroids over ≥28 days and consider prophylactic antibiotics, antifungals and anti PJP treatment (please refer to current NCCN guidelines for treatment of cancer-related infections.<br>– When event returns to baseline, resume study drug/study regimen and routine serum creatinine monitoring per study protocol.

**Dosing Modification and Toxicity Management Guidelines for Immune-mediated, Infusion Related, and Non Immune-mediated Reactions for Durvalumab Monotherapy**			
	**Grade of the Event (NCI CTCAE version 4.03)**	**Dose Modifications**	**Toxicity Management**
	Grade 3 or 4<br>(Grade 3: Serum Creatinine > 3.0 X baseline; >3.0-6.0 X ULN)<br><br>(Grade 4: Serum Creatinine > 6.0 X ULN)	Permanently discontinue study drug/study regimen	− Carefully monitor serum creatinine on daily basis<br><br>− Consult Nephrologist and consider renal biopsy if clinically indicated<br><br>− Promptly start prednisone 1 to 2 mg/kg/day or IV equivalent<br><br>− If event is not responsive within 3-5 days or worsens despite prednisone at 1-2 mg/kg/day or IV equivalent, additional workup should be considered and prompt treatment with IV methylprednisolone 2-4mg/kg/day started.<br><br>− Once improving, gradually taper steroids over ≥28 days and consider prophylactic antibiotics, antifungals and anti PJP treatment (refer to current NCCN guidelines for treatment of cancer-related infections.
**Rash or Dermatitis**<br>**(including Pemphigoid)**	Any Grade<br>(Please refer to NCICTCAE version 4.03 for definition of severity/grade depending on type of skin rash)	General Guidance	**For Any Grade:**<br>− Monitor for signs and symptoms of dermatitis (rash and pruritus).<br>− IF THERE IS ANY BULLOUS FORMATION, THE STUDY PHYSICIAN SHOULD BE CONTACTED **AND STUDY DRUG DISCONTINUED IF SUSPECT STEVENS-JOHNSON SYNDROME OR TOXIC EPIDERMAL NECROLYSIS.**

Dosing Modification and Toxicity Management Guidelines for Immune-mediated, Infusion Related, and Non Immune-mediated Reactions for Durvalumab Monotherapy			
	**Grade of the Event (NCI CTCAE version 4.03)**	**Dose Modifications**	**Toxicity Management**
	Grade 1	No dose modification	For Grade 1:<br>– Consider symptomatic treatment including oral antipruritics (e.g., diphenhydramine or hydroxyzine) and topical therapy (e.g., urea cream)
	Grade 2	For persistent (> 1- 2 weeks) Grade 2 events, hold scheduled study drug/study regimen until resolution to ≤ Grade 1 or baseline<br>• If toxicity worsens then treat as Grade 3<br>• If toxicity improves to Grade ≤1 or baseline, then resume drug/study regimen after completion of steroid taper	For Grade 2 :<br>– Obtain Dermatology consult<br>– Consider symptomatic treatment including oral antipruritics (e.g., diphenhydramine or hydroxyzine) and topical therapy (e.g., urea cream)<br>– Consider moderate-strength topical steroid<br>– If no improvement of rash/skin lesions occurs within 3-5 days or is worsening despite symptomatic treatment and/or use of moderate strength topical steroid, consider, as necessary, discussing with study physician and promptly start systemic steroids prednisone 1-2 mg/kg/day PO or IV equivalent<br>– Consider skin biopsy if persistent for >1-2 weeks or recurs
	Grade 3	Hold study drug/study regimen until resolution to ≤ Grade 1 or baseline<br>If temporarily holding the study drug/study regimen does not provide improvement of the Grade 3 skin rash to ≤ Grade 1 or baseline within 30 days, then permanently discontinue	For Grade 3 or 4:<br>– Consult Dermatology<br>– Promptly initiate empiric IV methylprednisolone 1 to 4 mg/kg/day or equivalent<br>– Consider hospitalization<br>– Monitor extent of rash [Rule of Nines]

Dosing Modification and Toxicity Management Guidelines for Immune-mediated, Infusion Related, and Non Immune-mediated Reactions for Durvalumab Monotherapy			
	**Grade of the Event (NCI CTCAE version 4.03)**	**Dose Modifications**	**Toxicity Management**
		Study drug/study regimen	− Consider skin biopsy (preferably more than 1) as clinically feasible.<br>− Once improving, gradually taper steroids over ≥28 days and consider prophylactic antibiotics, antifungals and anti PJP treatment (please refer to current NCCN guidelines for treatment of cancer-related infections)<br>− Consider, as necessary, discussing with Study Physician
	Grade 4	Permanently discontinue study drug/study regimen	
Endocrinopathy (e.g., hyperthyroidism, thyroiditis, hypothyroidism, Type 1 diabetes mellitus, hypophysitis, hypopituitarism, adrenal insufficiency.): exocrine event of amylase/lipase increased also included in this section)	Any Grade<br>(Depending on the type of endocrinopathy, refer to NCI CTCAE version 4.03 for defining the CTC grade/severity)	General Guidance	− Consider consulting an Endocrinologist for endocrine events.<br>− Consider, as necessary, discussing with study physician.<br>− Monitor patients for signs and symptoms of endocrinopathies. Non-specific symptoms include headache, fatigue, behavior changes, changed mental status, vertigo, abdominal pain, unusual bowel habits, polydipsia, polyuria hypotension and weakness.<br>− Patients should be thoroughly evaluated to rule out any alternative etiology (e.g., disease progression including brain metastases, infections, etc.)<br>− Depending on the suspected endocrinopathy, monitor and evaluate thyroid function tests: TSH, free T_3 and free T_4 and other relevant and related labs (e.g., blood glucose and ketone levels, HgA1c).<br>− For asymptomatic elevations in serum amylase and lipase >ULN and <3×ULN, corticosteroid treatment is not indicated as long as there are no other signs or symptoms of pancreatic inflammation.

Dosing Modification and Toxicity Management Guidelines for Immune-mediated, Infusion Related, and Non Immune-mediated Reactions for Durvalumab Monotherapy			
	**Grade of the Event (NCI CTCAE version 4.03)**	**Dose Modifications**	**Toxicity Management**
			– If a patient experiences an AE that is thought to be possibly of autoimmune nature (e.g., thyroiditis, pancreatitis, hypophysitis, diabetes insipidus), the investigator should send a blood sample for appropriate autoimmune antibody testing
	Grade 1<br>(Depending on the type of endocrinopathy, refer to NCI CTCAE version 4.03 for defining the CTC grade 1)	No dose modification	For Grade 1: (including those with asymptomatic TSH elevation)<br>– Monitor patient with appropriate endocrine function tests.<br>– For suspected hypophysitis/hypopituitarism, consider consultation of an endocrinologist to guide assessment of early-morning ACTH, cortisol, TSH and free T4; also consider gonadotropins, sex hormones, and prolactin levels, as well as cosyntropin stimulation test (though it may not be useful in diagnosing early secondary adrenal insufficiency).<br>– If TSH < 0.5X LLN, or TSH >2X ULN or consistently out of range in 2 subsequent measurements, include FT4 at subsequent cycles as clinically indicated and consider consultation of an endocrinologist.

Dosing Modification and Toxicity Management Guidelines for Immune-mediated, Infusion Related, and Non Immune-mediated Reactions for Durvalumab Monotherapy			
	**Grade of the Event (NCI CTCAE version 4.03)**	**Dose Modifications**	**Toxicity Management**
	Grade 2<br>(Depending on the type of endocrinopathy, refer to NCI CTCAE version 4.03 for defining the CTC grade/severity 2)	For Grade 2 endocrinopathy other than hypothyroidism and Type 1 diabetes mellitus hold study drug/study regimen dose until subject is clinically stable<br>• If toxicity worsens then treat as Grade 3 or Grade 4<br>•<br>Study drug/study regimen can be resumed once event stabilizes and after completion of steroid taper.<br>Patients with endocrinopathies who may require prolonged or continued steroid replacement (e.g. adrenal insufficiency) can be retreated with study drug/study regimen on the following conditions:<br>1) the event stabilizes and is controlled<br>2)) the patient is clinically stable as per Investigator or treating physician's clinical judgement,<br>3) Doses of prednisone are at less than or equal to 10mg/day or equivalent.	For Grade 2: (including those with symptomatic endocrinopathy)<br>– Consult endocrinologist to guide evaluation of endocrine function, and as indicated by suspected endocrinopathy and as clinically indicated, consider pituitary scan<br>– For all patients with abnormal endocrine work up, except those with isolated hypothyroidism or Type 1 DM, and as guided by an endocrinologist, consider short-term, corticosteroids (e.g., 1-2mg/kg/day methylprednisolone or IV equivalent) and prompt initiation of treatment with relevant hormone replacement (e.g. Levothyroxine, hydrocortisone, or sex hormones). -<br>– Once improving, gradually taper steroids over ≥28 days and consider prophylactic antibiotics, antifungals and anti PJP treatment (please refer to current NCCN guidelines for treatment of cancer-related infections)<br>– For patients with normal endocrine work up (lab or MRI scans), repeat labs/MRI as clinically indicated.

Dosing Modification and Toxicity Management Guidelines for Immune-mediated, Infusion Related, and Non Immune-mediated Reactions for Durvalumab Monotherapy			
	**Grade of the Event (NCI CTCAE version 4.03)**	**Dose Modifications**	**Toxicity Management**
	Grade 3 or 4<br>(Depending on the type of endocrinopathy, refer to NCI CTCAE version 4.03 for defining the CTC grade/severity 3 or 4)	For Grade 3 or 4 endocrinopathy other than hypothyroidism and Type 1 diabetes mellitus hold study drug/study regimen dose until endocrinopathy symptom(s) are controlled<br>Study drug/study regimen can be resumed once event stabilizes and after completion of steroid taper<br><br>Patients with endocrinopathies who may require prolonged or continued steroid replacement (e.g., adrenal insufficiency) can be retreated with study drug/study regimen on the following conditions:<br>1. The event stabilizes and is controlled.<br>2. The patient is clinically stable as per investigator or treating physician's clinical judgement.<br>3. Doses of prednisone are ≤10 mg/day or equivalent.	For Grade 3 or 4:<br>– Consult endocrinologist to guide evaluation of endocrine function and, as indicated by suspected endocrinopathy and as clinically indicated, consider pituitary scan. Hospitalization recommended.<br>– For all patients with abnormal endocrine work up, except those with isolated hypothyroidism<br>– Type 1 DM, and as guided by an endocrinologist, promptly initiate empiric IV methylprednisolone 1 to 2 mg/kg/day or equivalent<br>– as well as relevant hormone replacement (e.g. hydrocortisone, sex hormones)<br>– For adrenal crisis, severe dehydration, hypotension, or shock: immediately initiate intravenous corticosteroids with mineralocorticoid activity<br>– Isolated hypothyroidism may be treated with replacement therapy, without study drug/study regimen interruption, and without corticosteroids.<br>– Isolated Type 1 diabetes mellitus may be treated with appropriate diabetic therapy, without study drug/study regimen interruption, and without corticosteroids.<br>– Once patients on steroids are improving, gradually taper immunosuppressive steroids (as appropriate and with

Dosing Modification and Toxicity Management Guidelines for Immune-mediated, Infusion Related, and Non Immune-mediated Reactions for Durvalumab Monotherapy			
	**Grade of the Event (NCI CTCAE version 4.03)**	**Dose Modifications**	**Toxicity Management**
			guidance of endocrinologist) over ≥28 days and consider prophylactic antibiotics, antifungals and anti PJP treatment (please refer to current NCCN guidelines for treatment of cancer-related infections.
Neurotoxicity (to include but not limited to limbic encephalitis . autonomic neuropathy, excluding Myasthenia Gravis and Guillain-Barre)	Grade of Neurotoxicity<br>Depending on the type of neurotoxicity , refer to NCI CTCAE version 4.03 for defining the CTC grade/severity		
	Any Grade	General Guidance	– Patients should be evaluated to rule out any alternative etiology (e.g., disease progression, infections, metabolic syndromes and medications, etc.)<br>– Monitor patient for general symptoms (headache, nausea, vertigo, behavior change, or weakness)<br>– Consider appropriate diagnostic testing (e.g. electromyogram and nerve conduction investigations)

Dosing Modification and Toxicity Management Guidelines for Immune-mediated, Infusion Related, and Non Immune-mediated Reactions for Durvalumab Monotherapy			
	**Grade of the Event (NCI CTCAE version 4.03)**	**Dose Modifications**	**Toxicity Management**
			− Perform symptomatic treatment with Neurology consult as appropriate
	Grade 1	No dose modifications	See "Any Grade" recommendations above.
	Grade 2	• For acute motor neuropathies or neurotoxicity, hold study drug/study regimen dose until resolution to ≤ Grade 1<br>• For sensory neuropathy/neuropathic pain, consider holding study drug/study regimen dose until resolution to ≤ Grade 1.<br>o If toxicity worsens then treat as Grade 3 or Grade 4<br>• Study drug/study regimen can be resumed once event improves to Grade ≤1 and after completion of steroid taper	− Consider, as necessary, discussing with the study physician<br>− Obtain Neurology Consult<br>− Sensory neuropathy/neuropathic pain may be managed by appropriate medications (e.g., gabapentin, duloxetine, etc.)<br>− Promptly start systemic steroids prednisone 1-2mg/kg/day PO or IV equivalent<br>− If no improvement within 3-5 days despite 1-2mg/kg/day prednisone PO or IV equivalent consider additional workup and promptly treat with additional immunosuppressive therapy (e.g. IVIG)
	Grade 3	• Hold Study drug/study regimen dose until resolution to ≤ Grade 1<br>• Permanently discontinue study drug/study regimen if Grade 3 imAE does not resolve to ≤ Grade 1 within 30 days.	For Grade 3 or 4:<br>− Consider, as necessary, discussing with study physician<br>− Obtain Neurology Consult<br>− Consider hospitalization<br>− Promptly initiate empiric IV methylprednisolone 1 to 2

Dosing Modification and Toxicity Management Guidelines for Immune-mediated, Infusion Related, and Non Immune-mediated Reactions for Durvalumab Monotherapy			
	**Grade of the Event (NCI CTCAE version 4.03)**	**Dose Modifications**	**Toxicity Management**
	Grade 4	• Permanently discontinue study drug/study regimen	mg/kg/day or equivalent<br>− If no improvement within 3-5 days despite IV corticosteroids, consider additional workup and promptly treat with additional immunosuppressants (e.g. IVIG)<br>− Once stable, gradually taper steroids over ≥28 days
**Peripheral neuromotor syndromes** (such as Guillain-Barre and myasthenia gravis)		General Guidance	− The prompt diagnosis of immune-mediated peripheral neuromotor syndromes is important, since certain patients may unpredictably experience acute decompensations which can result in substantial morbidity or in the worst case, death. Special care should be taken for certain sentinel symptoms which may predict a more severe outcome, such as prominent dysphagia, rapidly progressive weakness, and signs of respiratory insufficiency or autonomic instability<br>− Patients should be evaluated to rule out any alternative etiology (e.g., disease progression, infections, metabolic syndromes and medications, etc.). It should be noted that the diagnosis of immune-mediated peripheral neuromotor syndromes can be particularly challenging in patients with underlying cancer, due to the multiple potential confounding effects of cancer (and its treatments) throughout the neuraxis. Given the importance of prompt and accurate diagnosis, it is essential to have a low threshold to obtain a Neurology consult<br>− Neurophysiologic diagnostic testing (e.g., electromyogram and nerve conduction investigations, and "repetitive

Dosing Modification and Toxicity Management Guidelines for Immune-mediated, Infusion Related, and Non Immune-mediated Reactions for Durvalumab Monotherapy			
	**Grade of the Event (NCI CTCAE version 4.03)**	**Dose Modifications**	**Toxicity Management**
			stimulation" if myasthenia is suspected) are routinely indicated upon suspicion of such conditions and may be best facilitated by means of a Neurology consultation<br>– It is important to consider that the use of steroids as the primary treatment of Guillain-Barre is not typically considered effective. Patients requiring treatment should be started with IVIG and followed by plasmapheresis if not responsive to IVIG
	Grade 1	No dose modification	– Consider, as necessary, discussing with the study physician<br>– Care should be taken to monitor patients for sentinel symptoms of a potential decompensation as described above<br>– Obtain a Neurology consult
	Grade 2	Hold study drug/study regimen dose until resolution to ≤ Grade 1<br>Permanently discontinue study drug/study regimen if it does not resolve to ≤ Grade 1 within 30 days or if there are signs of respiratory insufficiency or autonomic instability	Grade 2<br>– Consider, as necessary, discussing with the study physician<br>– Care should be taken to monitor patients for sentinel symptoms of a potential decompensation as described above<br>– Obtain a Neurology Consult<br>– Sensory neuropathy/neuropathic pain may be managed by appropriate medications (e.g., gabapentin, duloxetine, etc.)<br>*MYASTHENIA GRAVIS*<br>○ Steroids may be successfully used to treat Myasthenia

Dosing Modification and Toxicity Management Guidelines for Immune-mediated, Infusion Related, and Non Immune-mediated Reactions for Durvalumab Monotherapy			
	**Grade of the Event (NCI CTCAE version 4.03)**	**Dose Modifications**	**Toxicity Management**
			Gravis. Important to consider that steroid therapy (especially with high doses) may result in transient worsening of myasthenia and should typically be administered in a monitored setting under supervision of a consulting neurologist.<br>○ Patients unable to tolerate steroids may be candidates for treatment with plasmapheresis or IVIG. Such decisions are best made in consultation with a neurologist, taking into account the unique needs of each patient.<br>○ If Myasthenia Gravis-like neurotoxicity present, consider starting acetylcholine esterase (AChE) inhibitor therapy in addition to steroids. Such therapy, if successful, can also serve to reinforce the diagnosis.<br>*GUILLAIN-BARRE:*<br>○ Important to consider here that the use of steroids as the primary treatment of Guillain-Barre is not typically considered effective.<br>○ Patients requiring treatment should be started with IVIG and followed by plasmapheresis if not responsive to IVIG.
	Grade 3	Hold study drug/study regimen dose until resolution to ≤ Grade 1<br>Permanently discontinue Study drug/study regimen if Grade 3 irAE does not resolve to ≤ Grade 1 within 30 days or if there are signs of respiratory insufficiency or autonomic instability	For severe or life threatening (Grade 3 or 4) events:<br>– Consider, as necessary, discussing with study physician<br>– Recommend hospitalization<br>– Monitor symptoms and obtain Neurology consult<br>*MYASTHENIA GRAVIS*<br>○ Steroids may be successfully used to treat Myasthenia

Dosing Modification and Toxicity Management Guidelines for Immune-mediated, Infusion Related, and Non Immune-mediated Reactions for Durvalumab Monotherapy			
	**Grade of the Event (NCI CTCAE version 4.03)**	**Dose Modifications**	**Toxicity Management**
	Grade 4	Permanently discontinue study drug/study regimen	Gravis. It should typically be administered in a monitored setting under supervision of a consulting neurologist.<br>o Patients unable to tolerate steroids may be candidates for treatment with plasmapheresis or IVIG.<br>o If Myasthenia Gravis-like neurotoxicity present, consider starting acetylcholine esterase (AChE) inhibitor therapy in addition to steroids. Such therapy, if successful, can also serve to reinforce the diagnosis.<br>*GUILLAIN-BARRE*:<br>o Important to consider here that the use of steroids as the primary treatment of Guillain-Barre is not typically considered effective.<br>o Patients requiring treatment should be started with IVIG and followed by plasmapheresis if not responsive to IVIG
Myocarditis	Any Grade	Discontinue drug permanently if biopsy-proven immune-mediated myocarditis.	For Any Grade:<br>– The prompt diagnosis of immune-mediated myocarditis is important, particularly in patients with baseline cardiopulmonary disease and reduced cardiac function.<br>– Consider, as necessary, discussing with the study physician.<br>– Monitor patients for signs and symptoms of myocarditis (new onset or worsening chest pain,

**Dosing Modification and Toxicity Management Guidelines for Immune-mediated, Infusion Related, and Non Immune-mediated Reactions for Durvalumab Monotherapy**			
	**Grade of the Event (NCI CTCAE version 4.03)**	**Dose Modifications**	**Toxicity Management**
			arrhythmia, shortness of breath, peripheral edema). As some symptoms can overlap with lung toxicities, simultaneously evaluate for and rule out pulmonary toxicity as well as other causes (e.g., pulmonary embolism, congestive heart failure, malignant pericardial effusion). A Cardiology consultation should be obtained early, with prompt assessment of whether and when to complete a cardiac biopsy, including any other diagnostic procedures.<br>– Initial work-up should include clinical evaluation, BNP, cardiac enzymes, ECG, echocardiogram (ECHO), monitoring of oxygenation via pulse oximetry (resting and exertion), and additional laboratory work-up as indicated. Spiral CT or cardiac MRI can complement ECHO to assess wall motion abnormalities when needed.<br>– Patients should be thoroughly evaluated to rule out any alternative etiology (e.g., disease progression, other medications, or infections)
	Grade 1 (asymptomatic with laboratory [e.g., BNP] or cardiac imaging abnormalities)	No dose modifications required unless clinical suspicion is high, in which case hold study drug/study regimen dose during diagnostic work-up for other etiologies. If study drug/study regimen is held, resume after complete resolution to	For Grade 1 (no definitive findings):<br>-Monitor and closely follow up in 2 to 4 days for clinical symptoms, BNP, cardiac enzymes, ECG, ECHO, pulse oximetry (resting and exertion), and laboratory work-up as clinically indicated.

Dosing Modification and Toxicity Management Guidelines for Immune-mediated, Infusion Related, and Non Immune-mediated Reactions for Durvalumab Monotherapy			
	**Grade of the Event (NCI CTCAE version 4.03)**	**Dose Modifications**	**Toxicity Management**
	Grade 2, 3 or 4<br><br>(Grade 2: Symptoms with mild to moderate activity or exertion)<br><br>(Grade 3: Severe with symptoms at rest or with minimal activity or exertion; intervention indicated)<br><br>(Grade 4: Life-threatening consequences; urgent intervention indicated (e.g., continuous IV therapy or mechanical hemodynamic support))	Grade 0.<br><br>-If Grade 2 -- Hold study drug/study regimen dose until resolution to Grade 0. If toxicity rapidly improves to Grade 0, then the decision to reinitiate study drug/study regimen will be based upon treating physician's clinical judgment and after completion of steroid taper. If toxicity does not rapidly improve, permanently. Discontinue study drug/study regimen.<br><br>- If Grade 3-4, permanently discontinue study drug/study regimen.	-Consider using steroids if clinical suspicion is high.<br><br>For grade 2-4:<br>− Monitor symptoms daily, hospitalize.<br>− Promptly start IV methylprednisolone 2 to 4 mg/kg/day or equivalent after Cardiology consultation has determined whether and when to complete diagnostic procedures including a cardiac biopsy.<br>− Supportive care (e.g., oxygen).<br>− If no improvement within 3 to 5 days despite IV methylprednisolone at 2 to 4 mg/kg/day, promptly start immunosuppressive therapy such as TNF inhibitors (e.g., infliximab at 5 mg/kg every 2 weeks). Caution: It is important to rule out sepsis and refer to infliximab label for general guidance before using infliximab.<br>− Once the patient is improving, gradually taper steroids over ≥28 days and consider prophylactic antibiotics, antifungals, or anti-PJP treatment (refer to current NCCN guidelines for treatment of cancer-related infections)

Dosing Modification and Toxicity Management Guidelines for Immune-mediated, Infusion Related, and Non Immune-mediated Reactions for Durvalumab Monotherapy			
	**Grade of the Event (NCI CTCAE version 4.03)**	**Dose Modifications**	**Toxicity Management**
Myositis/Polymyositis ("Poly/myositis")	Any Grade	General Guidance	For Any Grade:<br>– Monitor patients for signs and symptoms of poly/myositis. Typically, muscle weakness/pain occurs in proximal muscles including upper arms, thighs, shoulders, hips, neck and back, but rarely affects the extremities including hands and fingers; also difficulty breathing and/or trouble swallowing can occur and progress rapidly. Increased general feelings of tiredness and fatigue may occur, and there can be new-onset falling, difficulty getting up from a fall, and trouble climbing stairs, standing up from a seated position, and/or reaching up.<br>– If poly/myositis is suspected, a Neurology consultation should be obtained early, with prompt guidance on diagnostic procedures. Myocarditis may co-occur with poly/myositis; refer to guidance under Myocarditis. Given breathing complications, refer to guidance under Pneumonitis/ILD. Given possibility of an existent (but previously unknown) autoimmune disorder, consider Rheumatology consultation.<br>– Consider, as necessary, discussing with the study physician.<br>– Initial work-up should include clinical evaluation,

Dosing Modification and Toxicity Management Guidelines for Immune-mediated, Infusion Related, and Non Immune-mediated Reactions for Durvalumab Monotherapy			
	**Grade of the Event (NCI CTCAE version 4.03)**	**Dose Modifications**	**Toxicity Management**
			creatine kinase, aldolase, LDH, BUN/creatinine, erythrocyte sedimentation rate or C-reactive protein level, urine myoglobin, and additional laboratory work-up as indicated, including a number of possible rheumatological/antibody tests (i.e., consider whether a rheumatologist consultation is indicated and could guide need for rheumatoid factor, antinuclear antibody, anti-smooth muscle, antisynthetase [such as anti-Jo-1], and/or signal-recognition particle antibodies). Confirmatory testing may include electromyography, nerve conduction studies, MRI of the muscles, and/or a muscle biopsy. Consider Barium swallow for evaluation of dysphagia or dysphonia.<br><br>Patients should be thoroughly evaluated to rule out any alternative etiology (e.g., disease progression, other medications, or infections).
	Grade 1<br>(mild pain)	For Grade 1:<br>-No dose modifications.	For Grade 1:<br>−Monitor and closely follow up in 2 to 4 days for clinical symptoms and initiate evaluation as clinically indicated.<br>−Consider Neurology consult.<br>−Consider, as necessary, discussing with the study physician.
	Grade 2<br>(moderate pain associated with	For Grade 2:<br>Hold study drug/study regimen dose until resolution to Grade ≤1.	For Grade 2:<br>−Monitor symptoms daily and consider hospitalization.<br>−Obtain Neurology consult, and initiate evaluation.

Dosing Modification and Toxicity Management Guidelines for Immune-mediated, Infusion Related, and Non Immune-mediated Reactions for Durvalumab Monotherapy			
	**Grade of the Event (NCI CTCAE version 4.03)**	**Dose Modifications**	**Toxicity Management**
	weakness; pain limiting instrumental activities of daily living [ADLs])	-Permanently discontinue study drug/study regimen if it does not resolve to Grade ≤1 within 30 days or if there are signs of respiratory insufficiency.	−Consider, as necessary, discussing with the study physician.<br>−If clinical course is rapidly progressive (particularly if difficulty breathing and/or trouble swallowing), promptly start IV methylprednisolone 2 to 4 mg/kg/day systemic steroids along with receiving input from Neurology consultant<br>−If clinical course is not rapidly progressive, start systemic steroids (e.g., prednisone 1 to 2 mg/kg/day PO or IV equivalent); if no improvement within 3 to 5 days, continue additional work up and start treatment with IV methylprednisolone 2 to 4 mg/kg/day<br>−If after start of IV methylprednisolone at 2 to 4 mg/kg/day there is no improvement within 3 to 5 days, consider start of immunosuppressive therapy such as TNF inhibitors (e.g., infliximab at 5 mg/kg every 2 weeks). Caution: It is important to rule out sepsis and refer to infliximab label for general guidance before using infliximab.<br>−Once the patient is improving, gradually taper steroids over ≥28 days and consider prophylactic antibiotics, antifungals, or anti-PJP treatment (refer to current NCCN guidelines for treatment of cancer-related infections).
	Grade 3 or 4 (pain associated with severe weakness; limiting self-care ADLs)	For Grade 3:<br>Hold study drug/study regimen dose until resolution to Grade ≤1.<br>Permanently discontinue study drug/study regimen if Grade 3 imAE does not resolve to Grade ≤1 within 30 days or if there are	For Grade 3 or 4 (severe or life-threatening events):<br>−Monitor symptoms closely; recommend hospitalization.<br>−Obtain Neurology consult, and complete full evaluation.<br>−Consider, as necessary, discussing with the study physician.<br>−Promptly start IV methylprednisolone 2 to 4 mg/kg/day systemic steroids along with receiving input from Neurology

Dosing Modification and Toxicity Management Guidelines for Immune-mediated, Infusion Related, and Non Immune-mediated Reactions for Durvalumab Monotherapy			
	**Grade of the Event (NCI CTCAE version 4.03)**	**Dose Modifications**	**Toxicity Management**
		signs of respiratory insufficiency.<br><br>For Grade 4:<br>-Permanently discontinue study drug/study regimen.	consultant.<br>−If after start of IV methylprednisolone at 2 to 4 mg/kg/day there is no improvement within 3 to 5 days, consider start of immunosuppressive therapy such as TNF inhibitors (e.g., infliximab at 5 mg/kg every 2 weeks). Caution: It is important to rule out sepsis and refer to infliximab label for general guidance before using infliximab.<br>−Consider whether patient may require IV IG, plasmapheresis.<br>−Once the patient is improving, gradually taper steroids over ≥28 days and consider prophylactic antibiotics, antifungals, or anti-PJP treatment (refer to current NCCN guidelines for treatment of cancer-related infections).

AE Adverse event; CTC Common Toxicity Criteria; CTCAE Common Terminology Criteria for Adverse Events; imAE immune-mediated adverse event; IV intravenous; NCI National Cancer Institute; PO By mouth.

**Dosing Modification and Toxicity Management Guidelines for Immune-mediated, Infusion Related, and Non Immune-mediated Reactions for Durvalumab Monotherapy**		
**Table 2- Infusion-Related Reactions**		
Severity Grade of the Event (NCI CTCAE version 4.03)	**Dose Modifications**	**Toxicity Management**
Any Grade	General Guidance	– Management per institutional standard at the discretion of investigator<br>– Monitor patients for signs and symptoms of infusion-related reactions (e.g., fever and/or shaking chills, flushing and/or itching, alterations in heart rate and blood pressure, dyspnea or chest discomfort, skin rashes etc.) and anaphylaxis (e.g., generalized urticaria, angioedema, wheezing, hypotension, tachycardia, etc.)
Grade 1	The infusion rate of study drug/study regimen may be decreased by 50% or temporarily interrupted until resolution of the event	For Grade 1 or Grade 2:<br>– Acetaminophen and/or antihistamines may be administered per institutional standard at the discretion of the investigator<br>– Consider premedication per institutional standard prior to subsequent doses<br>– Steroids should not be used for routine premedication of ≤Grade 2 infusion reactions
Grade 2	The infusion rate of study drug/study regimen may be decreased 50% or temporarily interrupted until resolution of the event<br>Subsequent infusions may be given at 50% of the initial infusion rate	
Grade 3/4	Permanently discontinue study drug/study regimen	For Grade 3 or 4:<br>Manage severe infusion-related reactions per institutional standards (e.g., IM epinephrine, followed by IV diphenhydramine and ranitidine, and IV glucocorticoid)

Dosing Modification and Toxicity Management Guidelines for Immune-mediated, Infusion Related, and Non Immune-mediated Reactions for Durvalumab Monotherapy		
**Table 3- Non-immune Mediated Reactions**		
**Severity Grade of the Event (NCI CTCAE version 4.03)**	**Dose Modification**	**Toxicity Management**
**Any Grade**	Note: dose modifications are not required for adverse events not deemed to be related to study treatment (i.e. events due to underlying disease) or for laboratory abnormalities not deemed to be clinically significant.	Treat accordingly as per institutional standard
**1**	No dose adjustment	Treat accordingly as per institutional standard
**2**	Hold study drug/study regimen until resolution to ≤ Grade 1 or baseline	Treat accordingly as per institutional standard
**3**	Hold study drug/study regimen until resolution to ≤ Grade 1 or baseline<br>For AEs that downgrade to ≤ Grade 2 within 7 days or resolve to ≤ Grade 1 or baseline within 14 days, resume study drug/study regimen administration. Otherwise, discontinue study drug/study regimen	Treat accordingly as per institutional standard
**4**	Discontinue Study drug/study regimen (Note for Grade 4 labs, decision to discontinue would be based on accompanying clinical signs/symptoms and as per Investigator's clinical judgment and in consultation with the sponsor)	Treat accordingly as per institutional standard

Abbreviations:

AChE = acetylcholine esterase; ADA = American Dietetic Association; AE = adverse event; ALP = alkaline phosphatase; ALT = alanine aminotransferase; AST = aspartate aminotransferase; CT = computed tomography; GI = gastrointestinal; IDS=Infectious Disease Service; ILD = interstitial lung disease; IM = intramuscular; irAE = immune-related adverse event; IV = intravenous; NCI CTCAE = National Cancer Institute Common Terminology Criteria for Adverse Events; PO = by mouth; TNF = tumor necrosis factor; TSH = thyroid stimulating hormone; ULN = upper limit of normal.

Weill Cornell Medicine

Human Research Protections Program
Immediate Report Form

The Immediate Reporting Policy that governs what you must report using this form is available at the following web address: http://researchintegrity.weill.cornell.edu/institutional_review_board/irb_policies_and_procedures.html. The policy spans 5 categories: Adverse Events/Unexpected Adverse Device Effects (Section A of this form), Other Risk Reporting (Section B of this form), Protocol Deviation Reporting (Section C of this form) and Other Compliance Reporting (Section D of this form).

If you have any questions, please email immediatereports@med.cornell.edu or call 646-962-8200.

DIRECTIONS:

(a) Fill out questions 1 through 8 on this page.

(b) Then choose the section on this form that corresponds to the type of Immediate Report you are making. Any given section you choose will have only 1 to 7 questions.

(c) Create a PDF of this form and attach the most current WCMC IRB consent document and any supplemental information.

(d) Sign this form with Principal Investigator signature on the last page and send to immediatereports@med.cornell.edu.

NOTE:

- If you are using the Clinical and Translational Science Center (CTSC) you must also CC your Immediate Report to ctscrsa@med.cornell.edu.
- If you are using the Weill Cornell Medical College Data Safety Monitoring Board (WCMC DSMB) you must also CC your Immediate Report to dsmb@med.cornell.edu.
- If your protocol is subject to the requirements of the Institutional Biosafety Committee (IBC), you must also CC your Immediate Report to submit2ibc@med.cornell.edu.

---

1. Principal Investigator:

2. Protocol #:

3. Protocol Title:

4. Brief One Sentence Description of Report (**This will be used to reference this specific submission in all IRB correspondence about this Immediate Report**):

5. Status of Protocol at WCMC Site ☐ Open ☐ Open, Closed to Subject Accrual ☐ Open, Data Analysis Only

6. Funding Source:

7. Does this protocol involve use of FDA-regulated product(s) including those that are FDA-approved? ☐ Yes ☐ No

a. If yes, is this protocol conducted under an IND or IDE? ☐ Yes ☐ No

b. If yes, name IND or IDE sponsor:

8. Check (and email this submission to) all that apply: I use the: ☐ WCMC Data Safety Monitoring Board (WCMC DSMB)

☐ WCMC Institutional Biosafety Committee (IBC) ☐ Clinical and Translational Science Center (CTSC)

A: ADVERSE EVENT REPORTING / UNANTICIPATED ADVERSE DEVICE EFFECTS

Tip: The goal of this section is to capture emergent risk information that has an impact on the rights and welfare of WCMC subjects and may also require compliance management.

1. I am reporting (check all that apply):

☐ Adverse Event that meets all 3 conditions as outlined in Section I(A) of the Immediate Reporting Policy http://researchintegrity.weill.cornell.edu/institutional_review_board/irb_policies_and_procedures.html.

☐ Adverse Event that requirements for reporting and a death or serious injury due to a defect of a Humanitarian Use Device, as outlined in Section I(C) of the Immediate Reporting Policy http://researchintegrity.weill.cornell.edu/institutional_review_board/irb_policies_and_procedures.html.

☐ Unanticipated adverse device effect as outlined in Section II(A) of the Immediate Reporting Policy http://researchintegrity.weill.cornell.edu/institutional_review_board/irb_policies_and_procedures.html. .

Describe the adverse event, its cause, and indicate any actions taken to date at the WCMC site to protect the rights and welfare of WCMC research subjects. Provide as much detail as possible. If the PI or any committee/board determines that the Immediate Report requires an amendment to the protocol or consent, the PI has 30 days from the date of making the Immediate Report (or release of the sponsor amendment) to submit a protocol amendment to the IRB. If this requirement cannot be met, a request for an extension must be made.

1. Please explain whether you believe this adverse event suggests that the research places WCMC subjects at a greater risk of harm than was previously known or recognized. If so, include what additional action you intend to take in your management of this change in risk. E.g., Documented contact with subjects by the PI or medically qualified co-I; amendment to protocol or consent).

2. Name any agencies or entities (E.g., FDA, NIH, sponsor, DSMB, etc.) that have been notified of this adverse event.

B: OTHER RISK REPORTING

Tip: The goal of this section is to capture emergent risk information that has an impact on the rights and welfare of WCMC subjects. If none of the below checkboxes apply, submit an acknowledgment submission instead of an Immediate Report.

1. I am reporting (check all that apply):

☐ An interim analysis, safety monitoring report, publication in the literature, or revised Investigator Brochure that indicates an increase in the frequency or magnitude of a given harm, uncovers a new risk, or provides more information about the benefits of the human research. **(PLEASE ATTACH THE ADDITIONAL DOCUMENTATION)**

☐ Change in FDA labeling or withdrawal from marketing of a drug, device, or biologic used in the human research protocol.

☐ Clinical Hold, Enrollment Hold (excluding planned holds for interim analysis), or Study Termination due to Emergent Risk Information

☐ Complaint of a participant that indicates participants or others might be at increased risk of harm or at risk of a new harm.

2. Please summarize the information and how you intend to manage the new risk posed to WCMC research participants. Include any actions taken to date.

C: PROTOCOL DEVIATIONS

Tip: The sponsor and the IRB are separate and distinct entities with different protocol deviation reporting requirements. Please consult with your sponsor to find out what protocol deviations you must submit to your sponsor. This form is meant to capture protocol deviations that must be reported to the IRB. Note that, i*f WCMC is the lead/coordinating site, then protocol deviation reports submitted from other sites must be submitted to the WCMC IRB.*

1. I am reporting (check all that apply):

☐ Protocol deviation that was made in order to eliminate an apparent immediate hazard to participants.

☐ Breach of Confidentiality

☐ Protocol deviation that represents a failure to follow the protocol or IRB policies and determinations due to the action or inaction of the investigator or research staff. (Exception: Rescheduling of research appointments due to holidays, vacations, accommodation of research subject, etc.) which meets both of the following conditions:

a. The deviation has the potential to negatively impact subject safety or integrity of study data (ability to draw conclusions from the study data), or affect the subject's willingness to participate in the study **AND**

b. The deviation places WCM subjects **at greater risk of harm** (including physical, psychological, economic or social harm).

2. Did this deviation occur at an external site (non-WCMC research site for which WCMC is the lead/coordinating site)?

☐ No ☐ Yes, answer (a)

a) ☐ What is the status of the research protocol at the non-WCMC research site?

☐ Open ☐ Open, Closed to Subject Accrual ☐ Open, Data Analysis Only

3. Date the Protocol Deviation Occurred:

4. Date the WCMC PI was notified:

5. Describe the protocol deviation and clearly state how this protocol deviation differs from what the IRB-approved protocol and/or IRB policy allows. Include why the deviation occurred. If the deviation occurred because of an error, please assess the root cause of that error.

6. Describe what actions have been taken to date to ensure the rights and welfare of the participant(s).

7. What preventative actions have been taken to ensure this deviation does not reoccur? Note: While some deviations occur due to human error, some represent systemic issues that need to be resolved. If the research staff will be more careful in the future, please help the IRB understand how the research staff will do this.

D: OTHER COMPLIANCE REPORTING

1. **I am reporting (check all that apply):**

☐ Finding of noncompliance or allegation of noncompliance

☐ Complaint of a participant that cannot be resolved by the research team.

☐ Incarceration of a participant in a protocol not approved to enroll prisoners.

2. Please describe the scenario in detail and indicate any actions taken to date:

WCMC Principal Investigator's Signature ___________________________________ Date: ___________________

IMPORTANT: Before providing your digital signature, combine this form and supporting documents into a single PDF.

Note on dates of receipt: In order to ensure the effective review of emergent risks to research subjects, the IRB cannot honor the received date of Immediate Report submissions with questions left blank or without the information requested. Immediate Reports are considered received as of the date this form is received with all questions on the form answered as instructed.

Certain adverse events and incidents must be immediately reported to NewYork-Presbyterian Patient Services, Risk Management, and/or the Department of Health. Please refer to the following policies/offices to determine whether this event qualifies for reporting to any of the aforementioned groups.

Risk Management Incident Reporting (x66180): http://weill.cornell.edu/risk-management/insurance/incident_reporting.html
SAEs/Sentinel Events Reporting: https://infonet.nyp.org/QA/HospitalManual/S120SeriousAdverseEvents.pdf
NYPH Patient Services 212-746-4293
NYPH Westchester Patient Services 917-997-5920
