# Supplementary material for: Neoadjuvant durvalumab plus radiation versus durvalumab alone in stages I–III non-small cell lung cancer: survival outcomes and molecular correlates of a randomized phase II trial
Source: Nat Commun. 2023 Dec 19;14:8435. doi: 10.1038/s41467-023-44195-x (PMC10730562; doi:10.1038/s41467-023-44195-x)
Supplement: Supplementary file 3 — Description of Additional Supplementary Files [file 41467_2023_44195_MOESM3_ESM.pdf]

Description of Additional Supplementary Files

Supplementary Data 1 – Gene set.
