# Supplementary material for: Neoadjuvant durvalumab plus radiation versus durvalumab alone in stages I–III non-small cell lung cancer: survival outcomes and molecular correlates of a randomized phase II trial
Source: Nat Commun. 2023 Dec 19;14:8435. doi: 10.1038/s41467-023-44195-x (PMC10730562; doi:10.1038/s41467-023-44195-x)
Supplement: Supplementary file 5 — Reporting Summary [file 41467_2023_44195_MOESM5_ESM.pdf]

nature portfolio

Corresponding author(s): Nasser Altorki

Last updated by author(s): November 25,2023

Reporting Summary

Nature Portfolio wishes to improve the reproducibility of the work that we publish. This form provides structure for consistency and transparency in reporting. For further information on Nature Portfolio policies, see our Editorial Policies and the Editorial Policy Checklist.

Statistics

For all statistical analyses, confirm that the following items are present in the figure legend, table legend, main text, or Methods section.

n/a	Confirmed	
☐	☒	The exact sample size (*n*) for each experimental group/condition, given as a discrete number and unit of measurement
☐	☐	A statement on whether measurements were taken from distinct samples or whether the same sample was measured repeatedly
☐	☒	The statistical test(s) used AND whether they are one- or two-sided<br>*Only common tests should be described solely by name; describe more complex techniques in the Methods section.*
☐	☒	A description of all covariates tested
☐	☒	A description of any assumptions or corrections, such as tests of normality and adjustment for multiple comparisons
☐	☒	A full description of the statistical parameters including central tendency (e.g. means) or other basic estimates (e.g. regression coefficient) AND variation (e.g. standard deviation) or associated estimates of uncertainty (e.g. confidence intervals)
☐	☒	For null hypothesis testing, the test statistic (e.g. *F*, *t*, *r*) with confidence intervals, effect sizes, degrees of freedom and *P* value noted<br>*Give P values as exact values whenever suitable.*
☐	☐	For Bayesian analysis, information on the choice of priors and Markov chain Monte Carlo settings
☐	☒	For hierarchical and complex designs, identification of the appropriate level for tests and full reporting of outcomes
☒	☐	Estimates of effect sizes (e.g. Cohen's *d*, Pearson's *r*), indicating how they were calculated

Our web collection on statistics for biologists contains articles on many of the points above.

Software and code

Policy information about availability of computer code

Data collection	For flow-cytometry analyses, all data were collected on a 5-laser (UV-V-B-YG-R) Cytek Aurora spectral flow cytometer (Cytek) using staining procedures as described below. Raw FCS files generated from this were used for unbiased and targeted analyses.
Data analysis	For flow-cytometry analyses, we performed unbiased dimensionality reduction and clustering using PacMap. These analyses were supplemented with initial findings from PacMap and additional analyses using prior knowledge on the biology of T cells during cancer immunotherapies. Analysis were performed in Prism Graph pad software (version 9.02), R (version 4.0.2 (Copyright [C] 2020; The R Foundationfor Statistical Computing Vienna, Austria) and SAS Version 9.4 (SAS Institute, Inc., Cary, North Carolina).

For manuscripts utilizing custom algorithms or software that are central to the research but not yet described in published literature, software must be made available to editors and reviewers. We strongly encourage code deposition in a community repository (e.g. GitHub). See the Nature Portfolio guidelines for submitting code & software for further information.

Data

Policy information about availability of data

All manuscripts must include a data availability statement. This statement should provide the following information, where applicable:

- Accession codes, unique identifiers, or web links for publicly available datasets
- A description of any restrictions on data availability
- For clinical datasets or third party data, please ensure that the statement adheres to our policy

The author confirms that all data generated or analysed during this study are included in this published article. Additional information is available upon request from the corresponding authors

Research involving human participants, their data, or biological material

Policy information about studies with human participants or human data. See also policy information about sex, gender (identity/presentation), and sexual orientation and race, ethnicity and racism.

Reporting on sex and gender	In this secondary analysis of our trial, 28 males and 24 females were recruited and treated as per protocol.
Reporting on race, ethnicity, or other socially relevant groupings	Patients race was self-reported. 40 of 52 patients were White, 7 Blacks and 5 Asians
Population characteristics	In this secondary analysis , 52 patients were included (26 in each group), median age 70yrs, 24 females and 28 males,40 self identified whites 7 blacks and 5 Asians. Patients had non-small cell lung cancer stages 1-3. All covariates are shown in Tables 1 and 3
Recruitment	Lung cancer clinics at Weill Cornell Medicine Medical Center. Bias was minimized by randomization. Block randomization was generated by the study statistician . All block sizes were concealed from investigators and study personel and were only known by study statistician
Ethics oversight	The trial protocol was approved by the Institutional Review Board of Weill Cornell Medicine and the New York Presbyterian Hospital (protocol number:15010157950), and the trial was monitored by the Weill Cornell Medicine Data Safety Monitoring Board. Randomization minimized bias that might have impacted results.

Note that full information on the approval of the study protocol must also be provided in the manuscript.

Field-specific reporting

Please select the one below that is the best fit for your research. If you are not sure, read the appropriate sections before making your selection.

☒ Life sciences ☐ Behavioural & social sciences ☐ Ecological, evolutionary & environmental sciences

For a reference copy of the document with all sections, see nature.com/documents/nr-reporting-summary-flat.pdf

Life sciences study design

All studies must disclose on these points even when the disclosure is negative.

Sample size	52 patients, 26 in the durvalumab alone group and 26 in the durvalumab plus SBRT group; patients with available PBMC specimens were included
Data exclusions	None
Replication	N/A. In this clinical trial, clinical samples were limited
Randomization	Patients were randomly assigned to treatment groups. For analysis of blood and gene expression, there was no randomization. Analysis of gene expressionand blood was an unplanned post-hoc exploratory analysis. pre and post treatment samples served as internal controls. Group sizes too small to subdivide by multiple covariates
Blinding	There was no blinding performed in the clinical trial or in the analysis of gene expression or blood samples.

Reporting for specific materials, systems and methods

We require information from authors about some types of materials, experimental systems and methods used in many studies. Here, indicate whether each material, system or method listed is relevant to your study. If you are not sure if a list item applies to your research, read the appropriate section before selecting a response.

Materials & experimental systems

n/a	Involved in the study	
☐	☒	Antibodies
☒	☐	Eukaryotic cell lines
☒	☐	Palaeontology and archaeology
☒	☐	Animals and other organisms
☐	☒	Clinical data
☒	☐	Dual use research of concern
☒	☐	Plants

Methods

n/a	Involved in the study	
☒	☐	ChIP-seq
☐	☒	Flow cytometry
☒	☐	MRI-based neuroimaging

Clinical data

Policy information about clinical studies

All manuscripts should comply with the ICMJE guidelines for publication of clinical research and a completed CONSORT checklist must be included with all submissions.

Clinical trial registration	Clinical trials.gov NCT02904954
Study protocol	supplementary material
Data collection	Lung Cancer clinics at tertiary care academic medical center New York,USA between January 2017-and September 2020
Outcomes	Primary outcomne: pathological response (previously reported, Lancet Oncology; VOLUME 22, ISSUE 6, P824-835, JUNE 2021) secondary outcome: disease free survival. Pathological response assessed by pulmonary pathologist and DFS by Kaplan Meir method.

Flow Cytometry

Plots

Confirm that:

- [x] The axis labels state the marker and fluorochrome used (e.g. CD4-FITC).
- [x] The axis scales are clearly visible. Include numbers along axes only for bottom left plot of group (a 'group' is an analysis of identical markers).
- [x] All plots are contour plots with outliers or pseudocolor plots.
- [ ] A numerical value for number of cells or percentage (with statistics) is provided.

Methodology

Sample preparation

Following overnight culture all PBMC samples were collected in 5 mL Eppendorf tubes. Samples were centrifuged at 400 x g for 5 minutes, washed once with ice-cold PBS, and transferred to 96-well plates for staining. All samples were resuspended in 100 ul 1:500 Zombie-NearIR (Biolegend) in PBS and stained for 15 minutes on ice in the dark. Samples were then washed once with ice-cold FACS buffer (PBS with 2% FBS and 2 mM EDTA; Gibco), blocked for 10 minutes with 1:20 Human TruStain FcX (Biolegend) in FACS buffer, and then stained with surface antibodies in FACS buffer supplemented with 10 uL Brilliant Stain Buffer Plus (BD) (100 ul total volume per sample) for 30 minutes on ice in the dark. Samples were washed twice with FACS buffer and resuspended in 200 uL 1X FoxP3 Fixation/Permeabilization Buffer (eBioscience) for 30 minutes in the dark. Each sample was then washed twice with 1X Permeabilization Buffer (eBioscience) and stained with intracellular antibodies in 1X Permeabilization Buffer supplemented with 10 uL Brilliant Stain Buffer Plus (100 ul total volume per sample) for 30 minutes in the dark. Samples were washed once with 1X Permeabilization Buffer, once with FACS buffer, resuspended in 200 ul FACS buffer and filtered through 70 um filter-cap FACS tubes, and acquired on a 5-laser (UV-V-B-YG-R) Cytek Aurora spectral flow cytometer (Cytek). UltraComp eBeads (Thermo Fisher) were used for single stain compensation controls. Fluorescence-minus-one controls were prepared for intracellular cytokine and transcription factor stains. All analysis of flow cytometry data was performed in FlowJo v10.8 (BD). For quality control, PBMC samples with <10% live cells or <2000 total live cells were excluded from final analyses.

Below are the antibdoies, detection channel, and manufacturer with product identification.

CD8 BV421 Biolegend 344748
HLA-DR eF450/PacBlue/DAPI/BFP Thermo 48-9956-42
CD4 BV480/BUV496 BD 566104
Ki67 eF506 Invitrogen 69-5698-82
CD95 BV510 Biolegend 305640
LAG3 BV605/SB600/A594 BD 745160
CD137 (4-1BB) BV650/SB645/Qdot655 Biolegend 309828
CD45RA BV711/SB702/Qdot705 Biolegend 304137
CD28 BV750 Biolegend 302969
CD103 BV785/SB780 BD 568274
TCF1 A488/FITC/KV520/CFSE/GFP Cell Signaling 6444S
CD39 PE/A546/NB550 Biolegend 328208
FoxP3 PE-CF594/PE-Dazzle 594/NB610/BB630 Biolegend 320125
CD27 PE-Cy5 Thermo 15-0279-42
KLRG1 PerCP-Cy5.5/PE-Cy5.5/PE-Fire700 Biolegend 367708
TIM3 PE-Cy7 eBioscience 25-3109-42
TOX1 APC Miltenyi 130-118-335
NKG2D APC-Cy5.5/A660/SN685/NR685 Biolegend 320842
CD3 A700/APC-R700/NR700 Biolegend 300324
CCR7 APC-Cy7/APC-eF780/APC-Fire750/LD-NIR Biolegend 353246
PD1 APC-Fire810/BUV805/A790 Biolegend 612620

Instrument

5-laser (UV-V-B-YG-R) Cytek Aurora spectral flow cytometer (Cytek)

Software	23-marker spectral flow analysis and established dimensionality reduction and clustering tools
Cell population abundance	Cell population abundances were determined as fractions (given in percentages) of parent populations, as indicated in each specific analysis.
Gating strategy	Cells were gated on forward and side scatter to exclude doublets and debris; the next gate was on Zombie-NIR negative cells identifying viable cells only, then on CD3 to define T lymphocytes; CD4 and CD8 to define respective T cell populations followed by more granular phenotyping as indicated in specific analyses.

☒ Tick this box to confirm that a figure exemplifying the gating strategy is provided in the Supplementary Information.
